# Development of an HPLC–HRMS Method for the Identification and Semi‐Quantitation in River Water Samples of the Transformation Products Originated From Heterogeneous Photocatalysis of Antipsychotic Drugs

**DOI:** 10.1002/jssc.70299

**Published:** 2025-10-14

**Authors:** Elena De Rosa, Serena Arpaia, Sandra Vietti Michelina, Francesco Oliva, Claudio Medana, Paola Calza, Federica Dal Bello

**Affiliations:** ^1^ Department of Molecular Biotechnology and Health Science University of Turin Torino Italy; ^2^ Department of Clinical and Biological Sciences University of Turin Torino Italy; ^3^ Department of Chemistry University of Turin Torino Italy

**Keywords:** aripiprazole, haloperidol, high‐performance liquid chromatography–high‐resolution mass spectrometry (HPLC–HRMS), river surface water, titanium dioxide, toxicity test, transformation products

## Abstract

The environmental occurrence of psychiatric drugs is a growing concern due to their widespread use and persistence in aquatic systems. In this study, haloperidol and aripiprazole, two commonly prescribed antipsychotic agents, were selected as model compounds to investigate their photocatalytic modification and assess the formation of transformation products (TPs). A high‐performance liquid chromatography–high‐resolution mass spectrometry (HPLC–HRMS) method was developed for the identification and structural elucidation of TPs generated via TiO_2_‐mediated heterogeneous photocatalysis in ultrapure water. Reverse‐phase chromatographic separation was achieved using an octadecyl silica column as the stationary phase, with formic acid and acetonitrile as the mobile phase. The total run time of 37 min provided adequate resolution for the separation of the main TPs isomers. TPs annotation was carried out using orbitrap technology, operated at a resolving power of 60 000 for all experiments. Including isomers, a total of 32 haloperidol TPs and 13 aripiprazole TPs were identified, with proposed fragmentation pathways established through targeted MS*^n^* experiments. The method was then applied to the analysis of real river surface water samples collected from the Po and Sangone Rivers in Northern Italy over a 3‐month period. Following solid‐phase extraction (SPE), both parent compounds and TPs were recognized and semi‐quantified by retention time, exact mass, and MS^2^ spectral data. Haloperidol and aripiprazole were detected at maximum concentrations of 27 and 67 ng L^−1^, respectively. To assess the potential biological impact, in vitro cytotoxicity tests were conducted on normal (BEAS‐2B) and oncogenic (BEAS G12C) human bronchioalveolar epithelial cell lines. Although the parent drugs exhibited negligible toxicity at the tested concentrations, haloperidol TPs induced marked cytotoxic effects in both cell models. These results highlight the necessity of including TPs in environmental monitoring and toxicological assessments.

## 1 Introduction

From 1990 to 2019, the global incidence of schizophrenia increased by over 37% [1]. In Italy, between 2015 and 2022, an average of 795 000 psychiatric patients were treated by specialists annually, with 37% diagnosed with schizophrenia or other functional psychoses. Consequently, the use of antipsychotic medications has been steadily rising. A recent global study across 64 countries reported a 58% increase in atypical antipsychotic use between 2014 and 2019, particularly in high‐income nations, reflecting a broader trend in the growing reliance on these medications worldwide [2].

The antipsychotic action of neuroleptics has been predominantly attributed to the reduction of dopamine effects in the nervous system [3]. The first‐generation, or typical, antipsychotics act by inducing postsynaptic blockade of dopamine D2 receptors in the mesolimbic pathway of the central nervous system [4]. However, their low specificity results in unintended blockade of these receptors in other dopaminergic pathways, leading to extrapyramidal side effects (nigrostriatal pathway) [5], hyperprolactinemia (tuberoinfundibular pathway) [6], and worsening of negative symptoms (mesocortical pathway) [7]. Second‐generation, or atypical, antipsychotics have fewer side effects than typical antipsychotics due to their intrinsic serotonin receptor antagonism and transient D2 receptor binding. However, their broad pharmacological profile can lead to additional side effects, such as weight gain and cardiometabolic issues due to antihistaminic activity and anhidrosis, constipation, and urinary retention due to anticholinergic effects [7]. Third‐generation antipsychotics act as dopamine modulators due to their high affinity for D2 receptors and low intrinsic agonist activity (partial agonist). These characteristics make them ideal candidates for balancing dopaminergic activity while minimizing side effects [8]. We focused our attention on two antipsychotic drugs largely used, haloperidol and aripiprazole, which are prototypes of the first‐generation and third‐generation antipsychotic classes, respectively [9, 10].

In recent decades, the fate, occurrence, and effects of pharmaceutical pollutants in the aquatic environment have been assessed [11, 12, 13]. Originating from urban, industrial, and agricultural sources, these substances can enter water systems and are often not completely removed by conventional water treatment plants. Although their long‐term impact is not yet fully understood, emerging contaminants pose a serious threat, as many of them can accumulate in organisms and cause toxic and persistent effects.

Haloperidol and aripiprazole are nowadays recognized as environmental pollutants with potential risks to human health through the food chain. The drug is excreted largely unmetabolized in urine and feces and is poorly removed by standard wastewater treatment. Environmental contamination stems from improper disposal, hospital waste, livestock runoff, and incomplete sewage treatment. It has been detected globally, for example, up to 10 µg L^−1^ in South Africa's Msunduzi River [14], and at 39–2690 µg L^−1^ in hospital sewage and wastewater effluents [15].

In the environment, haloperidol may persist due to its strong affinity for particulates in air (vapor pressure 4.85 × 10^−11^ mm Hg at 25°C), low mobility and volatilization in soil (Henry's constant 2.3 × 10^−14^ atm m^3^ mol^−1^), and tendency to adsorb to solids in water, where it mainly exists in ionized form (p*K*a 8.66) with moderate bioconcentration potential (BCF 59) [16]. Aquatic organisms, especially fish, can accumulate haloperidol, risking behavioral and physiological disruption even at low concentrations due to the drug's potency. This raises concerns for human exposure via the food chain. A study on juvenile African sharptooth catfish (*Clarias gariepinus*) reported an LC_50_ of 2.40 mg L^−1^ after 96 h of exposure [17].

Regarding aripiprazole, concentrations up to 1220 ng L^−1^ have been detected in wastewater treatment plant (WWTP) effluents and up to 1490 ng g^−1^ in sludge [18]. Even at low levels, aripiprazole poses risks to aquatic life, as zebrafish studies showed impaired stress responses and anti‐predator behavior at concentrations as low as 0.556 ng L^−1^ [19, 20], potentially affecting survival and ecosystem dynamics. Aripiprazole is environmentally persistent with high bioaccumulation potential (BCF 1500). It binds to particulates in air (vapor pressure 3.2 × 10^−13^ mm Hg), adsorbs to soil organic carbon (p*K*a 7.46), and associates with suspended solids and sediments in water, whereas its chromophores enable photolysis by sunlight [21].

A promising approach for environmental remediation is the implementation of advanced oxidation processes (AOPs) in treatment plants [22]. AOPs rely on the generation of highly reactive oxygen species (ROS) to degrade contaminants, and within this framework, photocatalysis represents an environmentally friendly option for pollutant removal. In this contest, titanium dioxide (TiO_2_) is among the most commonly used and effective metal oxide [23, 24, 25, 26]. During the degradation of potentially toxic parent compounds by TiO_2_, transformation products (TPs) may form, and their toxicity should also be evaluated. The widespread presence of haloperidol and aripiprazole in the environment, especially in aquatic systems, has highlighted the need for in‐depth studies investigating not only the toxicity and the environmental fate of the original molecules, but also of their metabolites and TPs formed through natural processes.

In the environment, like many other drugs, both haloperidol and aripiprazole can undergo chemical degradation processes, including photolysis, hydrolysis, and microbial (bacterial) degradation [27].

Currently, there is no experimental toxicity data available for the photocatalytic TPs of haloperidol or aripiprazole and their occurrence in the environment. For haloperidol, oxidative and photolytic studies have identified *cis*‐ and *trans‐haloperidol N‐oxide*, with in silico assessments suggesting low toxicological concern; however, no experimental validation has been reported [28]. For aripiprazole, photocatalytic degradation products have not yet been identified, and no related toxicity data are available. Furthermore, qualitative and quantitative data for these TPs obtained with heterogeneous photocatalysis mediated by semi‐conductors are lacking for both compounds.

The aim of the present research study was the development of a high‐performance liquid chromatography–high‐resolution mass spectrometry (HPLC–HRMS) method for the determination of TiO_2_‐mediated photocatalysis TPs of the antipsychotic drugs haloperidol and aripiprazole. HPLC–HRMS was selected due to its high sensitivity, accurate mass measurements, and ability to provide structural information essential for the characterization of unknown TPs [29, 30, 31, 32, 33, 34, 35]. For the TPs of haloperidol and aripiprazole, the work involved the hypothesis of chemical structures and fragmentation pathways through advanced spectrometric analysis. The methodology was then applied to real river surface water samples for the research of parent molecules and annotated TPs. The protocol of sample preparation was focused on the development of a procedure for the analysis of aqueous samples, ensuring sufficient sensitivity to detect even the lowest concentrations of TPs present in real matrices.

Finally, the toxicity of haloperidol and aripiprazole, as well as their TPs, was assessed using cell viability assays on both normal (BEAS 2B) and oncogenic (BEAS 2B G12C) human lung epithelial cell lines. Toxicity analysis is used to determine the potential health risks associated with an acute/chronic exposure to these drugs and their TPs.

## 2 Materials and Methods

### 2.1 Chemicals

Haloperidol, aripiprazole, and ketoprofen d3 analytical standards (purity >98%), together with formic acid, were provided by Merck (Milan, Italy). The stock solutions of haloperidol and aripiprazole were prepared with a final concentration of 20 mg L^−1^ in methanol and ethanol, respectively. For the heterogeneous photocatalysis, a white titanium dioxide powder (Evonik P25) was used. The solvents used in HPLC were acetonitrile (HiPerSolv Chromanorm, purity ≥99.9%, VWR Chemicals, Milan, Italy) and a 0.1% aqueous solution of formic acid (Merck, Milan, Italy).

### 2.2 Photocatalytic Procedures

Considering the low solubility of the drugs in water, haloperidol and aripiprazole were solubilized in alcoholic solutions and subsequently removed using a rotavapor (R‐100, BUCHI, Switzerland). Titanium dioxide powder (400 mg L^−1^) was added to the drugs obtaining a final drug concentration of 10 mg L^−1^. The suspension obtained was placed in a Pyrex glass flask with a frosted neck for rotavapor, and the solvent was removed. The drug adsorbed on TiO_2_ was then resuspended in water and sonicated for 30 min. For the photocatalysis experiments, a Solarbox (CO.FO.ME.GRA., Milan, Italy) equipped with a 1500 W xenon lamp and cutoff filter at 340 nm was used. Several aliquots were placed in cells to achieve the photodegradation at the following time points: 0, 5, 10, 15, 30, 60, and 120 min. All the suspensions were filtered through a 0.45 µm polypropylene syringe filter (VWR, Milan, Italy) before the HPLC–HRMS analysis.

### 2.3 River Water Sampling Procedure

River sampling was carried out once a month in June, July, and August 2024 (wet season), and the two drugs and their TPs were quantified (weather conditions during the sampling months are depicted in Table S1). The environmental water samples were collected from Po River (Turin, 45.02430° N, 7.41037° E) and Sangone River (Moncalieri, 45.01345° N, 7.67007° E and Forno di Coazze, 45.03164° N, 7.23490° E). Samples were immediately filtered using a vacuum system and subsequently stored in the dark at 4°C for up to 24 h to prevent spontaneous compound degradation. Solid‐phase extraction (SPE) protocol using HLB cartridge (Merck, Milan, Italy) was performed to extract the analytes from water samples. Each cartridge was conditioned with methanol and water, and then 500 mL of sample was loaded followed by a washing step with water. Sample was eluted with methanol, evaporated to dryness overnight in a CentriVap lyophilizer (Labconco Co., Kansas City, MO, USA), and finally reconstituted with 500 µL of methanol/water 10:90, achieving a concentration factor of 2500. We used as working and injection standard ketoprofen‐d3 (final concentration of 1 mg L^−1^) to monitor the performance of the SPE protocol and MS analysis. Semi‐quantitative analysis of molecules was obtained using an external calibration curve of haloperidol and aripiprazole. A 1000 mg L^−1^ standard solution of analytical standards was made in methanol, whereas the diluted solutions in water/methanol 8/2 at concentration ranged from 1 to 750 ng L^−1^. Recovery of the antipsychotic drugs was assessed by applying the SPE protocol to 500 mL of pooled river surface water, obtained by combining 250 mL from two of the three sampling sites (total available volume: 750 mL), spiked with haloperidol and aripiprazole (final concentration 500 mg L^−1^).

### 2.4 Analytical Procedures

A Dionex UltiMate 3000 Basic Automated System chromatographer coupled through an ESI source to an Orbitrap Fusion Tribrid Mass Spectrometer (Thermo Fisher Scientifics, Bremen, Germany) was used for the analysis of parent compounds and TPs. The chromatographic separation was obtained with a C18 column (Phenomenex Luna C18(2), 150 × 2 mm^2^, 3 µm) thermostated at 40°C, with an injection volume of 20 µL in full loop mode.

The mobile phase involved the use of acetonitrile as the organic modifier (solvent B) and Milli‐Q water acidified with 0.1% formic acid (solvent A). The mobile phase flow rate is 200 µL min^−1^, and the gradient was as follows: from 5% to 35% B in 25 min, up to 100% in 3 min; then the column came back to the initial conditions for a total run time of 37 min.

The LC mobile phase was delivered to the ESI source using nitrogen both as sheath and auxiliary gas. Source parameters were set as follows: sheath gas 35 arbitrary units (arb), auxiliary gas 20 arb, ion transfer tube temperature 300°C, and CID collision energy 26%. Full mass spectra were acquired in positive ion mode in the *m*/*z* range between 60 and 600, and dedicated MS*^n^* experiments for analytes and TPs were subsequently carried out (resolving power of 60k for all the experiments).

### 2.5 Cellular Model and Growth Conditions

BEAS 2B cells (human bronchial epithelial cells derived from noncancerous individuals) were obtained from ATCC (catalog number: CRL‐3588). BEAS 2B KRAS G12C (oncogenic) cell model was obtained through stable retrovirus infection of BEAS 2B. The growing condition was 37°C with 5% of CO_2_ in Thermo Scientific cell incubator, using Dulbecco's modified Eagle medium (DMEM) with the addition of 10% of fetal bovine serum (FBS) and 1% of antibiotic cocktail (penicillin–streptavidin) as cultures media.

### 2.6 Site‐Directed Mutagenesis and Virus Infection

pBABE HA‐tagged KRAS G12C plasmid was obtained by point mutagenesis with Agilent Technologies QuikChange XL Site‐Directed Mutagenesis Kit (Cat #200516‐5) starting from pBABE HA‐tagged KRAS WT (a gift from Channing Der, Addgene plasmid #75282). Retroviruses were generated by co‐transfection of pBABE plasmids together with pAmpho and VSVG plasmids into 293T cells using the Effectene Transfection Reagent kit (Quiagen ref: 301427). The retroviruses were transduced into BEAS 2B (0.5–1 × 10^5^) followed by puromycin selection (1 µg mL^−1^).

### 2.7 Cellular Toxicity Test Sample Preparation

A Milli‐Q water suspension of TiO_2_ and standard solutions of selected antipsychotic drugs were prepared. A volume of 2.5 mL of TiO_2_ suspensions was placed in a Pyrex cell with 2.5 mL of Milli‐Q water as control and drugs. Then they were photocatalyzed through a Solarbox (CO.FO.ME.GRA., Milan, Italy) equipped with a 1500 W xenon lamp and a cutoff filter at 340 nm. Several time points were collected (0, 10, and 15 min). All the suspensions were filtered through a 0.45 µm polypropylene syringe filter (VWR, Milan, Italy) before the second filtration with 0.2 µm filter membrane (Cytiva, Whatman GD/X 25, polypropylene) in sterile conditions to be used in toxicity test.

### 2.8 Toxicity Test

A total of 1000 cells/well were plated in 96‐well plate (Falcon ref#353072). The following day they were treated with the photocatalytic product, filtered with 0.2 µm membrane in sterile biological environment, and incubated for 3 days. Sterile Milli‐Q water was used as control treatment. Then the cell viability was evaluated with CellTiter‐Glo 2.0 (Promega #G9242) and the plate reader Glomax Discover (Promega GM3000). The graphs and the anti‐proliferative effect (compounds’ half maximal inhibitory concentration, IC50) calculation were done with the prism V10.0 (Prism software).

## 3 Results and Discussion

### 3.1 Photocatalytic Degradation

**FIGURE 1:**
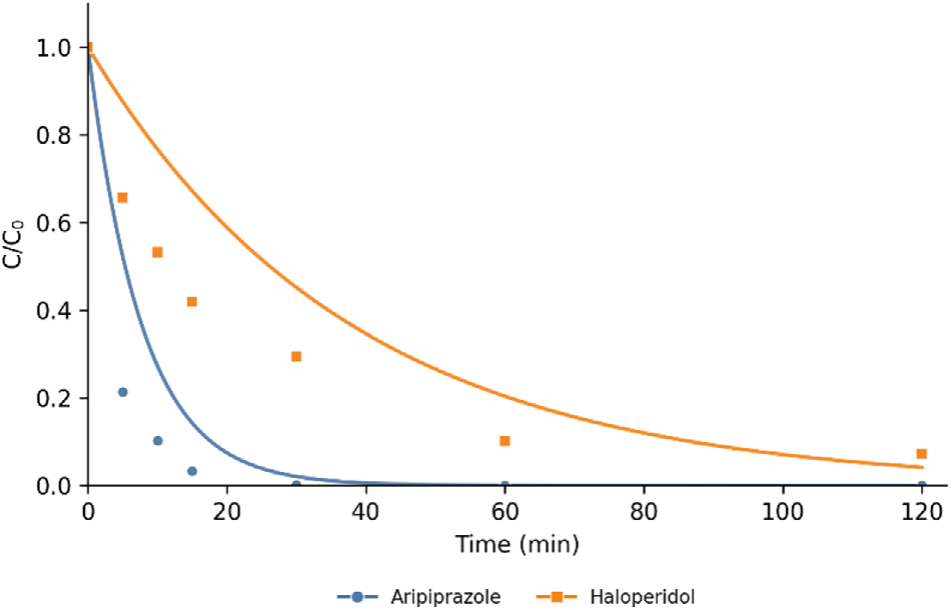
Haloperidol (orange line) and aripiprazole (blue line) kinetics of photocatalytic degradation.

From the disappearance curve of the drugs, a nearly complete elimination after 120 min was observed for haloperidol, whereas for aripiprazole, the complete elimination was achieved in 30 min (Figure 1).

Furthermore, it is essential to consider that the application of AOPs for the removal of these pharmaceuticals may lead to the formation of by‐products, which must be identified, quantified, and evaluated for potential biological activity. To this end, an HPLC–HRMS method was developed for the identification of haloperidol and aripiprazole TPs. The use of a high‐resolution separation technique, such as HPLC, is crucial for resolving the complex mixtures resulting from TiO_2_ photocatalysis, which contain both parent compounds and their TPs [36, 37, 38]. Appropriate retention times for the parent compounds are essential to avoid band broadening and to ensure the resolution of TPs without coelution. To achieve this, gradient speed tests were performed. The final gradient conditions yielded retention times of 21.35 and 25.46 min for haloperidol (*m*/*z* 376.1474) and aripiprazole (*m*/*z* 448.1553), respectively. The combination of the HPLC method with the developed HRMS approach enabled the tentative identification of the chemical structures of the TPs. The resolving power of 60 000 for both full‐scan and MS*^n^* spectra ensured mass accuracy up to the third decimal place in *m*/*z* measurements. A thorough understanding of the fragmentation pathways underlying MS*^n^* spectra was essential to hypothesize the structures of the detected TPs. We followed the rules proposed in literature by Shymanski [39] to tentatively propose the chemical structure of TPs. All the proposed structures are Level 3 recognition.

The photocatalytic degradation process resulted in the formation of 32 TPs for haloperidol and 13 TPs for aripiprazole. Kinetic profiles of TPs formation were evaluated, showing maximum signal intensities after 10–15 min of photocatalysis.

**FIGURE 2:**
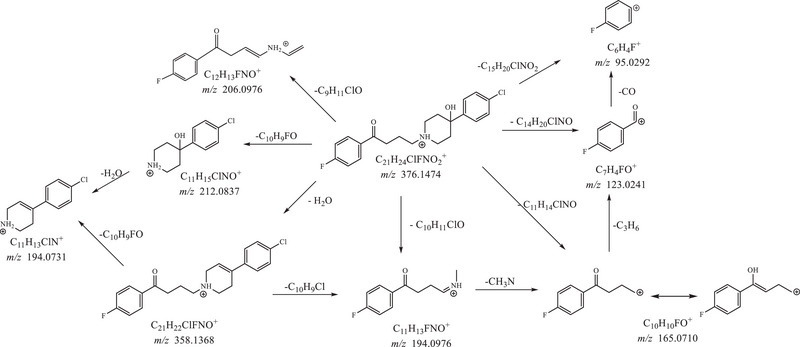
MS*^n^* fragmentation pathway of haloperidol.

**FIGURE 3:**
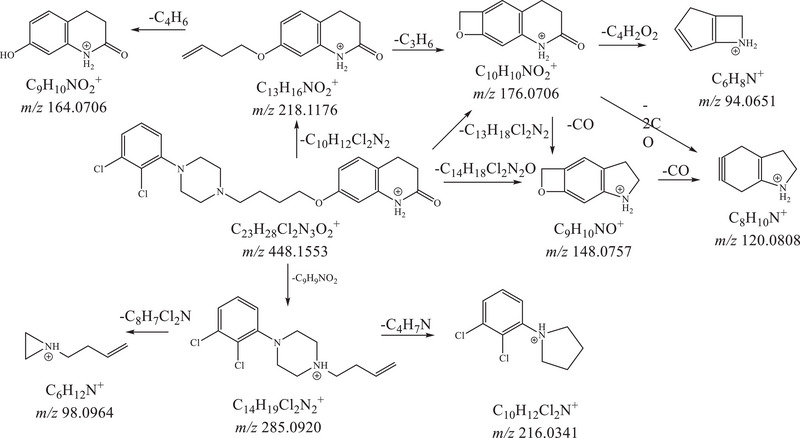
MS*^n^* fragmentation pathway of aripiprazole.

Flow injection analysis (FIA) of parent molecules was performed to obtain MS*^n^* fragmentation spectra leading to hypothesize for each ion: the formulae, the corresponding neutral loss, the mass accuracy, and the relative intensity (Tables 1 and 2). The fragmentation pathway of the parent compounds (Figures 2 and 3) represents a fundamental starting point for elucidating the chemical structures of the TPs. Identifying the main neutral losses that generate the most abundant product ions is key to localizing modifications such as hydroxylation, reduction, or other chemical transformations within the molecular structure.

**TABLE 1:** List of MS*^n^* for haloperidol, *m*/*z* 376.1466.

[MH]^+^	tr (min)	Δppm	MS^2^, molecular formula, (abundance %), [loss]	Δppm	MS^3^, molecular formula, (abundance %), [loss]	Δppm
376.1466 [C_21_H_24_ClFNO_2_]⁺	21.35	−2.2	358.1363 [C_21_H_22_ClFNO]⁺, (59) [–H_2_O]	−1.5	194.0728 [C_11_H_13_NCl]⁺, (5), [C_10_H_9_FO]	−1.6
			212.0834 [C_11_H_15_ClNO]⁺, (0,6), [–C_10_H_9_FO]	−1.3	∖	∖
			206.0972 [C_12_H_13_FNO]⁺, (0,5), [–C_9_H_11_ClO]	−1.8	∖	∖
			194.0973 [C_11_H_13_FNO]⁺, (2), [–C_10_H_11_ClO]	−1.4	165.0712 [C_10_H_10_FO]⁺, (100), [–CH_3_N]	1.1
			194.0728 [C_11_H_13_NCl]⁺, (5), [C_10_H_9_FO]	−1.6	∖	∖
			165.0712 [C_10_H_10_FO]⁺, (100), [–C_11_H_14_ClNO]	1.1	123.0242 [C_7_H_4_FO]⁺, (100), [–C_3_H_6_]	1.1
			123.0242 [C_7_H_4_FO]⁺, (22), [–C_14_H_20_ClNO]	1.1	95.0296 [C_6_H_4_F]⁺, (0,1), [–CO]	4.7
			95.0296 [C_6_H_4_F]⁺, (0,1), [–C_15_H_20_ClNO_2_]	4.7	∖	∖

**TABLE 2:** List of MS*^n^* for aripiprazole, *m*/*z* 448.1565.

[MH]^+^	tr (min)	Δppm	MS^2^, molecular formula, (abundance %), [loss]	Δppm	MS^3^, molecular formula, (abundance %), [loss]	Δppm
448.1565 [C_23_H_28_Cl_2_N_3_O_2_]⁺	26.74	2.7	285.0930 [C_14_H_19_Cl_2_N_2_]⁺, (100) [–C_9_H_9_NO_2_]	3.6	216.0346 [C_10_H_12_Cl_2_N]⁺, (50) [–C_4_H_7_N]	2.2
					98.0961, [C_6_H_12_N_]_⁺, (100), [–C_8_H_7_Cl_2_N]	−3.3
			218.1180 [C_13_H_16_NO_2_]⁺, (9.3) [–C_10_H_12_Cl_2_N_2_]	2.0	176.0709 [C_10_H_10_NO_2_]⁺, (4.8) [–C_3_H_6_]	1.7
					164.0708 [C_9_H_10_NO_2_]⁺, (4.8) [–C_4_H_6_]	1.2
					148.0759 [C_9_H_10_NO]⁺, (0.7) [–C_4_H_6_O]	1.4
			176.0709 [C_10_H_10_NO_2_]⁺, (4.8) [–C_13_H_16_Cl_2_N_2_]	1.7	148.0759 [C_9_H_10_NO]⁺, (100) [–CO]	1.4
					120.0808, [C_8_H_10_N]⁺, (8) [–2CO]	0.2
					94.0651, [C_6_H_8_N]⁺, (1), [–C_2_H_2_O_2_]	−0.3
			148.0759 [C_9_H_10_NO]⁺, (0.7) [–C_14_H_18_Cl_2_N_2_O]	1.4	120.0808, [C_8_H_10_N]⁺, (100), [–CO]	0.2
					94.0651, [C_6_H_8_N]⁺, (4), [–C_3_H_2_O]	−0.3

The MS^2^ spectrum of haloperidol showed as most abundant fragment ions those at *m*/*z*: 358.1363 (C_21_H_22_ClFNO^+^, neutral loss of a molecule of water), 165.0712 (base peak, C_10_H_10_FO^+^, neutral loss of a molecule of 4‐(4‐chlorophenyl)piperidin‐4‐ol), and 123.0242 (C_7_H_4_FO^+^, neutral loss of a molecule of 4‐(4‐chlorophenyl)‐1‐propylpiperidin‐4‐ol).

For aripiprazole, the peculiar fragment ions were those with *m*/*z* 285.0930 (base peak, C_14_H_19_Cl_2_N_2_^+^, neutral loss of a molecule of 7‐hydroxy‐3,4‐dihydroquinolin‐2(1*H*)‐one) and 218.1180 (C_13_H_16_NO_2_^+^, neutral loss of a molecule of 1‐(2,3‐dichlorophenyl)piperazine).

Some of these fragments were also observed in the fragmentation pathways of the TPs deriving from haloperidol and aripiprazole, either with the same *m*/*z* or with a mass difference, for example, 15 Da, corresponding to the addition of one hydroxyl group in that portion of the molecule.

### 3.2 Transformation Products

**FIGURE 4:**
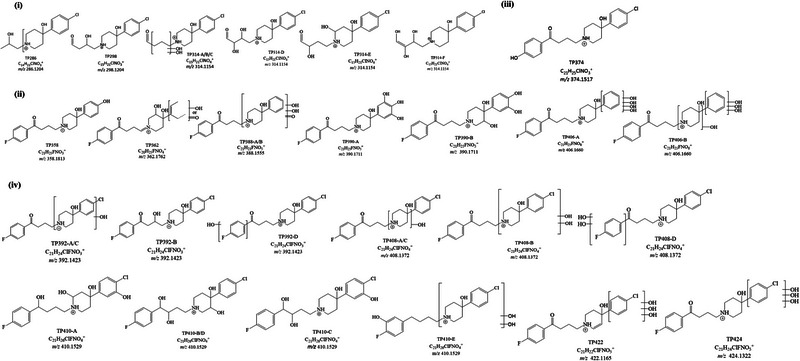
Hypothesized structures of transformation products (TPs_ha_) generated from haloperidol via TiO_2_‐mediated photocatalysis grouped for categories: (a) hydroxylated TPs with structure loss; (b) hydroxylated TPs with Cl loss; (c) hydroxylated TPs with fluorine loss; and (d) hydroxylated TPs.

All the samples subjected to irradiation were analyzed with the developed HPLC–HRMS method with a C18 column and ESI source in positive ionization mode. A total of 14 and 12 TPs were tentatively identified for haloperidol and aripiprazole, respectively, based on accurate mass measurements and fragmentation patterns (Figures 4 and 6). Most of these TPs were found to exist as multiple isomeric forms. The extracted ion chromatogram was obtained with a mass tolerance of 50 ppm, and compound selection was based on the corresponding MS*^n^* spectra.

Thanks to the use of the high resolving power instrumentation and MS^2^–MS^3^ experiments, it was possible to identify the accurate mass of the TPs and tentatively hypothesize the structural formula. The knowledge of fragmentation pathways and chemical structures of TPs is crucial to evaluate the efficacy of the photocatalysis process and to predict the possible environmental and toxicological implications of these compounds.

The MS*^n^* experiments were performed after 10 min of photocatalysis, since the TPs signals were more intense at that time as appearance/disappearance curves showed (Figures 8 and 9).

For simplicity, TPs and their kinetics have been grouped according to the category they belonged to: (a) hydroxylation with structure loss, (b) hydroxylation with Cl loss, (c) hydroxylation with F loss, and (d) hydroxylation, for haloperidol TPs; (a) modification or loss of the tetrahydroquinolone ring, (b) modification or loss of the dichlorobenzene ring, (c) modification of piperazine, and (d) generic structural changes, for aripiprazole TPs. They have been named with the pedis “ha” and “ar” if belonging to haloperidol and aripiprazole, respectively. The number refers to *m*/*z* and the letter A–B–C–n to the various isomers of the formed molecules. The retention times, fragmentation pathways, and tables summarizing the relative abundances, neutral losses, and chemical formulas of the TPs are provided in the Supporting Information section.

For haloperidol, all identified TPs, except for the two isoforms of TP_ha_314 (A and C), exhibited maximum intensities after 10 min of TiO_2_‐mediated photocatalysis, consistent with the 50% reduction of the parent compound at this time (Figures 1 and 8). Conversely, TP_ha_314 A and C reached their maximum abundance only after 60 min, indicating a slower formation pathway.

On the contrary, all aripiprazole TPs showed higher intensities after 10 min, in accordance with the faster disappearance kinetics of the drug (Figures 1 and 9). After 5 min, aripiprazole concentration was reduced to 10% of its initial value, demonstrating the high efficiency of TiO_2_ in degrading it.

**FIGURE 5:**
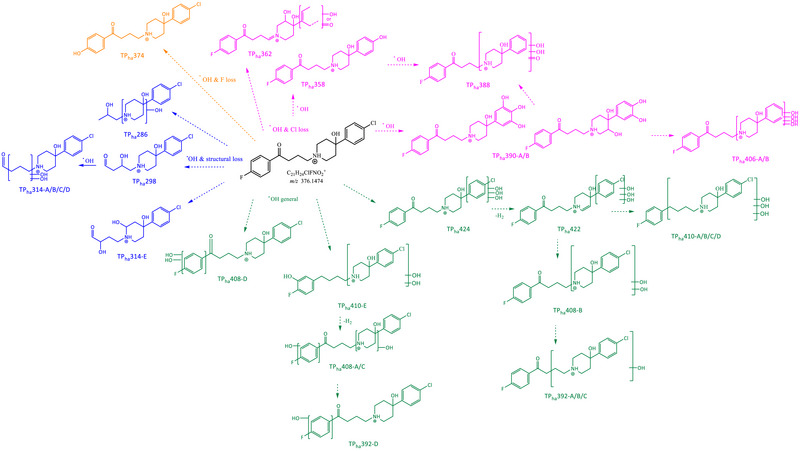
Proposed haloperidol transformation pathway indicating the identified TPs grouped for categories: (blue) hydroxylated TPs with structure loss; (pink) hydroxylated TPs with Cl loss; (orange) hydroxylated TPs with fluorine loss; and (green) hydroxylated TPs.

We tentatively proposed transformation pathways for haloperidol and aripiprazole (Figures 5 and 7) based on the findings reported below. The following paragraphs with the description of hypothesized chemical formula of TPs report only the fragment ions or neutral losses that were most relevant for the structural elucidation. A complete list of the observed fragments is provided in the Supporting Information section, as previously mentioned.

**FIGURE 6:**
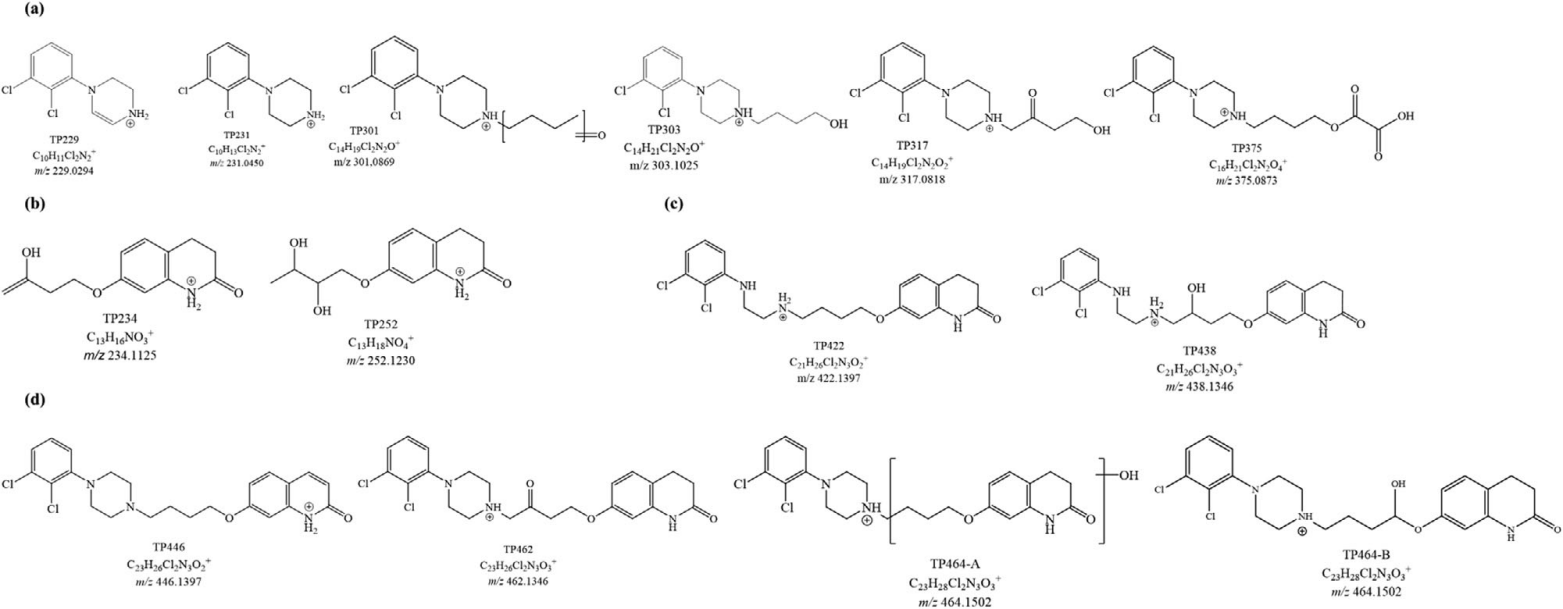
Hypothesized structures of transformation products (TPs_ar_) generated from aripiprazole via TiO_2_‐mediated photocatalysis grouped for categories: (a) TPs with modification or loss of tetrahydroquinolone ring; (b) TPs with modification or loss of dichlorobenzene ring; (c) TPs with modifications of piperazine ring; and (d) TPs with generic structural modifications.

**FIGURE 7:**
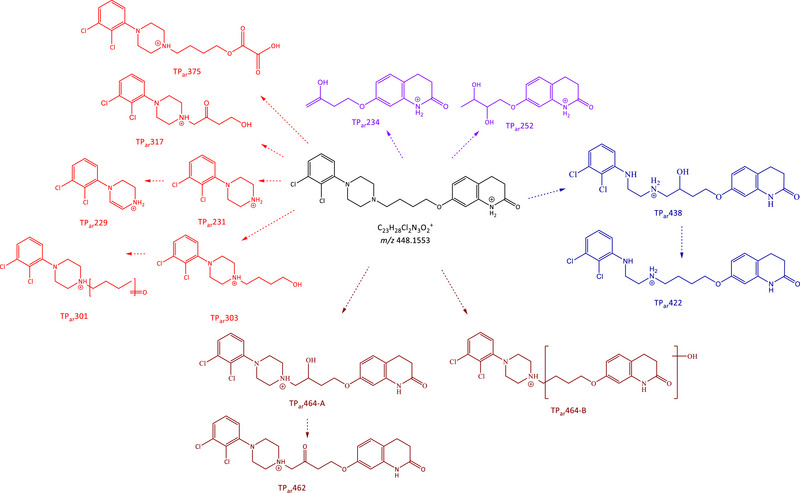
Proposed aripiprazole transformation pathway indicating the identified TPs grouped for categories: (red) TPs with modification or loss of tetrahydroquinolone ring; (violet) TPs with modification or loss of dichlorobenzene ring; (dark blue) TPs with modifications of piperazine ring; and (purple) TPs with generic structural modifications.

**FIGURE 8:**
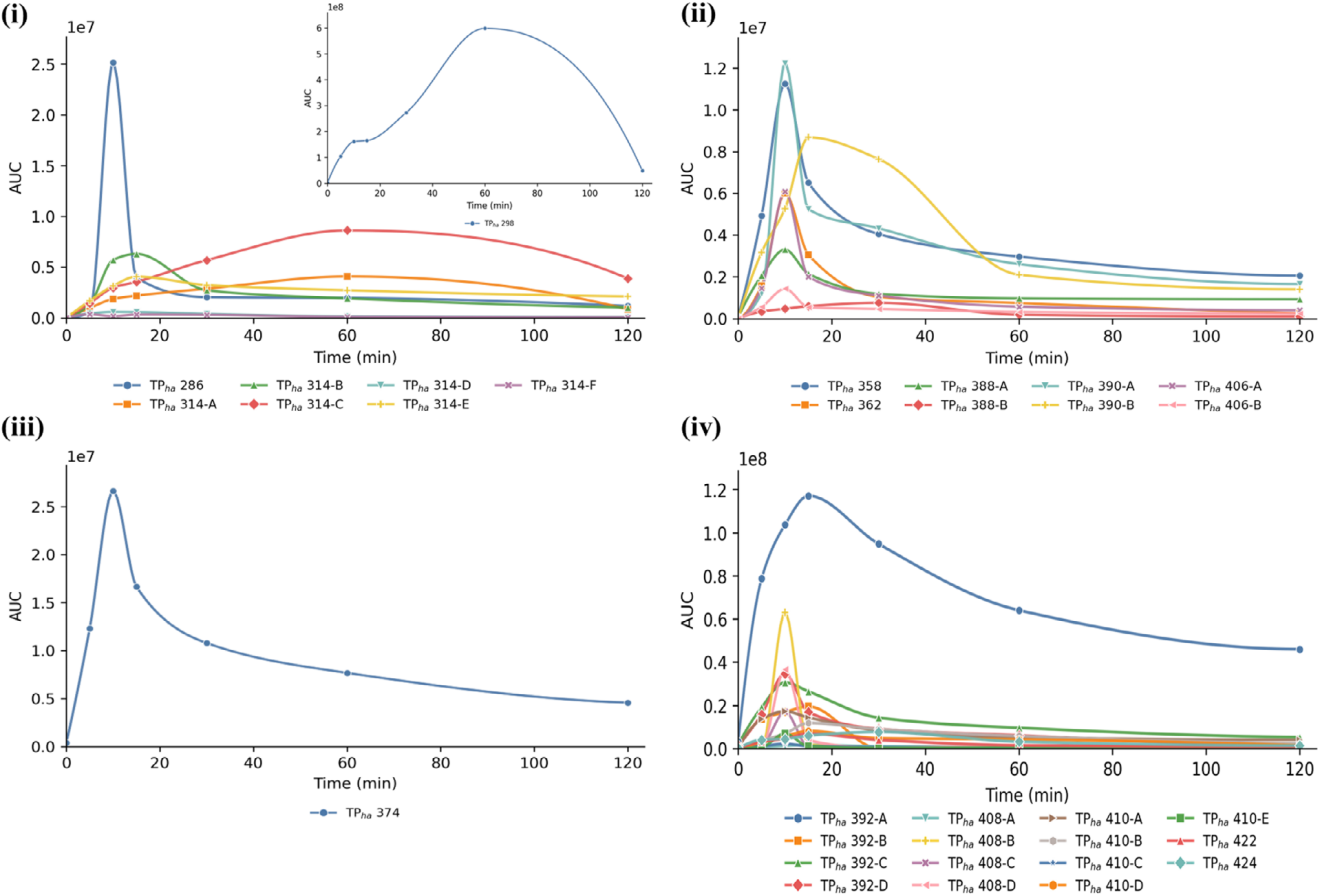
Appearance/Disappearance curves of haloperidol TPs originated after heterogeneous photocatalysis mediated by TiO_2_ grouped for categories: (a) hydroxylated TPs with structure loss; (b) hydroxylated TPs with Cl loss; (c) hydroxylated TPs with fluorine loss; and (d) hydroxylated TPs.

**FIGURE 9:**
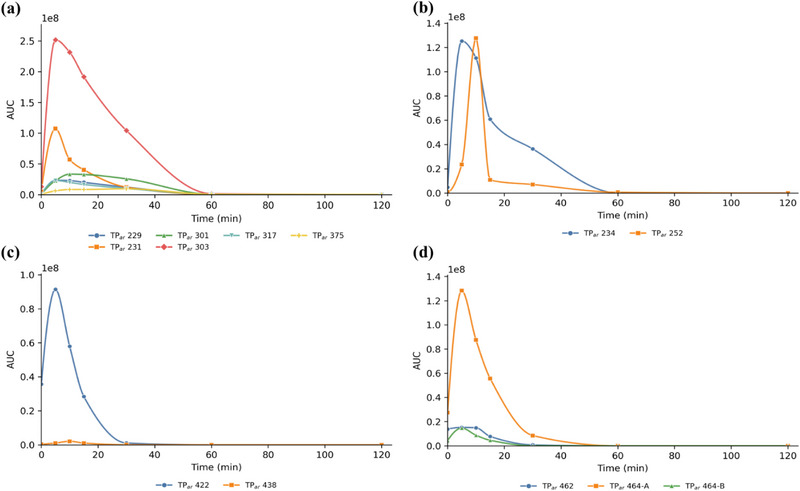
Appearance/Disappearance curves of aripiprazole TPs originated after heterogeneous photocatalysis mediated by TiO_2_ grouped for categories: (a) TPs with modification or loss of tetrahydroquinolone ring; (b) TPs with modification or loss of dichlorobenzene ring; (c) TPs with modifications of piperazine ring; and (d) TPs with generic structural modifications.

#### 3.2.1 Haloperidol Hydroxylated TPs With Structure Loss

Three species were detected at *m*/*z* 286.1186, 298.1200, and 314.1132. These TPs were characterized by the absence of the typical haloperidol product ions containing the fluorine atom (*m*/*z* 206, 95, 194, 165, and 123), confirming that hydroxylation and other structural modifications occurred on the chlorophenyl‐hydroxypiperidine moiety.

**TP_ha_286** ([C_14_H_21_ClNO_3_]^+^, Figures S1 and S2, Table S2) was generated through the loss of 1‐(4‐fluorophenyl)ethan‐1‐one, accompanied by two hydroxylations. In the MS^2^ spectrum, two main product ions were identified: *m*/*z* 268.1086, corresponding to the loss of H_2_O, and *m*/*z* 224.0825, resulting from a concerted neutral loss of acetaldehyde and water. The elimination of acetaldehyde indicated that one hydroxylation occurred on the aliphatic side chain, whereas the second was located on the hydroxypiperidine ring. This structural hypothesis was strongly supported by MS^3^ analysis.

**TP_ha_298** ([C_15_H_21_ClNO_3_]⁺, Figures S3 and S4, Table S3) retained the product ion at *m*/*z* 194.0731 in its MS^2^ spectrum, indicating that the chlorophenyl‐hydroxypiperidine moiety remained unmodified. Additional MS^2^ and particularly MS^3^ product ions supported the hypothesis that one hydroxylation and one oxidation occurred on the aliphatic side chain. The MS^3^ ion at *m*/*z* 234.1035 arose from a concerted loss of acetaldehyde and water, whereas the ion at *m*/*z* 165.0458 confirmed the presence of a chlorine atom, as evidenced by its characteristic isotopic pattern.

**TP_ha_314** ([C_15_H_21_ClNO_4_]^+^, Figures S5–S9, Table S4) showed six distinct isomers. Isomers **TP_ha_314‐A/B** and **C** were not distinguished only on the basis of the acquired information. All of them showed as main product ions *m*/*z* 296 (loss of water) and 210 (concerted loss of water and C_4_H_6_O_2_). MS^3^ product ion at *m*/*z* 197.0722, assigned to 3‐(4‐chlorophenyl)‐3‐hydroxypentano, was observed for isomers **TP_ha_314‐B/C**. This ion resulted from the loss of C_4_H_5_NO_2_, explained by charge migration and hydrogen scrambling [40, 41, 42]. The proposed structure of TP_ha_314‐A/B/C involved both hydroxyl groups on the aliphatic chain. **TP_ha_314‐D** was the only isomer showing, in its MS^2^ spectrum, a concerted loss of carbon dioxide and water, followed by rearrangement, suggesting the formation of a diol on the aliphatic chain. **TP_ha_314‐E** exhibited an intense MS^2^ product ion at *m*/*z* 116.0700 (relative abundance 61%), consistent with 4,6‐dihydroxy‐2,3,4,5‐tetrahydropyridine, indicating hydroxylation on the hydroxypiperidine ring. The subsequent detection of *m*/*z* 98.0600 in MS^3^ fragmentation provided strong support for this structural assignment. Finally, on the basis of the presence in the MS^2^ spectrum of product ions at *m*/*z* 224.0825 (C_12_H_15_ClNO^+^), 210.0670 (C_11_H_13_ClNO^+^), and 165.0459 (C_10_H_10_Cl^+^), the structure of **TP_ha_314‐F** was tentatively assigned as bearing three hydroxyl groups and an unsaturation, all localized on the aliphatic side chain.

#### 3.2.2 Haloperidol Hydroxylated TPs With Cl Loss

The most frequent reactions found were hydroxylation and Cl loss. Six species were revealed at *m*/*z* 358.1795, 362.1746, 378.1696, 388.1544, 390.1700, and 406.1638. For all of them, MS^2^ fragmentation experiments revealed product ions at *m*/*z* 165 and 123, which are shared with haloperidol, confirming the presence of at least the fluorobenzaldehyde moiety in the molecular structure. No isotopic distribution of chlorine atom in MS and MS*^n^* spectra was observed for these TPs.

**TP_ha_358** ([C_21_H_25_FNO_3_]⁺, Figure S10, Table S5) showed the common substitution of the chlorine atom with a hydroxyl group [43].

The fragmentation pattern of **TP_ha_362** ([C_20_H_25_FNO_4_]^+^, Figures S11 and S12, Table S6) strongly supported the formation of a quaternary amine, with one of the two hydroxylations occurring on the hydroxypiperidine ring. The second transformation could involve either hydroxylation or oxidation following the substitution of the chlorine atom with an open benzene ring. The presence of product ions at *m*/*z* 165 and 123 ruled out a double bond in the fluorophenyl‐butanone portion, whereas the ions at *m*/*z* 244 and 218 supported this hypothesis.

**TP_ha_388** ([C_21_H_23_FNO_5_]^+^, Figures S13 and S14, Table S7) had two isomers for which it was not possible to distinguish a different molecular structure hypothesis, as the fragmentation pathway did not allow it. With certainty, the two hydroxyl groups and the oxidation occurred on the hydroxy‐phenylpiperidine moiety because of the presence in the MS^2^ spectra of *m*/*z* 224.0917. It was not possible to indicate the exact position of the substitutions due to the lack of information deriving from MS*^n^* experiments.

The fragmentation pathway of **TP_ha_390** ([C_21_H_25_FNO_5_]⁺, Figures S15–S17, Table S8) showed some peculiar neutral loss that supported the proposed molecular structure. First, for isomer **TP_ha_390‐A**, MS^2^ product ion *m*/*z* 264.1390 with chemical formula C_15_H_19_FNO_2_^+^ derived from the loss of a molecule of benzene‐1,2,3‐triol suggesting the occurrence of three hydroxylations on benzene ring (the exact position was not known; it was only a speculation). In addition, the molecule did not fragment by losing two water molecules, further supporting the previous findings. Conversely, isomer **TP_ha_390‐B** exhibited the loss of two water molecules (product ion *m*/*z* 354.1500) and shared with isomer A the product ion *m*/*z* 226.1074, indicating a double hydroxylation on the benzene ring along with a single hydroxylation on the hydroxypiperidine ring.

The same considerations were done for **TP_ha_406** ([C_21_H_25_FNO_6_]⁺, Figures S18–S20, Table S9) with four hydroxylations. For isomer **TP_ha_406‐A**, hydroxylation occurred on benzene ring, whereas for isomer **TP_ha_406‐B,** one of the four substitutions took place on hydroxypiperidine ring.

#### 3.2.3 Haloperidol Hydroxylated TPs With F Loss

One species, **TP_ha_374**, was detected at *m*/*z* 374.1503 ([C_21_H_25_ClNO_3_]⁺, Figures S21 and S22, Table S10), formed by the loss of a fluorine atom and the subsequent substitution with a hydroxyl group. All MS^2^ and MS^3^ experiments supported this hypothesis, as the product ions showed a mass difference of 2 Da (–F +OH) compared with those of haloperidol.

#### 3.2.4 Haloperidol Hydroxylated TPs

Five TPs were detected at *m*/*z* 392.1416, 408.1358, 410.1515, 422.1156, and 424.1304. These TPs were particularly distinctive due to the presence of hydroxyl or oxo groups introduced at various positions within the parent structure. Elucidation of their structural formulas relied heavily on the interpretation of fragmentation pathways.

Four isomeric forms of **TP_ha_392** ([C_21_H_24_ClFNO_3_]⁺, Figures S23–S26, Table S11) were identified, eluting at approximately 20 min. The first and third isomers, **TP_ha_392‐A** and **TP_ha_392‐C** (based on elution order), yielded product ions at *m*/*z* 194.0971, 165, and 123, consistent with those observed for haloperidol. These fragments suggested that hydroxylation occurred on the 4‐(4‐chlorophenyl)piperidin‐4‐ol moiety. However, the exact position of the hydroxyl group could not be determined based solely on MS*^n^* data.

In contrast, although the second peak appeared chromatographically only as a minor shoulder of the main peak at Rt = 18.45 min, corresponding to isomer TP_ha_392‐A (Figure S26), **TP_ha_392‐B** exhibited extensive fragmentation. The generation of multiple MS*^n^* product ions suggested that TP392‐B possesses a distinct chemical structure from TP_ha_392‐A. MS^3^ fragments at *m*/*z* 208, 125, and 194.0731 indicated that hydroxylation did not involve the 4‐(4‐chlorophenyl)piperidin‐4‐ol ring but instead occurred on the butyl side chain. This hypothesis was further supported by the product ion at *m*/*z* 163.0554, which differs by 2 Da from the *m*/*z* 165.0710 fragment characteristic of haloperidol, consistent with hydroxylation of the aliphatic chain. The final isomer, **TP_ha_392‐D**, exhibited a predominant product ion at *m*/*z* 181.0659 (100% relative abundance), corresponding to 1‐(4‐fluoro‐*X*‐hydroxyphenyl)butan‐1‐one, where *X* denotes a potential hydroxylation site on the fluorobenzene ring. The exact position of the hydroxyl group could not be unambiguously determined from MS*^n^* experiments. This hypothesis was supported by MS^3^ product ions at *m*/*z* 139, consistent with 4‐fluoro‐*X*‐hydroxybenzaldehyde, and at *m*/*z* 125, attributed to *X*‐fluoro‐5‐methylphenol.

A second hydroxylation on the haloperidol backbone characterized the four isomer forms of **TP_ha_408** ([C_21_H_24_ClFNO_4_]⁺, Figures S27–S30, Table S12). Similarly to TP_ha_392‐D, which exhibited a predominant product ion at *m*/*z* 181.0659, the first and third isomers of TP_ha_408, **TP_ha_408‐A** and **TP_ha_408‐C** (based on elution order), also showed the MS^2^ product ions at *m*/*z* 181 and 139.0183. The presence of these ions further supports the hypothesis that hydroxylation occurred on the fluorobenzene ring in both cases. The second hydroxylation is proposed to have occurred on the piperidin‐4‐ol ring, based on the detection of product ions at *m*/*z* 210, 240, and 372. In particular, this last ion *m*/*z* 372 also confirmed that no modifications took place on the chlorobenzene moiety, as it resulted from the neutral loss of two water molecules from the parent compound. In contrast, the isomer **TP_ha_408‐B** exhibited two hydroxylations on the chlorophenyl‐hydroxypiperidine moiety. The presence of product ions at *m*/*z* 244 and 226 in the MS^2^ spectrum supported this hypothesis, although the exact position of the substituents remains uncertain. Additionally, the ions at *m*/*z* 165, 123, and 194 further corroborated this conclusion. Finally, in **TP_ha_408‐D**, a double hydroxylation occurred on the fluorobenzene ring. All MS*^n^* product ions strongly supported this hypothesis, as their *m*/*z* values were 32 Da higher compared to those derived from haloperidol (*m*/*z* 155 vs. 123; *m*/*z* 197 vs. 165; *m*/*z* 224 vs. 194).

**TP_ha_410** ([C_21_H_26_ClFNO_4_]⁺, Figures S31–S35, Table S13) characterized by two hydroxylations and one reduction, exhibited five isomeric forms. Similarly to isomer TP_ha_410‐C, isomer **TP_ha_410‐A** exhibited a concerted neutral loss of water and hydrogen chloride, yielding the product ion at *m*/*z* 356.1639, potentially indicating a hydroxylation on the chlorobenzene ring. However, unlike isomer C, the presence of the MS^2^ product ion at *m*/*z* 194.0968 (C_11_H_13_FNO^+^) suggested that the second hydroxylation occurred on the piperidine ring. **TP_ha_410‐B/D** displayed both *m*/*z* 165 and 181 as MS^2^ product ions. However, the latter did not generate *m*/*z* 139 upon further fragmentation, suggesting the presence of a hydroxyl group on the butyl side chain rather than on the fluorobenzene ring. Additional MS*^n^* product ions supported the localization of the second hydroxylation on the piperidine ring and indicated that the ketone group was reduced to a secondary alcohol. **TP_ha_410‐C** exhibited a product ion at *m*/*z* 356.1639 (C_21_H_23_FNO_3_) with 34% relative abundance, resulting from the concerted neutral loss of one water molecule and one hydrogen chloride molecule. The only plausible explanation for the elimination of HCl involves a hydroxylation on the chlorobenzene ring, potentially accompanied by the formation of a cyclic epoxide. Moreover, fluorine was eliminated as hydroxy fluoride from **TP_ha_410‐E**, and in this case, one of the three hydroxylations occurred on fluorobenzene ring with the subsequent formation of cyclic epoxide. The other two hydroxylations took place on the chlorophenyl‐piperidin ring.

**TP_ha_422** ([C_21_H_22_ClFNO_5_]⁺, Figures S36 and S37, Table S14) was formed through triple hydroxylation on the chlorobenzene ring and dehydrogenation of the hydroxypiperidine ring. This hypothesis was supported by the absence of a second neutral loss of water and the presence of MS^2^ product ions at *m*/*z* 272, 254, 258, and 240. The same held true for **TP_ha_424** ([C_21_H_24_ClFNO_5_]⁺, Figures S38 and S39, Table S15), which originated from the previous one following the hydrogenation of the hydroxypiperidine ring.

#### 3.2.5 Aripiprazole TPs With Modification or Loss of the Tetrahydroquinolone Ring

Seven TPs resulting from the heterogeneous photocatalysis of aripiprazole were detected at *m*/*z* 229.0284, 231.0444, 301.0864, 303.1018, 317.0811, 319.0960, and 375.0865.

The isotopic pattern of **TP_ar_229** ([C_10_H_11_Cl_2_N]⁺, Figures S40 and S41, Table S16) clearly indicated the presence of two chlorine atoms in the structure. Moreover, no typical MS*^n^* product ions associated with the dihydroquinolin‐2(1*H*)‐one moiety were detected, supporting the assignment of TP_ar_229 as 4‐(2,3‐dichlorophenyl)‐1,2,3,4‐tetrahydropyrazine. **TP_ar_231** ([C_10_H_13_Cl_2_N_2_]⁺, Figures S42 and S43, Table S17), arising from dehydrogenation of the piperazine ring, shared with TP229 the fragment ion at *m*/*z* 200. Notably, a radical loss of chlorine generated a product ion at *m*/*z* 153.0340, a fragmentation pathway also described for other chlorinated compounds undergoing TiO_2_‐mediated photocatalysis [43]. Similarly, **TP_ar_301** ([C_14_H_19_Cl_2_N_2_O]⁺, Figures S44 and S45, Table S18) exhibited a neutral loss of a chlorine radical, yielding *m*/*z* 153.0340. The molecular mass of the precursor ion, along with the presence of product ions at *m*/*z* 231 and 188 in the MS^2^ spectra, supported the occurrence of oxidation on the aliphatic chain. Following this transformation route, we identified **TP_ar_303** ([C_14_H_21_Cl_2_N_2_O]⁺, Figures S46 and Table S19), with an exact mass 2 Da higher than the previous TP301. In this case, the ketone (or aldehyde) group on the aliphatic chain was reduced to a hydroxyl group. The proposed structural formula suggests that it could lose a water molecule, leading to the formation of an unsaturation on the aliphatic chain (*m*/*z* 285.0920), and subsequently undergo fragmentation similar to TP_ar_301 and TP_ar_229. As observed for TP_ar_303, the product ion *m*/*z* 153.0340 was generated following the loss of a radical chlorine atom.

**TP_ar_317** ([C_14_H_19_Cl_2_N_2_O_2_]⁺, Figures S47 and S48, Table S20) was structurally related to the previous ones, featuring an additional hydroxylation. After analyzing its MS*^n^* product ions, we hypothesized two hydroxylations (one followed by an oxidation) on the aliphatic side chain. We observed a neutral loss of two water molecules and the presence of key product ions at *m*/*z* 229, 202, 231, and 188, confirming that no modifications occurred on the 1‐(2,3‐dichlorophenyl)piperazine moiety.

The isotopic distribution and chemical formula of **TP_ar_375** ([C_16_H_21_Cl_2_N_2_O_4_]⁺, Figures S49 and S50, Table S21) suggested the cleavage of the 3,4‐dihydroquinolin‐2(1*H*)‐one ring, followed by three hydroxylation events on the remaining carbon backbone. Fragmentation was limited, and no additional information was obtained from the MS*^n^* spectra.

#### 3.2.6 Aripiprazole TPs With Modification or Loss of the Dichlorobenzene Ring

Two species were detected at *m*/*z* 234.1116 (**TP_ar_234**, [C_13_H_16_NO_3_]⁺, Figures S51 and S52, Table S22) and 252.1220 (**TP_ar_252**, [C_13_H_18_NO_4_]⁺, Figures S53 and S54, Table S23). No isotopic distribution of the chlorine atom was observed for these molecules, confirming the modification of the 3,4‐dihydroquinolin‐2(1*H*)‐one moiety. For these TPs, MS*^n^* experiments provided limited information, revealing only that the modification occurred on the aliphatic side chain (the indicated position is a speculation). The presence of product ions at *m*/*z* 164 and 122 supported this hypothesis. In TP_ar_234, a single hydroxylation occurred, whereas TP_ar_252 underwent two hydroxylations.

#### 3.2.7 Aripiprazole TPs With Modification of the Piperazine Ring

**TP_ar_422** ([C_21_H_26_Cl_2_N_3_O_2_]⁺, Figures S55 and S56, Table S24) and **TP_ar_438** ([C_21_H_26_Cl_2_N_3_O_3_]⁺, Figures S57 and S58, Table S25) with exact mass *m*/*z* 422.1400 and *m*/*z* 438.1347, respectively, were identified as TPs resulting from piperazine ring cleavage. These two TPs shared the MS^2^ product ion at *m*/*z* 259.0763, indicating the retention of both chlorine atoms in their structures. In the case of TP_ar_438, the loss of a water molecule in MS^2^ suggested that hydroxylation occurred on the aliphatic chain; however, a possible alternative location could be the piperidin‐2‐one ring. Nonetheless, the available data did not allow for unambiguous determination of the exact hydroxylation site.

#### 3.2.8 Aripiprazole TPs With Generic Structure Modification

For the following TPs, it was not possible to correlate the proposed structural formulas with a specific or characteristic modification of aripiprazole. These compounds appeared to result from multiple transformations involving various regions of the parent molecule.

A hydroxylation on the butyl side chain, followed by a reduction, was observed in the TP **TP_ar_462** ([C_23_H_26_Cl_2_N_3_O_3_]⁺, Figures S59 and S60, Table S26). The neutral loss of carbon monoxide was consistent with the cleavage of the piperidin‐2‐one ring and was followed in MS^3^ by the elimination of a C_10_H_13_NO fragment, resulting in the formation of the product ion at *m*/*z* 271.0392.

The final TP identified was **TP_ar_464** ([C_23_H_28_Cl_2_N_3_O_3_]⁺, Figures S61–S63, Table S27), which was detected in two isomeric forms. MS*^n^* fragmentation experiments for **TP_ar_464‐A** provided limited structural information due to poor spectral quality. However, the hydroxylation was tentatively assigned to the 7‐butoxy‐3,4‐dihydroquinolin‐2(1*H*)‐one moiety. In contrast, **TP_ar_464‐B** displayed rich fragmentation patterns in both MS^2^ and MS^3^ spectra. The simultaneous detection of product ions at *m*/*z* 285 and 243 (containing chlorine atoms), along with ions at *m*/*z* 234, 216, 176, and 164, strongly suggested that hydroxylation occurred on the aliphatic butyl chain. The observation of a product ion at *m*/*z* 303.1025 further supported the hypothesis that the hydroxyl group was located on the carbon adjacent to the ether oxygen.

### 3.3 Semi‐Quantitative Analysis in Real River Samples

The developed HPLC–HRMS method was subsequently applied to real water samples collected from three distinct locations: two in the city center of Turin (Italy), corresponding to the Po and Sangone Rivers, and one in the province of Turin at a mountain site in Forno di Coazze (1000 meters above sea level), also part of the Sangone River system.

Prior to the quantitation of haloperidol, aripiprazole, and their TPs, the limits of detection (LOD) and quantitation (LOQ) were determined. For haloperidol, the LOD and LOQ were 0.40 and 1 ng L^−1^, respectively, whereas for aripiprazole, the LOD was 4.5 ng L^−1^ and the LOQ 10 ng L^−1^. In addition, SPE recovery was evaluated, yielding 77% for haloperidol and 45% for aripiprazole. The molecules were semi‐quantified using an external calibration curve of haloperidol and aripiprazole as reported in Section 2.3. As previously described, all samples were processed using an SPE protocol, and a semi‐quantitative analysis was carried out over 3 months (June, July, and August 2024) to estimate the concentrations of both parent compounds and their TPs. The results are summarized in Table 3. Although analytical standards for haloperidol and aripiprazole are commercially available, the concentrations of their TPs were estimated by assuming similar ionization behavior to that of the parent compounds. This approach has evident limitations [44, 45]; however, to our knowledge, it represents one of the feasible options.

**TABLE 3:** Concentrations (ng/L) of haloperidol, aripiprazole, and their transformation products (TPs) detected in environmental river water samples.

	Concentrations (ng/L)								
	June 2024			July 2024			August 2024		
	Po River, Torino	Sangone River, Moncalieri	Sangone River, Forno di Coazze	Po River, Torino	Sangone River, Moncalieri	Sangone River, Forno di Coazze	Po River, Torino	Sangone River, Moncalieri	Sangone River, Forno di Coazze
Haloperidol	n.d.	n.d.	n.d.	n.d.	**3**	**27**	25	**7**	**8**
Aripiprazole	n.d.	n.d.	n.d.	n.d.	**67**	**29**	**28**	n.d.	**34**
TP314‐F	n.d.	n.d.	n.d.	n.d.	n.d.	n.d.	**0.16**	n.d.	n.d.
TP388‐B	n.d.	n.d.	n.d.	n.d.	n.d.	n.d.	**0.25**	n.d.	n.d.
TP392‐D	n.d.	n.d.	n.d.	n.d.	n.d.	n.d.	**0.47**	n.d.	n.d.

*Note*: n.d., not detectable (<LOQ).

Haloperidol was detected in all river samples, with the highest concentration of 27 ng L^−1^ observed in July in the Sangone River at Forno di Coazze. Aripiprazole was also found in all samples, reaching a maximum concentration of 67 ng L^−1^ in the Sangone River at Moncalieri. In August 2024, haloperidol and aripiprazole were both detected and quantified in the Po River, with concentrations of 25 and 28 ng L^−1^, respectively. Additionally, several TPs were identified and semi‐quantified in the same river, with concentrations of 0.16, 0.25, and 0.47 ng L^−1^ for TP_ha_314‐F, TP_ha_388‐B, and TP_ha_392‐D, respectively. To our knowledge, this is the first report of TPs derived from haloperidol detected in environmental samples. As highlighted in previous studies on other pharmaceuticals, such as antidepressants, the occurrence of TPs may be harmful to living organisms and pose a significant risk to the aquatic environment [46].

Previous research studies have also reported the presence of antipsychotic drugs in environmental waters worldwide, often using HPLC–MS(MS) analytical techniques and finding comparable concentration ranges.

Silveira et al., Caldas et al., and Reichert et al. [47, 48, 49] focused on the Brazilian territory, reporting haloperidol in surface waters in Rio Grande at concentrations of 0.1, 0.04, and 1.43 µg L^−1^, respectively. In Oceania, Dehm et al. reported haloperidol in surface waters along the southern coast of Viti Levu in Fiji, with concentrations ranging between 0.47 and 34 ng L^−1^ [50]. In Asia, Goswami et al. detected haloperidol in WWTP influents in Sri Lanka at concentrations between 2.6 and 58.8 ng L^−1^ [51]. In Europe, Grabic et al. measured haloperidol in Swedish WWTP effluents, reporting concentrations between 1.0 and 4.7 ng L^−1^ [52]. In Greece, Christophoridis et al. observed an average concentration of 20.9 ng L^−1^ in WWTP influents in Thessaloniki [53], whereas in Ioannina, Konstas et al. found 42.0 ng L^−1^ in WWTP effluent [54].

Aripiprazole has also been detected in WWTP influents, effluents, and surface water samples. In the United States, Croft [55] and Subedi [56] reported concentrations of 5.69 ng L^−1^ in WWTP influent (Kentucky) and 22.4 ng L^−1^ in WWTP effluent (Albany, NY, USA), respectively. Skees et al. also detected aripiprazole in surface waters of the Bee Creek River (Kentucky) with concentrations ranging from 5.1 to 8.3 ng L^−1^ [57]. In India, Subedi et al. found aripiprazole in WWTP influents at concentrations between 4.2 and 14 ng L^−1^ [58].

Our sampling campaign, carried out during the summer months (wet season), revealed an increase in haloperidol and aripiprazole concentrations from June to August, in line with rainfall patterns (14 rainy days in June vs. 6 in August). Drug levels ranged from not detectable in June to an average of 23.8 ng L^−1^ in August. Although the number of surface water samples was limited, some considerations can be drawn. The high number of rainy days in June likely increased the volume of river water, leading to undetectable concentrations due to dilution [59, 60, 61]. In addition, monthly drug consumption could influence their occurrence in surface waters, as previously reported for other pharmaceuticals by Lindholm‐Lehto et al. [62].

Quite surprisingly, in July and August, we observed elevated concentrations of drugs in the Sangone River at a remote site (Forno di Coazze, 45.03164° N, 7.23490° E; July: haloperidol 27 ng L^−1^, aripiprazole 29 ng L^−1^; August: haloperidol 8 ng L^−1^, aripiprazole 34 ng L^−1^). This area is highly frequented in summer for trekking and hiking, and the large number of visitors, combined with the limited dilution capacity of the watercourse, more a creek than a river, may explain these unexpectedly high concentrations [63, 64]. The results obtained from our study, together with those in literature, confirm that haloperidol, aripiprazole, and TPs are relevant emerging contaminants in surface waters, warranting increased attention due to their potential ecotoxicological implications. However, it should be noted that the majority of sewage discharges flow into WWTPs, suggesting that the concentrations detected in surface waters are likely underestimated with respect to their actual environmental impact.

### 3.4 Results of Toxicity Test

**FIGURE 10:**
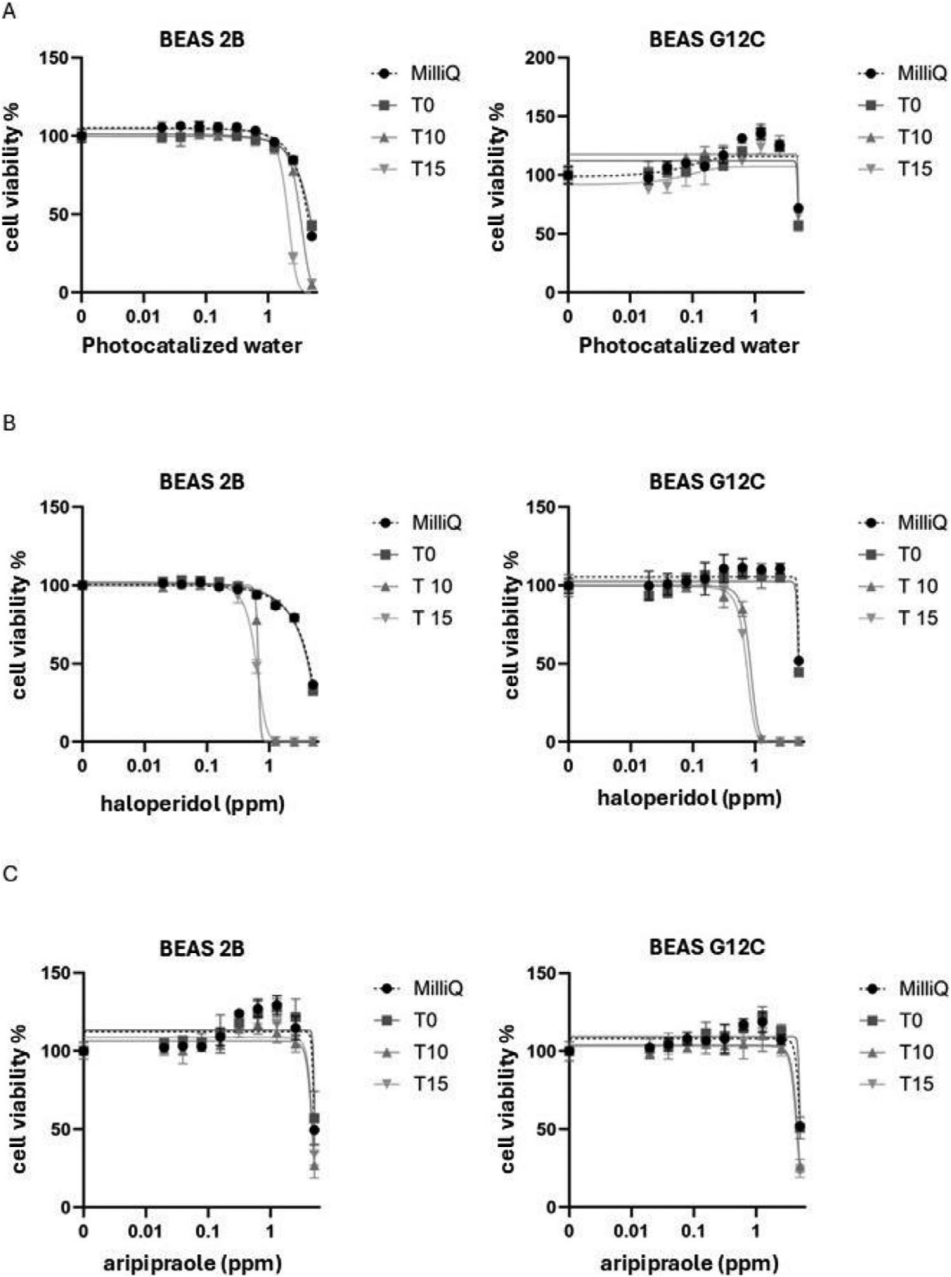
Evaluation of cell viability following treatment with (a) photocatalyzed water (best of two biological replicates); (b) haloperidol TPs (best of three biological replicates); (c) aripiprazole TPs (best of two biological replicates).

To evaluate the potential harmful effects of the identified TPs on human health, cytotoxicity assays were performed using both non‐tumorigenic (BEAS‐2B) and cancerous (BEAS‐G12C) human pulmonary epithelial cells. The BEAS‐G12C cell model was generated via stable retroviral infection. As described in Section 2.7, haloperidol and aripiprazole were photocatalyzed at several time points (0–10–15 min), and after 0.2 µm filtration, this mixture of formed TPs and parent compounds was used. Cell lines were treated with serial dilution of different photocatalyzed compounds to obtain a dose‐dependent curve (Figure 10). After 72 h of exposure, cell viability was assessed using a standard vitality assay (Figure 10). Milli‐Q water and TiO_2_‐photocatalyzed water were included as controls to exclude potential toxic effects due to osmotic stress or residual titanium dioxide.

As shown in Figure 10a, TiO_2_‐treated water significantly reduced cell viability in BEAS‐2B cells at higher concentrations (<10% viability), whereas BEAS‐G12C cells displayed greater resistance. Interestingly, haloperidol TPs collected at 10 and 15 min of photocatalysis were highly toxic to both cell lines, with significant cytotoxic effects observed even below 1 ppm (Figure 10b). In contrast, treatment with aripiprazole TPs showed no significant toxicity in either cell line (Figure 10c).

Overall, these findings indicate an acute cytotoxic effect of haloperidol TPs at elevated concentrations, with no substantial difference between normal and cancer‐derived pulmonary models. However, further studies are required to investigate the effects of long‐term exposure, as well as potential impacts in other human and animal models, to better understand the environmental and health risks associated with these TPs. Given the increasing consumption of psychotropic drugs [65] and the fact that haloperidol concentrations in aquatic environments have already exceeded the critical threshold proposed by Fick et al. [66], these compounds may represent a growing concern for public health.

## 4 Conclusions

In this study, we investigated the degradation pathways of two widely used antipsychotic drugs, haloperidol and aripiprazole, through heterogeneous photocatalysis mediated by TiO_2_ in ultrapure water. This approach not only enabled a significant reduction in the concentration of the parent compounds but also allowed for an in‐depth exploration of their TPs, including structural elucidation and fragmentation pathways.

The application of HPLC–HRMS was instrumental in the identification and characterization of the TPs. Tailored MS*^n^* experiments enabled the reconstruction of fragmentation patterns and supported the proposed structural formulas, even distinguishing among positional isomers.

For haloperidol, 14 TPs (32 including isomers) were identified and classified into four main structural categories: structural loss, hydroxylation with chlorine loss, hydroxylation with fluorine loss, and pure hydroxylation. Aripiprazole yielded 12 TPs (13 with isomers), categorized as modifications or losses of the tetrahydroquinoline moiety, changes to the dichlorobenzene ring, and other generic structural alterations.

The degradation kinetics of both drugs and the formation/degradation trends of their TPs during TiO_2_‐mediated photocatalysis were also evaluated. After 120 min of treatment, haloperidol showed a degradation rate of 93.2%, whereas aripiprazole was almost completely removed from the solution.

Subsequently, the developed HPLC–HRMS method was applied to real river surface water samples collected in Northern Italy. An SPE procedure was used for environmental analysis. Sampling was conducted in June, July, and August 2024. Haloperidol was detected at its highest concentration (27 ng L^−1^) in July in the Sangone River near Forno di Coazze (Turin), whereas aripiprazole peaked at 67 ng L^−1^ in the Sangone River at Moncalieri. Among the TPs, three haloperidol TPs were detected in the Po River in August. In the future, a larger number of surface water samples could be analyzed to better correlate these findings with continuous monitoring of antipsychotic drugs.

Finally, the cytotoxicity of the parent compounds and their TPs (at t0′, t10′, and t15′) was assessed on both normal (BEAS‐2B) and cancerous (BEAS G12C) human pulmonary epithelial cell lines. Although the parent drugs did not display toxicity in this cellular model, a marked cytotoxic effect was observed for haloperidol TPs in both cell lines, indicating their potential harmfulness.

These results underscore the importance of investigating not only the fate of pharmaceuticals in aquatic environments but also the biological activity of their TPs. The findings presented here highlight the relevance of haloperidol and aripiprazole as emerging contaminants and raise concerns about the potential ecological and health risks posed by their TPs.

## Conflicts of Interest

The authors declare no conflicts of interest.

## Supporting information



Supporting File: jssc70299‐sup‐0001‐SuppMat.docx

## Data Availability

The data used to support the findings of this study are available from the corresponding author upon request.
