# Supplementary material for: Development of an HPLC–HRMS Method for the Identification and Semi‐Quantitation in River Water Samples of the Transformation Products Originated From Heterogeneous Photocatalysis of Antipsychotic Drugs
Source: J Sep Sci. 2025 Oct 14;48(10):e70299. doi: 10.1002/jssc.70299 (PMC12520203; doi:10.1002/jssc.70299)
Supplement: Supplementary file 1 — Supporting File: jssc70299‐sup‐0001‐SuppMat.docx [file JSSC-48-e70299-s001.docx]

**Development of an HPLC-HRMS method for the determination of TiO_2_-mediated photocatalysis products of the antipsychotic drugs haloperidol and aripiprazole and research in river waters**

Elena De Rosa^1^, Serena Arpaia^1^, Sandra Vietti Michelina^1^, Francesco Oliva^2^, Claudio Medana^1^, Paola Calza^3^, Federica Dal Bello^1#^

^1^Department of Molecular Biotechnology and Health Science, University of Turin, Torino, Italy

^2^Department of Clinical and Biological Sciences, University of Turin, Torino, Italy

^3^Department of Chemistry, University of Turin, Torino, Italy

^#^correspondign author: federica.dalbello@unito.it

**SUPPLEMENTARY MATERIALS**

| **June** | | **July** | | **August** | |
| --- | --- | --- | --- | --- | --- |
| 1 | S | 1 | **R** | 1 | S |
| 2 | **R** | 2 | S | 2 | **R** |
| 3 | S | 3 | **R** | 3 | S |
| 4 | **R** | 4 | S | 4 | S |
| 5 | S | 5 | S | 5 | S |
| 6 | S | 6 | **R** | 6 | S |
| 7 | S | 7 | **R** | 7 | **R** |
| 8 | S | 8 | S | 8 | S |
| 9 | **R** | 9 | S | 9 | S |
| 10 | S | 10 | S | 10 | S |
| 11 | **R** | 11 | **R** | 11 | S |
| 12 | **R** | 12 | **R** | 12 | S |
| 13 | S | 13 | S | 13 | S |
| 14 | **R** | 14 | S | 14 | **R** |
| 15 | **R** | 15 | S | 15 | S |
| 16 | S | 16 | S | 16 | S |
| 17 | S | 17 | S | 17 | S |
| 18 | S | 18 | S | 18 | S |
| 19 | S | 19 | S | 19 | S |
| 20 | **R** | 20 | S | 20 | S |
| 21 | **R** | 21 | S | 21 | S |
| 22 | **R** | 22 | S | 22 | S |
| 23 | **R** | 23 | S | 23 | S |
| 24 | **R** | 24 | S | 24 | S |
| 25 | **R** | 25 | S | 25 | S |
| 26 | S | 26 | S | 26 | S |
| 27 | S | 27 | S | 27 | **R** |
| 28 | S | 28 | S | 28 | S |
| 29 | **R** | 29 | S | 29 | S |
| 30 | S | 30 | S | 30 | S |
|  |  | 31 | S | 31 | S |

**Table S1**: Weather conditions during the sampling months of June, July, and August 2024. S, sun; **R**, rain.

**(i) Haloperidol hydroxylated TPs with structure loss.**

**TP_ha_286**

***Figure S1.*** MS^n^ fragmentation pattern of TP_ha_286.


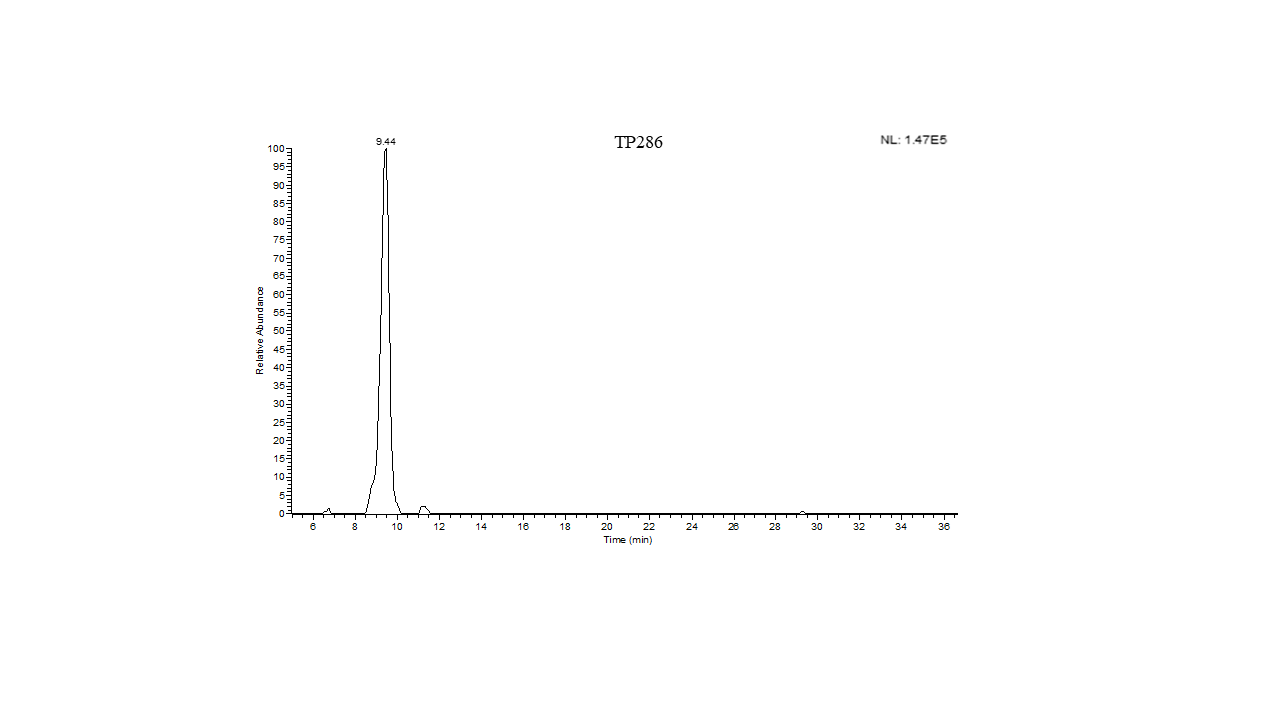


***Figure S2.*** Chromatographic separation of TP_ha_286, Rt= 9.44 min.

| TP_ha_ | [MH]^+^ | tr (min) | Δppm | MS^2^, molecular formula, (abundance %), [loss] | Δppm | MS^3^, molecular formula, (abundance %), [loss] | Δppm | MS^4^, molecular formula, (abundance %), [loss] | Δppm |
| --- | --- | --- | --- | --- | --- | --- | --- | --- | --- |
| 286 | 286.1195  [C₁₄H₂₁ClNO₃]⁺ | 9.45 | -3.3 | 268.1086, [C₁₄H₁₉ClNO₂]⁺,  (100), [-H₂O] | -4.8 | 224.0828, [C₁₂H₁₅ClNO]⁺,  (100), [-C₂H_4_O] | -3.9 | 206.0725, [C₁₂H₁₃ClN]⁺,  (100), [-H_2_O] | -2.9 |
|  |  |  |  |  |  | 206.0725, [C₁₂H₁₃ClN]⁺,  (12), [-C₂H_6_O_2_] | -2.9 |  |  |
|  |  |  |  | 224.0828, [C₁₂H₁₅ClNO]⁺,  (84), [-C₂H₆O₂] | -3.9 | 206.0725, [C₁₂H₁₃ClN]⁺,  (100), [-H_2_O] | -2.9 |  |  |
|  |  |  |  |  |  | 112.0757, [C_6_H_10_NO]⁺,  (30), [-C_6_H_5_Cl] | 0.1 |  |  |
|  |  |  |  |  |  | 84.0807, [C_5_H_10_N]⁺,  (17), [-C_7_H_5_ClO] | -0.9 |  |  |

***Table S2.*** List of MS^n^ product ions for TP_ha_286, *m/z* 286.1186.

**TP_ha_298**

***Figure S3.*** MS^n^ fragmentation pattern of TP_ha_298.


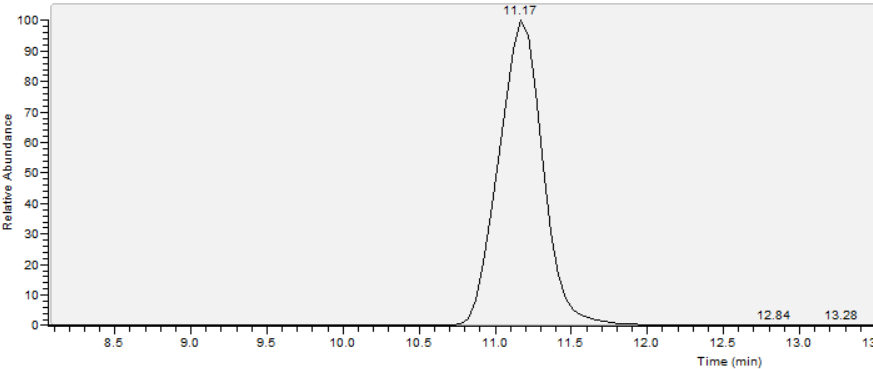


***Figure S4.*** Chromatographic separation of TP_ha_298, Rt= 11.17 min.

| TP_ha_ | [MH]^+^ | tr (min) | Δppm | MS^2^, molecular formula, (abundance %), [loss] | Δppm | MS^3^, molecular formula, (abundance %), [loss] | Δppm |
| --- | --- | --- | --- | --- | --- | --- | --- |
| 298 | 298.1200  [C_15_H_21_ClNO_3_]⁺ | 11.15 | -1.2 | 280.1092, [C_15_H_19_ClNO_2_]⁺,  (100), [-H_2_O] | -0.7 | 262.0984, [C_15_H_17_ClNO]⁺,  (46), [-H_2_O] | 0.7 |
|  |  |  |  |  |  | 234.1035, [C_14_H_17_ClN]⁺,  (46), [-CH_2_O_2_] | -0.9 |
|  |  |  |  |  |  | 194.0724, [C_11_H_13_ClN]⁺,  (100), [-C_4_H_6_O_2_] | -1.3 |
|  |  |  |  |  |  | 165.0458, [C_10_H_10_Cl]⁺,  (10), [-C_5_H_9_NO_2_] | -4.8 |
|  |  |  |  |  |  | 126.0911, [C_7_H_12_NO]⁺,  (23), [-C_8_H_7_ClO] | -2.2 |
|  |  |  |  | 194.0724, [C_11_H_13_ClN]⁺,  (54), [-C_4_H_8_O_3_] | -2.0 | \ | \ |

***Table S3.*** List of MS^n^ product ions for TP_ha_298, *m/z* 298.1200.

**TP_ha_314**

***Figure S5.*** MS^n^ fragmentation pattern of TP_ha_314-A/B/C.

***Figure S6.*** MS^n^ fragmentation pattern of TP_ha_314-D.

***Figure S7.*** MS^n^ fragmentation pattern of TP_ha_314-E.

***Figure S8.*** MS^n^ fragmentation pattern of TP_ha_314-F.


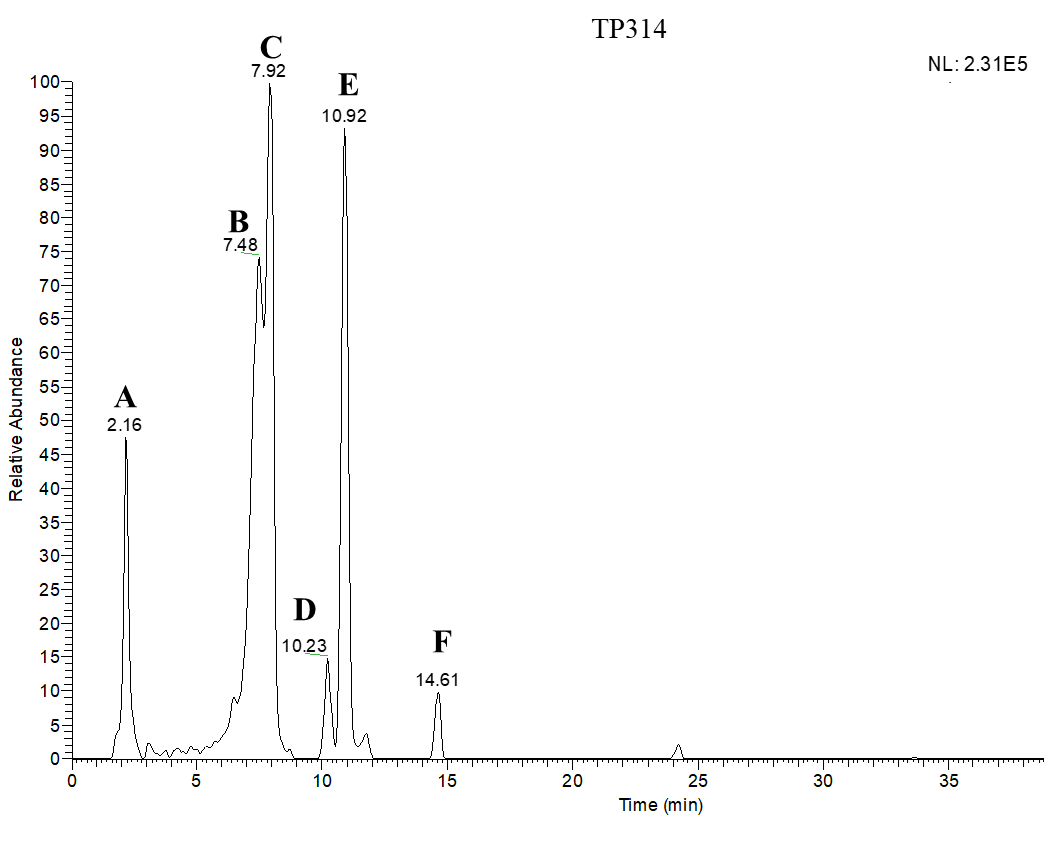


***Figure S9.*** Chromatographic separation of TP_ha_314 isomer forms, Rt= (A) 2.16, (B) 7.48, (C) 7.92, (D) 10.23, (E) 10.92 and (F) 14.61 min.

| TP_ha_ | [MH]^+^ | tr (min) | Δppm | MS^2^, molecular formula, (abundance %), [loss] | Δppm | MS^3^, molecular formula, (abundance %), [loss] | Δppm |
| --- | --- | --- | --- | --- | --- | --- | --- |
| 314-A | 314.1140 [C_15_H_21_ClNO_4_] ^+^ | 7.48 | -4.3 | 296.1034, [C_15_H_19_ClNO_3_] ^+^,  (100), [H₂O] | -4.7 | \ | \ |
|  |  |  |  | 210.0670, [C₁₁H₁₃ClNO]⁺,  (56), [-C_4_H₈O₃] | -4.8 | \ | \ |
| 314-B 314-C | 314.1140 [C_15_H_21_ClNO_4_] ^+^ | 7.92 | -4.3 | 296.1034, [C_15_H_19_ClNO_3_] ^+^,  (100), [H₂O] | -4.7 | 197.0722, [C_11_H_14_ClO]⁺,  (100), [-C_4_H_5_NO_2_] | -2.9 |
|  |  |  |  | 210.0670, [C₁₁H₁₃ClNO]⁺,  (82), [-C_4_H₈O₃] | -4.8 | \ | \ |
| 314-D | 314.1140 [C_15_H_21_ClNO_4_] ^+^ | 10.23 | -4.3 | 296.1034, [C_15_H_19_ClNO_3_] ^+^,  (100), [H₂O] | -4.7 | 278.0930, [C₁₅H₁₇ClNO₂]⁺,  (100), [-H_2_O] | -4.4 |
|  |  |  |  |  |  | 254,0935 [C_13_H₁₇ClNO₂]⁺,  (30), [-C_2_H_2_O] | -2.9 |
|  |  |  |  | 278.0930, [C₁₅H₁₇ClNO₂]⁺,  (65), [-2H_2_O] | -4.4 | 194.0728, [C₁₁H₁₃ClN]⁺,  (100), [-C_4_H_6_O_3_] | -1.6 |
|  |  |  |  | 254.0935 [C_13_H₁₇ClNO₂]⁺,  (16), [-C_2_H₄O₂] | -2.9 | \ | \ |
|  |  |  |  | 252.1137, [C₁₄H₁₉ClNO]⁺,  (30), [-CH₂O₃] | -5.0 | \ | \ |
|  |  |  |  | 194.0728, [C₁₁H₁₃ClN]⁺,  (20), [-C₄H₈O₄] | -1.6 | \ | \ |
| 314-E | 314.1140 [C_15_H_21_ClNO_4_] ^+^ | 10.92 | -4.3 | 296.1034, [C_15_H_19_ClNO_3_] ^+^,  (100), [H₂O] | -4.7 | 116.0700, [C₅H_10_NO₂]⁺,  (61), [-C₁₀H_9_ClO] | -5.2 |
|  |  |  |  |  |  | 98.0597, [C₅H₈NO],  (24), [-C_10_H_11_ClO_2_] | -3.5 |
|  |  |  |  | 116.0700, [C₅H₁₀NO₂]⁺,  (61), [-C₁₀H₁₁ClO₂] | -5.2 | \ | \ |
|  |  |  |  | 98.0597, [C₅H₈NO],  (24), [-C₁₀H₁₃ClO₃] | -3.5 | \ | \ |
| 314-F | 314.1140 [C_15_H_21_ClNO_4_] ^+^ | 14.61 | -4.3 | 224.0825, [C₁₂H₁₅ClNO]⁺,  (34), [-C₃H₆O₃] | -5.2 | 210.0670, [C₁₁H₁₃ClNO]⁺,  (100), [-CH_2_] | -4.8 |
|  |  |  |  | 210.0670, [C₁₁H₁₃ClNO]⁺,  (39), [-C₂H₈O₃] | -4.8 | \ | \ |
|  |  |  |  | 165.0459, [C₁₀H₁₀Cl]⁺,  (100), [-C₅H₁₁NO₄] | -4.0 | \ | \ |

***Table S4.*** List of MS^n^ product ions for TP_ha_314, *m/z* 314.1132.

**(ii) Haloperidol hydroxylated TPs with Cl loss.**

**TP_ha_358**

***Figure S10.*** MS^n^ fragmentation pattern of TP_ha_358.

| TP_ha_ | [MH]^+^ | tr (min) | Δppm | MS^2^, molecular formula, (abundance %), [loss] | Δppm | MS^3^, molecular formula, (abundance %), [loss] | Δppm |
| --- | --- | --- | --- | --- | --- | --- | --- |
| 358 | 358.1795  [C_21_H_25_FNO_3_]⁺ | 14.44 | -0.9 | 340.1691, [C_21_H_23_FNO_2_]⁺,  (34), [-H_2_O] | -1.7 | 194.0965, [C_11_H_13_FNO]⁺,  (52), [-C_10_H_10_O] | -1.2 |
|  |  |  |  |  |  | 165.0701, [C_10_H_10_FO]⁺,  (100), [-C_11_H_13_NO] | -1.9 |
|  |  |  |  |  |  | 123.0235, [C_7_H_4_FO]⁺,  (14), [-C_3_H_6_] | 0.2 |
|  |  |  |  | 176.1060, [C_11_H_14_NO]⁺,  (1) , [-C_10_H_11_FO_2_] | -0.7 | \ | \ |
|  |  |  |  | 165.0701, [C_10_H_10_FO]⁺,  (100), [-C_11_H_15_NO_2_] | -1.8 | 123.0235, [C_7_H_4_FO]⁺,  (17), [-C_3_H_6_] | -1.5 |
|  |  |  |  | 123.0235, [C_7_H_4_FO]⁺,  (22), [-C_14_H_21_NO_2_] | -1.5 | \ | \ |

***Table S5.*** List of MS^n^ product ions for TP_ha_358, *m/z* 358.1795.

**TP_ha_362**

***Figure S11.*** MS^n^ fragmentation pattern of TP_ha_362.

**
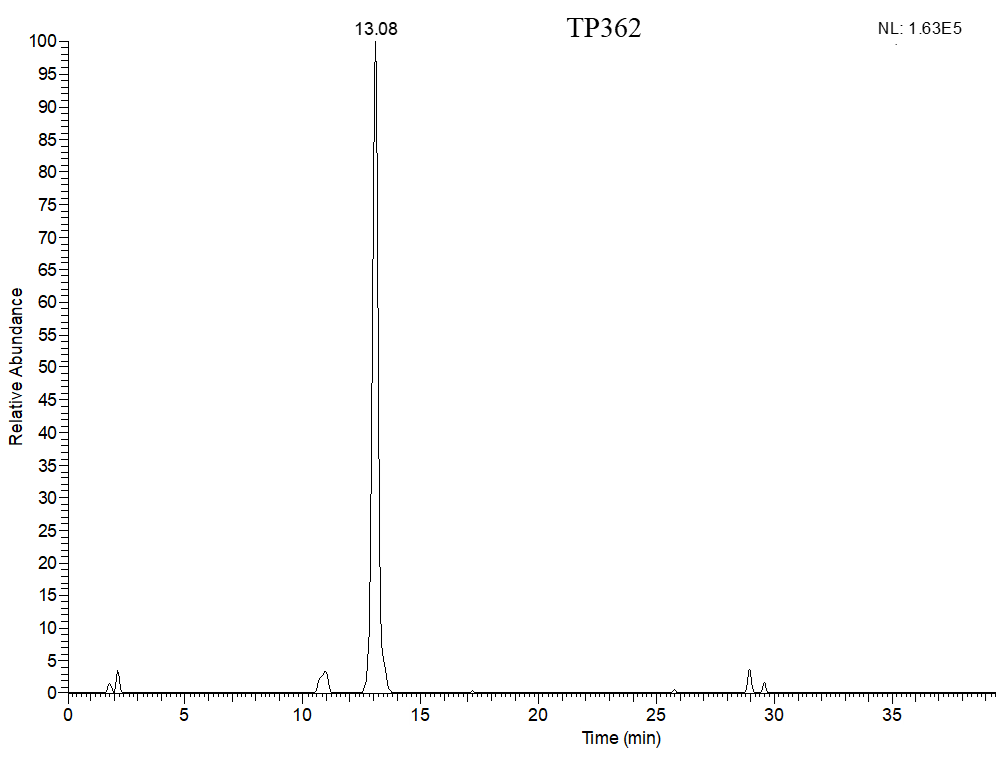
**

***Figure S12.*** Chromatographic separation of TP_ha_362, Rt= 13.08 min.

| TP_ha_ | [MH]^+^ | tr (min) | Δppm | MS^2^, molecular formula, (abundance %), [loss] | Δppm | MS^3^, molecular formula, (abundance %), [loss] | Δppm |
| --- | --- | --- | --- | --- | --- | --- | --- |
| 362 | 362.1746  [C_20_H_25_FNO_4_]⁺ | 13.08 | -4.4 | 344.1640, [C_20_H_23_FNO_3_]⁺,  (30), [-H_2_O] | -4.8 | 326.1548, [C_20_H_21_FNO_2_]⁺,  (100), [-H_2_O] | -0.9 |
|  |  |  |  |  |  | 244.1132, [C_15_H_15_FNO]⁺,  (28), [-C_5_H_8_O_2_] | -0.1 |
|  |  |  |  |  |  | 218.0974, [C_13_H_13_FNO]⁺,  (19), [-C_7_H_10_O_2_] | -1 |
|  |  |  |  |  |  | 165.0712, [C_10_H_10_FO]⁺,  (31), [-C_10_H_13_NO_2_] | -0.8 |
|  |  |  |  | 165.0712, [C_10_H_10_FO]⁺,  (100), [-C_10_H_15_NO_3_] | 1.1 | 123.0242, [C_7_H_4_FO]⁺,  (100), [-C_3_H_6_] | 1.1 |
|  |  |  |  | 123.0242, [C_7_H_4_FO]⁺,  (21), [-C_13_H_21_NO_3_] | 1.1 | \ | \ |

***Table S6.*** List of MS^n^ product ions for TP_ha_362, *m/z* 362.1746.

**TP_ha_388**

***Figure S13.*** MS^n^ fragmentation pattern of TP_ha_388-A/B.

**
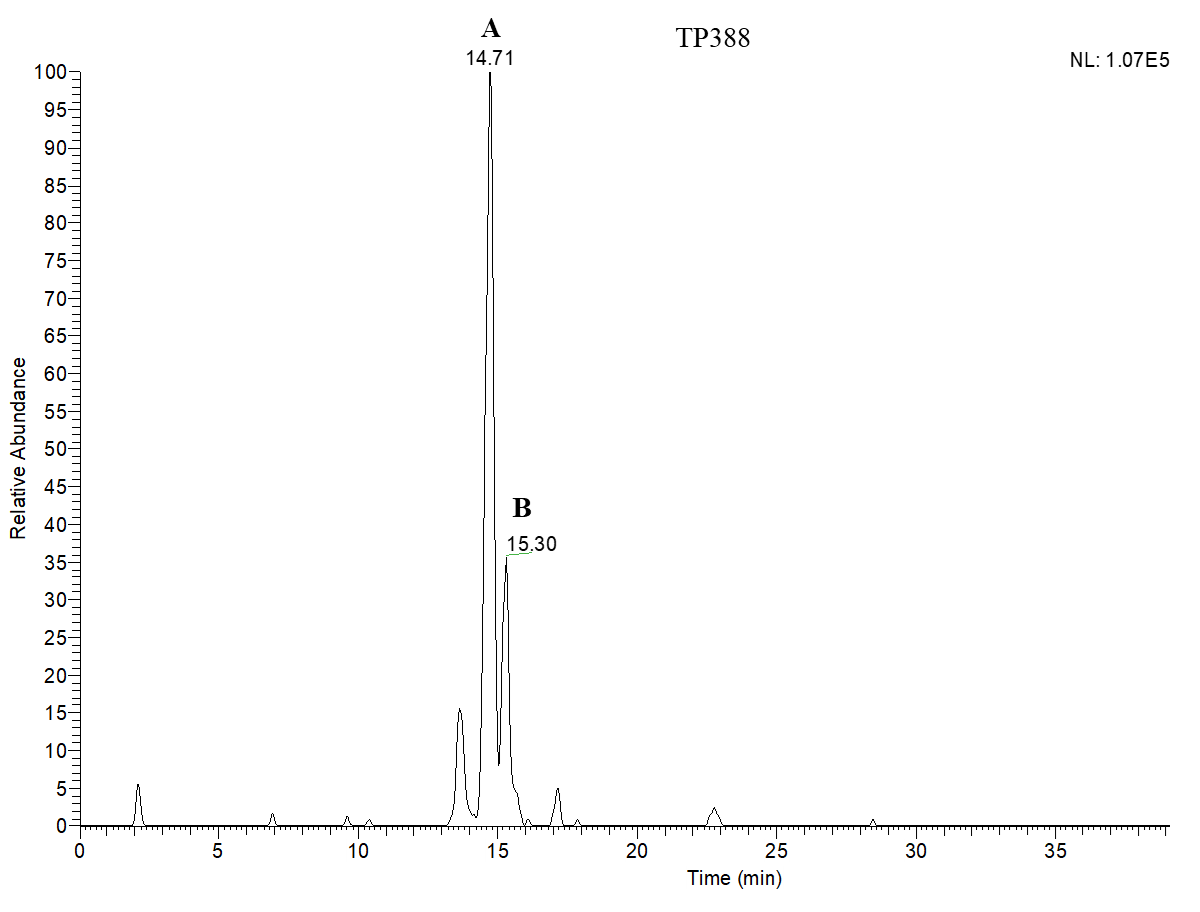
**

***Figure S14.*** Chromatographic separation of TP_ha_388 isomer forms, Rt= (A) 14.71 and (B) 15.30 min.

| TP_ha_ | [MH]^+^ | tr (min) | Δppm | MS^2^, molecular formula, (abundance %), [loss] | Δppm | MS^3^, molecular formula, (abundance %), [loss] | Δppm |
| --- | --- | --- | --- | --- | --- | --- | --- |
| 388-A | 388.1544  [C_21_H_23_FNO_5_]⁺ | 14.71 | -2.8 | 370.1432, [C_21_H_21_FNO_4_]⁺,  (1), [-H_2_O] | -4.6 | 194.0967, [C_11_H_13_FNO]⁺,  (50), [-C_10_H_8_O_3_] | -4.5 |
|  |  |  |  |  |  | 165.0701, [C_10_H_10_FO]⁺, (100), [-C_11_H_11_NO_3_] | -4.5 |
|  |  |  |  |  |  | 123.0235, [C_7_H_4_FO]⁺,  (19), [-C_14_H_17_NO_3_] | -4.4 |
|  |  |  |  | 224.0907, [C_11_H_14_NO_4_]⁺,  (1) , [-C_10_H_8_FO] | -4.6 | \ | \ |
|  |  |  |  | 165.0701, [C_10_H_10_FO]⁺,  (100), [-C_11_H_13_NO_4_] | -4.5 | 123.0235, [C_7_H_4_FO]⁺,  (100), [-C_3_H_6_] | -4.4 |
|  |  |  |  | 123.0235, [C_7_H_4_FO]⁺,  (19), [-C_14_H_19_NO_4_] | -4.4 | \ | \ |
| 388-B | 388.1544  [C_21_H_23_FNO_5_]⁺ | 15.28 | -2.8 | 165.0701, [C_10_H_10_FO]⁺,  (100), [-C_11_H_13_NO_4_] | -4.5 | 123.0235, [C_7_H_4_FO]⁺,  (100), [-C_3_H_6_] | -4.4 |
|  |  |  |  | 123.0235, [C_7_H_4_FO]⁺,  (19), [-C_14_H_19_NO_4_] | -4.4 | \ | \ |

***Table S7.*** List of MS^n^ product ions for TP_ha_388, *m/z* 388.1544.

**TP_ha_390**

***Figure S15.*** MS^n^ fragmentation pattern of TP_ha_390-A.

***Figure S16.*** MS^n^ fragmentation pattern of TP_ha_390-B.

***
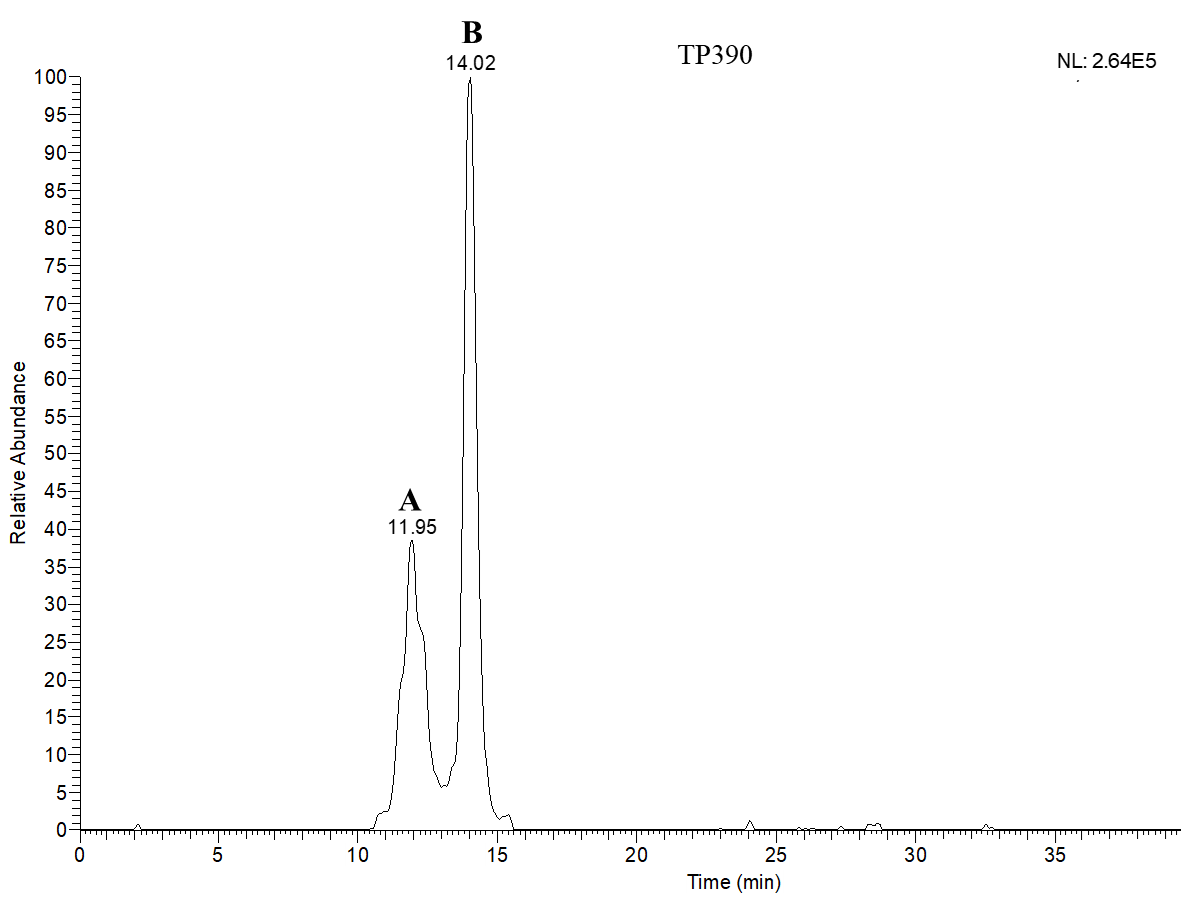
***

***Figure S17.*** Chromatographic separation of TP_ha_390 isomer forms, Rt= (A) 11.95 and (B) 14.02 min.

| TP_ha_ | [MH]^+^ | tr (min) | Δppm | MS^2^, molecular formula, (abundance %), [loss] | Δppm | MS^3^, molecular formula, (abundance %), [loss] | Δppm |
| --- | --- | --- | --- | --- | --- | --- | --- |
| 390-A | 390.1705  [C_21_H_25_FNO_5_]⁺ | 11.95 | -1.6 | 372.1599, [C_21_H_23_FNO_4_]⁺,  (18), [-H_2_O] | -1.8 | 165.0708, [C_10_H_10_FO]⁺,  (100), [-C_11_H_15_NO_4_] | -1.3 |
|  |  |  |  |  |  | 123.0239, [C_7_H_4_FO]⁺,  (32), [-C_14_H_19_NO_3_] | -1.4 |
|  |  |  |  | 264.1390, [C_15_H_19_FNO_2_]⁺,  (3), [-C_6_H_6_O_3_] | -1.6 | 165.0708, [C_10_H_10_FO]⁺,  (100), [-C_11_H_15_NO_4_] | -1.3 |
|  |  |  |  | 226.1070, [C_11_H_16_NO_4_]⁺,  (2), [-C_10_H_9_FO] | -1.7 | \ | \ |
|  |  |  |  | 165.0708, [C_10_H_10_FO]⁺,  (100), [-C_11_H_15_NO_4_] | -1.3 | 123.0239, [C_7_H_4_FO]⁺,  (100), [-C_3_H_6_] | -1.4 |
|  |  |  |  | 123.0239, [C_7_H_4_FO]⁺,  (20), [-C_14_H_21_NO_4_] | -1.4 | \ | \ |
| 390-B | 390.1700  [C_21_H_25_FNO_5_]⁺ | 14.02 | -1.6 | 372.1599, [C_21_H_23_FNO_4_]⁺,  (19), [-H_2_O] | -1.8 | 354.1493, [C_21_H_21_FNO_3_]⁺,  (14), [-2H_2_O] | -1.9 |
|  |  |  |  |  |  | 208.0965, [C_11_H_14_NO_3_]⁺,  (19), [-C_10_H_9_FO] | -1.5 |
|  |  |  |  |  |  | 165.0708, [C_10_H_10_FO]⁺,  (74), [-C_11_H_15_NO_4_] | -1.3 |
|  |  |  |  |  |  | 123.0239, [C_7_H_4_FO]⁺,  (24), [-C_14_H_19_NO_3_] | -1.4 |
|  |  |  |  | 354.1493, [C_21_H_21_FNO_3_]⁺,  (2), [-2H_2_O] | -1.9 | \ | \ |
|  |  |  |  | 226.1070, [C_11_H_16_NO_4_]⁺,  (6), [-C_10_H_9_FO] | -1.7 | 208.0957, [C_11_H_14_NO_3_]⁺,  (100), [-H_2_O] | X |
|  |  |  |  | 208.0957, [C_11_H_14_NO_3_]⁺,  (3), [-C_10_H_11_FO_2_] | -3.9 | \ | \ |
|  |  |  |  | 165.0708, [C_10_H_10_FO]⁺,  (100), [-C_11_H_15_NO_4_] | -1.3 | 123.0239, [C_7_H_4_FO]⁺,  (100), [-C_3_H_6_] | -1.4 |
|  |  |  |  | 123.0239, [C_7_H_4_FO]⁺,  (21), [-C_14_H_21_NO_4_] | -1.4 | \ | \ |

***Table S8.*** List of MS^n^ product ions for TP_ha_390, *m/z* 390.1700.

**TP_ha_406**

***Figure S18.*** MS^n^ fragmentation pattern of TP_ha_406-A.

***Figure S19.*** MS^n^ fragmentation pattern of TP_ha_406-B.


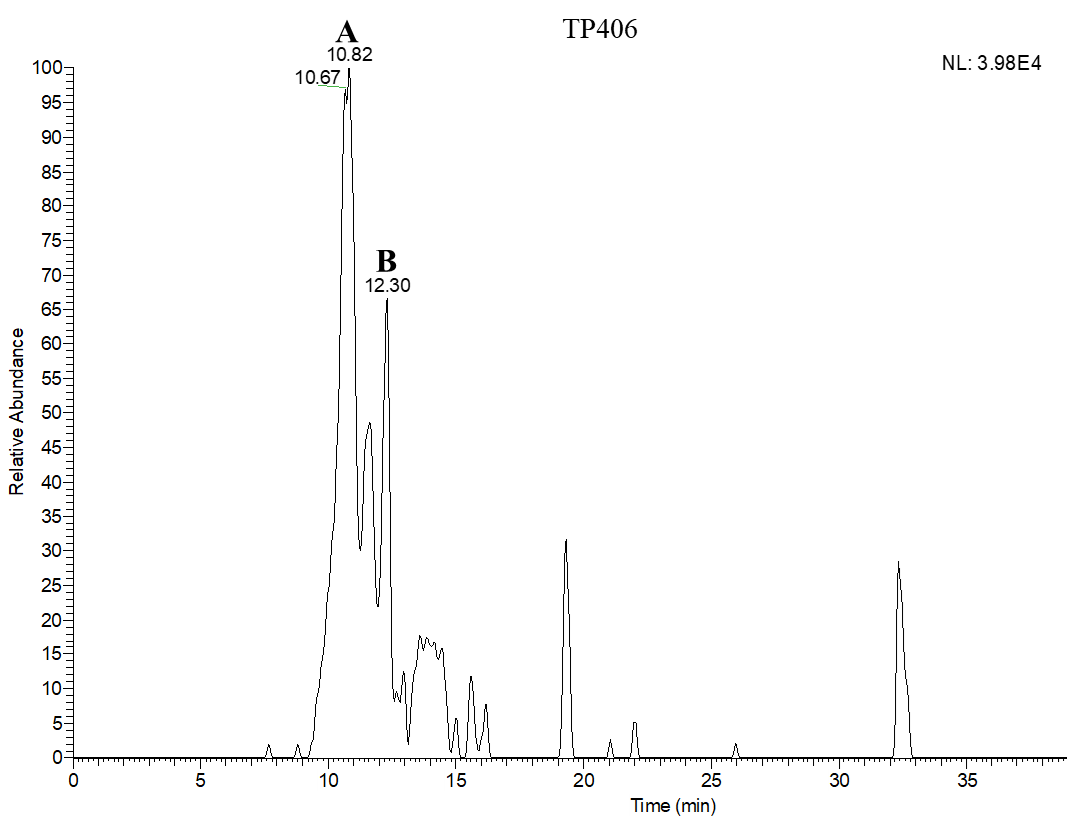


***Figure S20.*** Chromatographic separation of TP_ha_406 isomer forms, Rt = (A) 10.82 and (B) 12.30 min.

| TP_ha_ | [MH]^+^ | tr (min) | Δppm | MS^2^, molecular formula, (abundance %), [loss] | Δppm | MS^3^, molecular formula, (abundance %), [loss] | Δppm |
| --- | --- | --- | --- | --- | --- | --- | --- |
| 406-A | 406.1645  [C_21_H_25_FNO_6_]⁺ | 10.82 | -3.8 | 388.1539, [C_21_H_23_FNO_5]_⁺,  (32), [-H_2_O] | -4.0 | \ | \ |
|  |  |  |  | 242.1013, [C_11_H_16_NO_5_]⁺,  (8), [-C_10_H_9_FO] | -4.1 | \ | \ |
|  |  |  |  | 165.0705, [C_10_H_10_FO]⁺,  (100), [-C_11_H_15_NO_5_] | -3.8 | 123.0235, [C_7_H_4_FO]⁺,  (100), [-C_3_H_6_] | -4.6 |
|  |  |  |  | 123.0235, [C_7_H_4_FO]⁺,  (17), [-C_14_H_11_NO_5_] | -4.6 | \ | \ |
| 406-B | 406.1645 [C_21_H_25_FNO_6_]⁺ | 12.3 | -3.8 | 388.1539, [C_21_H_23_FNO_5]_⁺,  (32), [-H_2_O] | -4.0 | 370.1436, [C_21_H_21_FNO_4_]⁺,  (100), [-H_2_O] | -3.5 |
|  |  |  |  | 370.1436, [C_21_H_21_FNO_4_]⁺,  (9), [-2H_2_O] | -3.5 | \ | \ |
|  |  |  |  | 224.0910, [C_11_H_14_NO_4_]⁺,  (19), [-C_10_H_11_FO_2_] | -3.3 | 206.0805, [C_11_H_12_NO_3_]⁺,  (100), [-H_2_O] | -2.2 |
|  |  |  |  | 206.0805, [C_11_H_12_NO_3_]⁺,  (12), [-C_10_H_19_NO_4_] | -2.2 | \ | \ |
|  |  |  |  | 165.0705, [C_10_H_10_FO]⁺,  (100), [-C_11_H_15Cl_NO_5_] | -3.8 | 123.0235, [C_7_H_4_FO]⁺,  (100), [-C_3_H_6_] | -4.6 |
|  |  |  |  | 123.0235, [C_7_H_4_FO]⁺,  (20), [-C_14_H_11_ClNO_5_] | -4.6 | \ | \ |

***Table S9.*** List of MS^n^ product ions for TP_ha_406, *m/z* 406.1645.

**(iii) Haloperidol hydroxylated TPs with F loss**

**TP_ha_374**

***Figure S21.*** MS^n^ fragmentation pattern of TP_ha_374.


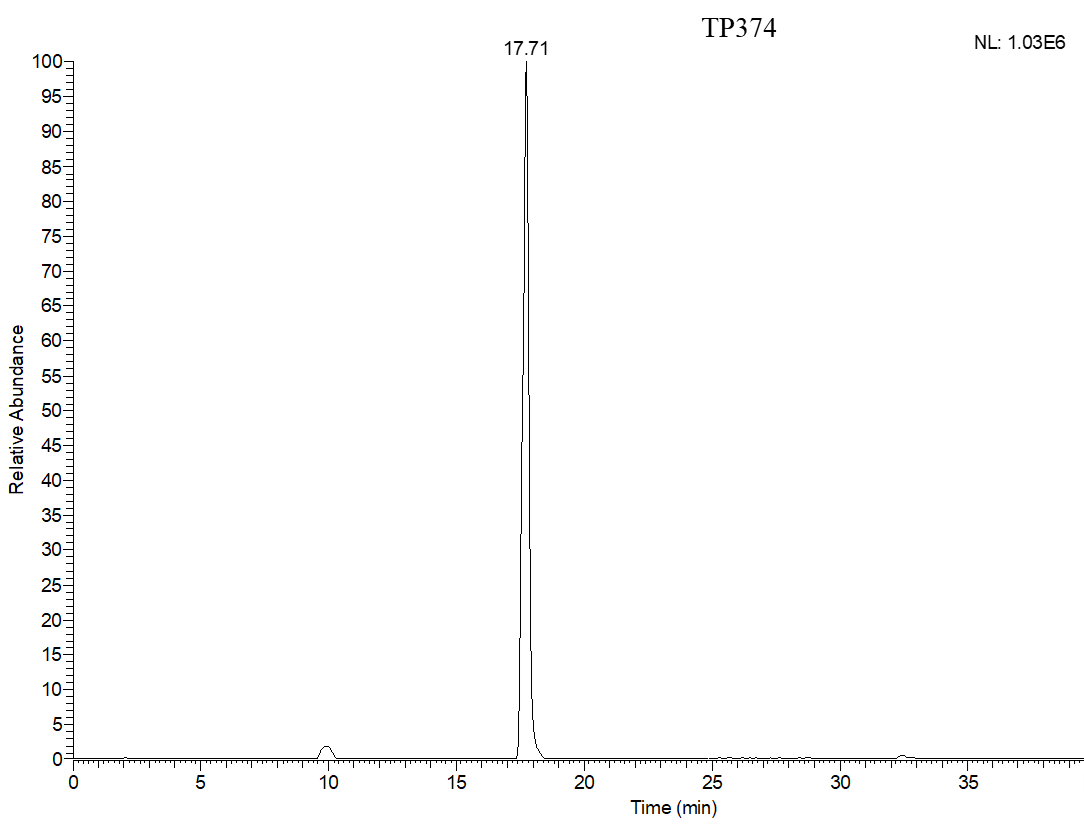


***Figure S22.*** Chromatographic separation of TP_ha_374, Rt = 17.71 min.

| TP_ha_ | [MH]^+^ | tr (min) | Δppm | MS^2^, molecular formula, (abundance %), [loss] | Δppm | MS^3^, molecular formula, (abundance %), [loss] | Δppm |
| --- | --- | --- | --- | --- | --- | --- | --- |
| 374 | 374.1503  [C_21_H_25_ClNO_3_]⁺ | 17.71 | -0.9 | 356.1398, [C_21_H_23_ClNO_2_]⁺,  (100), [-H_2_O] | -1.2 | 194.0721, [C_11_H_13_ClN]⁺,  (57), [-C_10_H_10_O_2_] | -0.7 |
|  |  |  |  |  |  | 192.1009, [C_11_H_14_NO_2_]⁺,  (27), [-C_10_H_9_Cl] | -0.9 |
|  |  |  |  |  |  | 163.0746, [C_10_H_11_O_2_]⁺,  (100), [-C_11_H_12_ClN] | -0.9 |
|  |  |  |  |  |  | 161.0596, [C_10_H_9_O_2_]⁺,  (12), [-C_11_H_12_ClN] | -0.9 |
|  |  |  |  |  |  | 145.0648, [C_10_H_9_O]⁺,  (6), [-C_11_H_14_ClNO] | -0.1 |
|  |  |  |  |  |  | 133.0647, [C_9_H_8_O],  (3), [-C_12_H_15_ClNO] | -0.9 |
|  |  |  |  |  |  | 121.0278, [C_7_H_5_O_2_]⁺,  (7), [-C_14_H_18_ClN] | -0.5 |
|  |  |  |  | 194.0721, [C_11_H_13_ClN]⁺,  (3), [-C_10_H_12_O_3_] | -3.2 | \ | \ |
|  |  |  |  | 192.1009, [C_11_H_14_NO_2_]⁺,  (7), [-C_10_H_11_ClO] | -1.2 | 163.0746, [C_10_H_11_O_2_]⁺,  (100), [-CH_3_] | -2.1 |
|  |  |  |  | 163.0746, [C_10_H_11_O_2_]⁺,  (94), [-C_11_H_14_ClNO] | -1.8 | 121.0278, [C_7_H_5_O_2_]⁺,  (100), [-C_3_H_6_] | -2 |
|  |  |  |  | 121.0278, [C_7_H_5_O_2_]⁺,  (16), [-C_14_H_20_ClNO] | -1.5 | \ | \ |

***Table S10.*** List of MS^n^ product ions for TP_ha_374, *m/z* 374.1503.

**(iv) Haloperidol hydroxylated TPs.**

**TP_ha_392**

***Figure S23.*** MS^n^ fragmentation pattern of TP_ha_392-A/C.

***Figure S24.*** MS^n^ fragmentation pattern of TP_ha_392-B.

***Figure S25.*** MS^n^ fragmentation pattern of TP_ha_392-D.

**
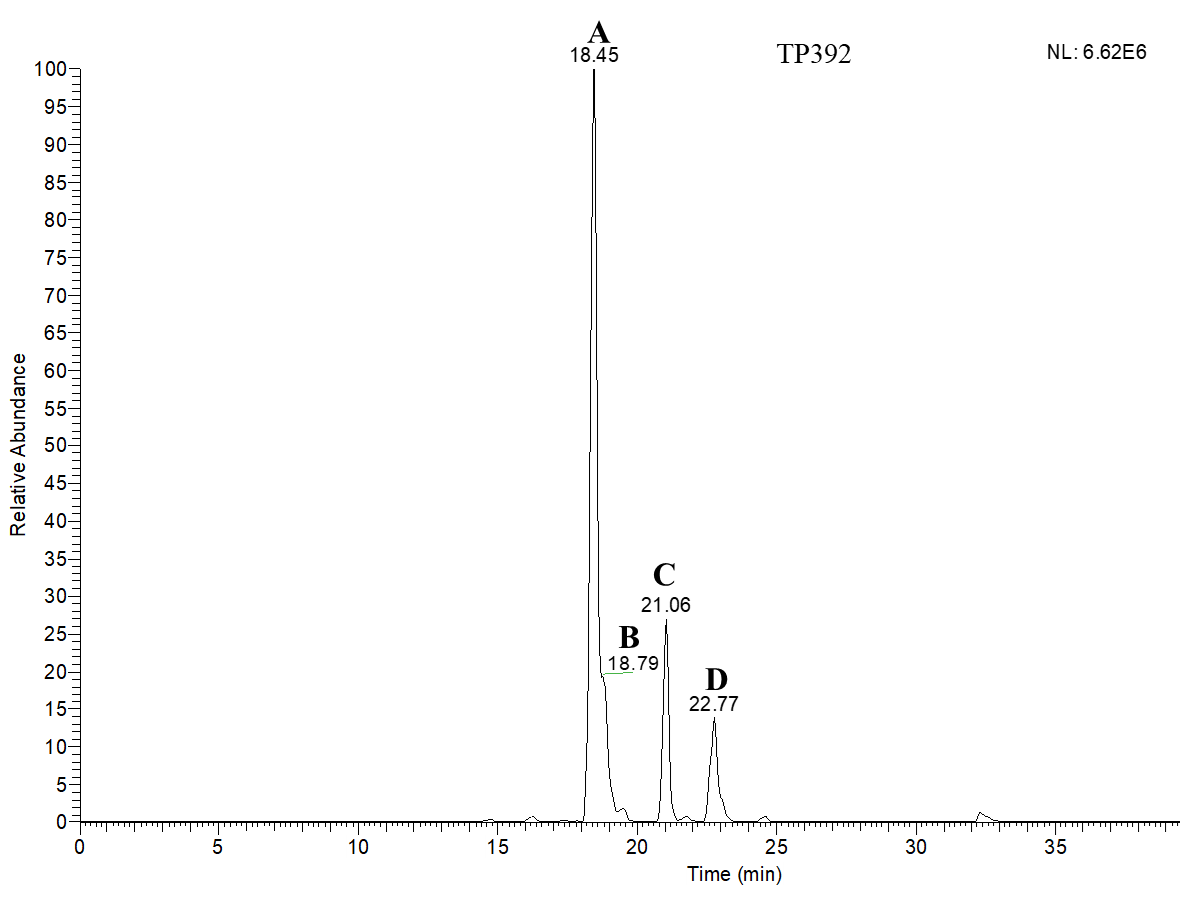
**

***Figure S26.*** Chromatographic separation of TP_ha_392 isomer forms, Rt = (A) 18.45, (B) 18.79, (C) 21.06 and (D) 22.77 min.

| TP_ha_ | [MH]^+^ | tr (min) | Δppm | MS^2^, molecular formula, (abundance %), [loss] | Δppm | MS^3^, molecular formula, (abundance %), [loss] | Δppm |
| --- | --- | --- | --- | --- | --- | --- | --- |
| 392-A | 392.1416  [C_21_H_24_ClFNO_3_]⁺ | 18.45 | -1.8 | 374.1310, [C_21_H_22_ClFNO_2_]⁺,  (53), [-H_2_O] | -2.0 | 210.0675, [C_11_H_13_ClNO]⁺,  (91), [-C_10_H_9_FO] | -2.4 |
|  |  |  |  |  |  | 194.0971, [C_11_H_13_FNO],  (29), [-C_10_H_9_ClO] | -2.4 |
|  |  |  |  |  |  | 165.0706, [C_10_H_10_FO]⁺,  (100), [-C_11_H_12_ClNO] | -2.5 |
|  |  |  |  |  |  | 123.0238, [C_7_H_4_FO]⁺,  (15), [-C_14_H_18_ClNO] | -2.1 |
|  |  |  |  | 228.0780, [C_11_H_15_ClNO_2_]⁺,  (1), [-C_10_H_9_FO] | -2.5 | 210.0675, [C_11_H_13_ClNO]⁺,  (100), [-H_2_O] | -2.4 |
|  |  |  |  | 210.0675, [C_11_H_13_ClNO]⁺,  (7), [-C_10_H_11_FO_2_] | -2.4 | \ | \ |
|  |  |  |  | 194.0971, [C_11_H_13_FNO],  (2), [-C_10_H_11_ClO_2_] | -2.4 | 165.0706, [C_10_H_10_FO]⁺,  (100), [-CH_3_N] | -2.5 |
|  |  |  |  | 165.0706, [C_10_H_10_FO]⁺,  (100), [-C_11_H_14_ClNO_2_] | -2.5 | 123.0238, [C_7_H_4_FO]⁺,  (100), [-C_3_H_6_] | -2.1 |
|  |  |  |  | 123.0238, [C_7_H_4_FO]⁺,  (25), [-C_14_H_20_ClNO_2_] | -2.1 | \ | \ |
| 392-B | 392.1416  [C_21_H_24_ClFNO_3_]⁺ | 18.79 | -1.8 | 374.1310, [C_21_H_22_ClFNO_2_]⁺,  (100), [-H_2_O] | -2.0 | 356.1207, [C_21_H_20_ClFNO]⁺,  (16), [-H_2_O] | -1.3 |
|  |  |  |  |  |  | 236.0832, [C_13_H_15_ClNO]⁺,  (26), [-C_8_H_7_FO] | -1.9 |
|  |  |  |  |  |  | 210.0924, [C_11_H_13_FNO_2_]⁺,  (37), [-C_10_H_9_Cl] | -0.4 |
|  |  |  |  |  |  | 208.0886, [C_12_H_15_ClN]⁺,  (100), [-C_9_H_7_FO] | -0.7 |
|  |  |  |  |  |  | 194.0728, [C_11_H_13_ClN]⁺,  (5), [-C_10_H_9_FO_2_] | -1.6 |
|  |  |  |  |  |  | 192.0816, [C_11_H_11_FNO]⁺,  (7), [-C_10_H_11_ClO] | -1.7 |
|  |  |  |  |  |  | 179.0502, [C_10_H_8_FO_2_]⁺,  (8), [-C_11_H_14_ClN] | -0.5 |
|  |  |  |  |  |  | 181.0657, [C_10_H_10_FO_2_]⁺,  (4), [-C_11_H_12_ClN] | -1.3 |
|  |  |  |  |  |  | 163.0553, [C_10_H_8_FO]⁺, (2), [-C_11_H_14_ClNO] | -0.4 |
|  |  |  |  |  |  | 151.0550, [C_9_H_8_FO]⁺,  (2), [-C_12_H_14_ClNO] | -2.5 |
|  |  |  |  |  |  | 125.0153, [C_7_H_6_Cl]⁺,  (1), [-C_14_H_16_FO] | 0.4 |
|  |  |  |  | 356.1207, [C_21_H_20_ClFNO]⁺,  (5), [-2H_2_O] | -1.3 | \ | \ |
|  |  |  |  | 254.0942, [C_13_H_17_ClNO_2_],  (12), [-C_8_H_7_FO] | -0.1 | \ | \ |
|  |  |  |  | 236.0832, [C_13_H_15_ClNO]⁺,  (2), [-C_8_H_9_FO_2_] | -1.9 | \ | \ |
|  |  |  |  | 222.0921, [C_12_H_13_FNO_2_]⁺,  (5), [-C_9_H_11_ClO] | -1.7 | 163.0553, [C_10_H_8_FO]⁺, (100), [-C_2_H_5_NO] | -0.4 |
|  |  |  |  | 210.0924, [C_11_H_13_FNO_2_]⁺,  (7), [-C_10_H_11_FO_2_] | -0.4 | 192.0816, [C_11_H_11_FNO]⁺,  (58), [-CH_2_O] | -1.7 |
|  |  |  |  |  |  | 163.0553, [C_10_H_8_FO]⁺, (100), [-CH_5_ClNO] | -0.4 |
|  |  |  |  | 194.0728, [C_11_H_13_ClN]⁺,  (1), [-C_10_H_11_FO_3_] | -1.6 | \ | \ |
|  |  |  |  | 181.0657, [C_10_H_10_FO_2_]⁺,  (6), [-C_11_H_14_ClNO] | -1.3 | 163.0553, [C_10_H_8_FO]⁺, (100), [-H_2_O] | -0.4 |
|  |  |  |  | 163.0553, [C_10_H_8_FO]⁺, (13), [-C_11_H_16_ClNO_2_] | -0.4 | 123.0238, [C_7_H_4_FO]⁺,  (100), [-C_3_H_4_] | -2.1 |
|  |  |  |  | 123.0238, [C_7_H_4_FO]⁺,  (1), [-C_14_H_20_ClNO_2_] | -2.1 | \ | \ |
| 392-C | 392.1416  [C_21_H_24_ClFNO_3_]⁺ | 21.05 | -1.8 | 374.1310, [C_21_H_22_ClFNO_2_]⁺,  (66), [-H_2_O] | -2.0 | 210.0675, [C_11_H_13_ClNO]⁺,  (42), [-C_10_H_9_FO] | -2.4 |
|  |  |  |  |  |  | 194.0971, [C_11_H_13_FNO],  (82), [-C_10_H_9_ClO] | -2.4 |
|  |  |  |  |  |  | 165.0706, [C_10_H_10_FO]⁺,  (100), [-C_11_H_12_ClNO] | -2.5 |
|  |  |  |  |  |  | 123.0238, [C_7_H_4_FO]⁺,  (12), [-C_14_H_18_ClNO] | -2.1 |
|  |  |  |  | 210.0675, [C_11_H_13_ClNO]⁺,  (5), [-C_10_H_11_FO_2_] | -2.4 | \ | \ |
|  |  |  |  | 194.0971, [C_11_H_13_FNO],  (16), [-C_10_H_11_ClO_2_] | -2.4 | 165.0706, [C_10_H_10_FO]⁺,  (100), [-CH_3_N] | -2.5 |
|  |  |  |  | 165.0706, [C_10_H_10_FO]⁺,  (100), [-C_11_H_14_ClNO_2_] | -2.5 | 123.0238, [C_7_H_4_FO]⁺,  (100), [-C_3_H_6_] | -2.1 |
|  |  |  |  | 123.0238, [C_7_H_4_FO]⁺,  (6), [-C_14_H_20_ClNO_2_] | -2.1 | \ | \ |
| 392-D | 392.1416  [C_21_H_24_ClFNO_3_]⁺ | 22.76 | -1.8 | 374.1310, [C_21_H_22_ClFNO_2_]⁺,  (95), [-H_2_O] | -2.0 | 210.0924, [C_11_H_13_FNO_2_]⁺,  (28), [-C_10_H_9_Cl] | -0.4 |
|  |  |  |  |  |  | 194.0971, [C_11_H_13_FNO],  (76), [-C_10_H_9_ClO] | -2.4 |
|  |  |  |  |  |  | 181.0657, [C_10_H_10_FO_2_]⁺,  (100), [-C_11_H_12_ClN] | -1.3 |
|  |  |  |  |  |  | 139.0188, [C_7_H_4_FO_2_]⁺,  (23),[-C_14_H_18_ClN]⁺ | -1.3 |
|  |  |  |  |  |  | 125.0395, [C_7_H_6_FO]⁺,  (5), [-C_14_H_16_ClNO] | -1.8 |
|  |  |  |  | 194.0973, [C_11_H_13_FNO],  (7), [-C_10_H_11_ClO_2_] | -1.4 | \ | \ |
|  |  |  |  | 181.0657, [C_10_H_10_FO_2_]⁺,  (100), [-C_11_H_14_ClNO] | -1.3 | 139.0188, [C_7_H_4_FO_2_]⁺,  (100),[-C_3_H_6_]⁺ | -1.3 |
|  |  |  |  | 139.0188, [C_7_H_4_FO_2_]⁺,  (27),[-C_14_H_20_ClNO]⁺ | -1.3 | \ | \ |

***Table S11.*** List of MS^n^ product ions for TP_ha_392, *m/z* 392.1416.

**TP_ha_408**

***Figure S27.*** MS^n^ fragmentation pattern of TP_ha_408-A/C.

***Figure S28.*** MS^n^ fragmentation pattern of TP_ha_408-B.

***Figure S29.*** MS^n^ fragmentation pattern of TP_ha_408-D.


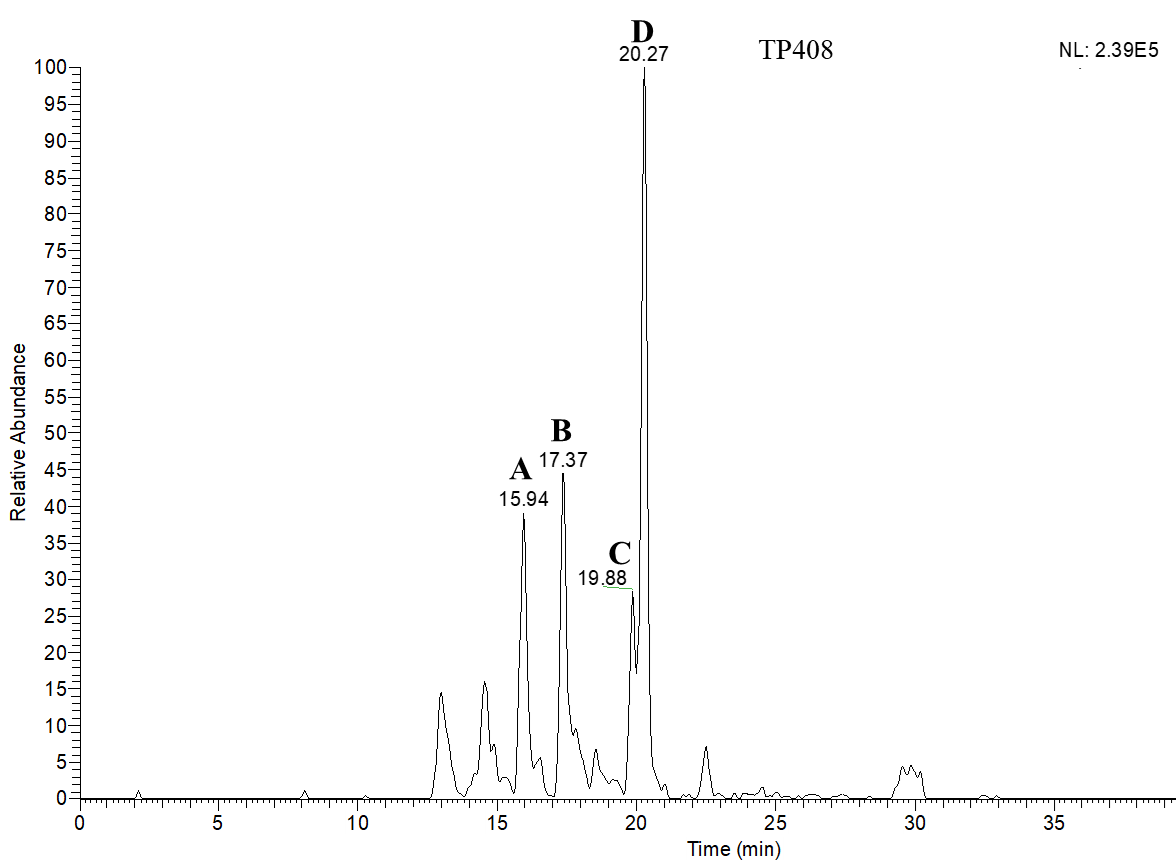


***Figure S30.*** Chromatographic separation of TP_ha_408 isomer forms, Rt= (A) 15.94, (B) 17.37, (C) 19.88 and (D) 20.27 min.

| TP_ha_ | [MH]^+^ | tr (min) | Δppm | MS^2^, molecular formula, (abundance %), [loss] | Δppm | MS^3^, molecular formula, (abundance %), [loss] | Δppm |
| --- | --- | --- | --- | --- | --- | --- | --- |
| 408-A | 408.1358  [C_21_H_24_ClFNO_4_]⁺ | 15.94 | -2.8 | 390.1256, [C_21_H_22_ClFNO_3_]⁺,  (33), [-H_2_O] | -2.7 | 372.1144, [C_21_H_20_ClFNO_2_]⁺,  (X), [-H_2_O] | -5.9 |
|  |  |  |  |  |  | 224.0837, [C_12_H_15_ClNO]⁺,  (100), [-C_9_H_7_FO_2_] | X |
|  |  |  |  | 372.1144, [C_21_H_20_ClFNO_2_]⁺,  (1), [-2H_2_O] | -5.9 | \ | \ |
|  |  |  |  | 240.0772, [C_12_H_15_ClNO_2_],  (1), [-C_9_H_9_FO_2_] | -5.7 | \ | \ |
|  |  |  |  | 210.0670, [C_11_H_13_ClNO]⁺,  (3), [-C_10_H_11_FO_3_] | -4.8 | \ | \ |
|  |  |  |  | 181.0650, [C_10_H_10_FO_2_]⁺,  (100), [-C_11_H_14_ClO_2_] | -5.1 | 139.0183, [C_7_H_4_FO_2_]⁺,  (100), [-C_3_H_6_] | X |
|  |  |  |  | 139.0183, [C_7_H_4_FO_2_]⁺,  (24), [-C_14_H_20_ClO_2_] | -5.2 | \ | \ |
| 408-B | 408.1358  [C_21_H_24_ClFNO_4_]⁺ | 17.41 | -4.9 | 390.1256, [C_21_H_22_ClFNO_3_]⁺,  (41), [-H_2_O] | -4.3 | 226.0618, [C_11_H_13_ClNO_2_]⁺,  (5), [-C_10_H_9_FO] | X |
|  |  |  |  |  |  | 194.0964, [C_11_H_13_FNO],  (8), [-C_10_H_9_ClO_2_] | X |
|  |  |  |  |  |  | 165.0701, [C_10_H_10_FO]⁺, (100), [-C_11_H_12_ClNO_2_] | X |
|  |  |  |  |  |  | 123.0235, [C_7_H_4_FO]⁺,  (21), [-C_14_H_18_ClNO_2_] | X |
|  |  |  |  | 244.0723, [C_11_H_15_ClNO_3_]_,_  (2), [-C_10_H_9_FO] | -4.9 | 226.0618, [C_11_H_13_ClNO_2_]⁺,  (100), [-H_2_O] | X |
|  |  |  |  | 226.0618, [C_11_H_13_ClNO_2_]⁺,  (5), [-C_10_H_11_FO_2_] | -5.2 | \ | \ |
|  |  |  |  | 194.0964, [C_11_H_13_FNO],  (8), [-C_10_H_11_ClO_3_] | -6.1 | 165.0701, [C_10_H_10_FO]⁺,  (100), [-CH_3_N] | -5.5 |
|  |  |  |  |  |  | 123.0235, [C_7_H_4_FO]⁺,  (21), [-C_4_H_7_N] | -4.9 |
|  |  |  |  | 165.0701, [C_10_H_10_FO]⁺,  (100), [-C_11_H_14_ClNO_3_] | -5.5 | 123.0235, [C_7_H_4_FO]⁺,  (100), [-C_3_H_6_] | -4.9 |
|  |  |  |  | 123.0235, [C_7_H_4_FO]⁺,  (21), [-C_14_H_20_ClNO_3_] | -4.9 | \ | \ |
| 408-C | 408.1358  [C_21_H_24_ClFNO_4_]⁺ | 19.87 | -5.8 | 390.1256, [C_21_H_22_ClFNO_3_]⁺,  (56), [-H_2_O] | -4.9 | 372.1144, [C_21_H_20_ClFNO_2_]⁺,  (100), [-H_2_O] | -5.9 |
|  |  |  |  | 210.0670, [C_11_H_13_ClNO]⁺,  (6), [-C_10_H_11_FO_3_] | -5.7 | \ | \ |
|  |  |  |  | 181.0650, [C_10_H_10_FO_2_]⁺,  (100), [-C_11_H_14_ClO_2_] | -4.9 | 139.0183, [C_7_H_4_FO_2_]⁺,  (24), [-C_14_H_20_ClO_2_] | -4.4 |
|  |  |  |  | 139.0183, [C_7_H_4_FO_2_]⁺,  (24), [-C_14_H_20_ClO_2_] | -4.4 | \ | \ |
| 408-D | 408.1358  [C_21_H_24_ClFNO_4_]⁺ | 20.31 | -4.9 | 390.1256, [C_21_H_22_ClFNO_3_]⁺,  (89), [-H_2_O] | -5.1 | 226.0862, [C_11_H_13_FNO_3_]⁺,  (30), [C_10_H_9_Cl] | -5.4 |
|  |  |  |  |  |  | 197.0598, [C_10_H_10_FO_3_]⁺,  (100), [-CH_3_N] | -5.4 |
|  |  |  |  |  |  | 155.0131, [C_7_H_4_FO_3_]⁺,  (25), [-C_14_H_18_Cl] | -5.1 |
|  |  |  |  | 226.0862, [C_11_H_13_FNO_3_]⁺,  (5), [C_10_H_11_ClO] | -5.4 | \ | \ |
|  |  |  |  | 197.0598, [C_10_H_10_FO_3_]⁺,  (100), [-C_11_H_24_ClO] | -5.4 | 155.0131, [C_7_H_4_FO_3_]⁺,  (100), [-C_3_H_6_] | -5.1 |
|  |  |  |  | 155.0131, [C_7_H_4_FO_3_]⁺,  (25), [-C_14_H_20_ClO] | -5.1 | \ | \ |

***Table S12.*** List of MS^n^ product ions for TP_ha_408, *m/z* 408.1358.

**TP_ha_410**

***Figure S31.*** MS^n^ fragmentation pattern of TP_ha_410-A.

***Figure S32.*** MS^n^ fragmentation pattern of TP_ha_410-B/D.

***Figure S33.*** MS^n^ fragmentation pattern of TP_ha_410-C.

***Figure S34.*** MS^n^ fragmentation pattern of TP_ha_410-E.


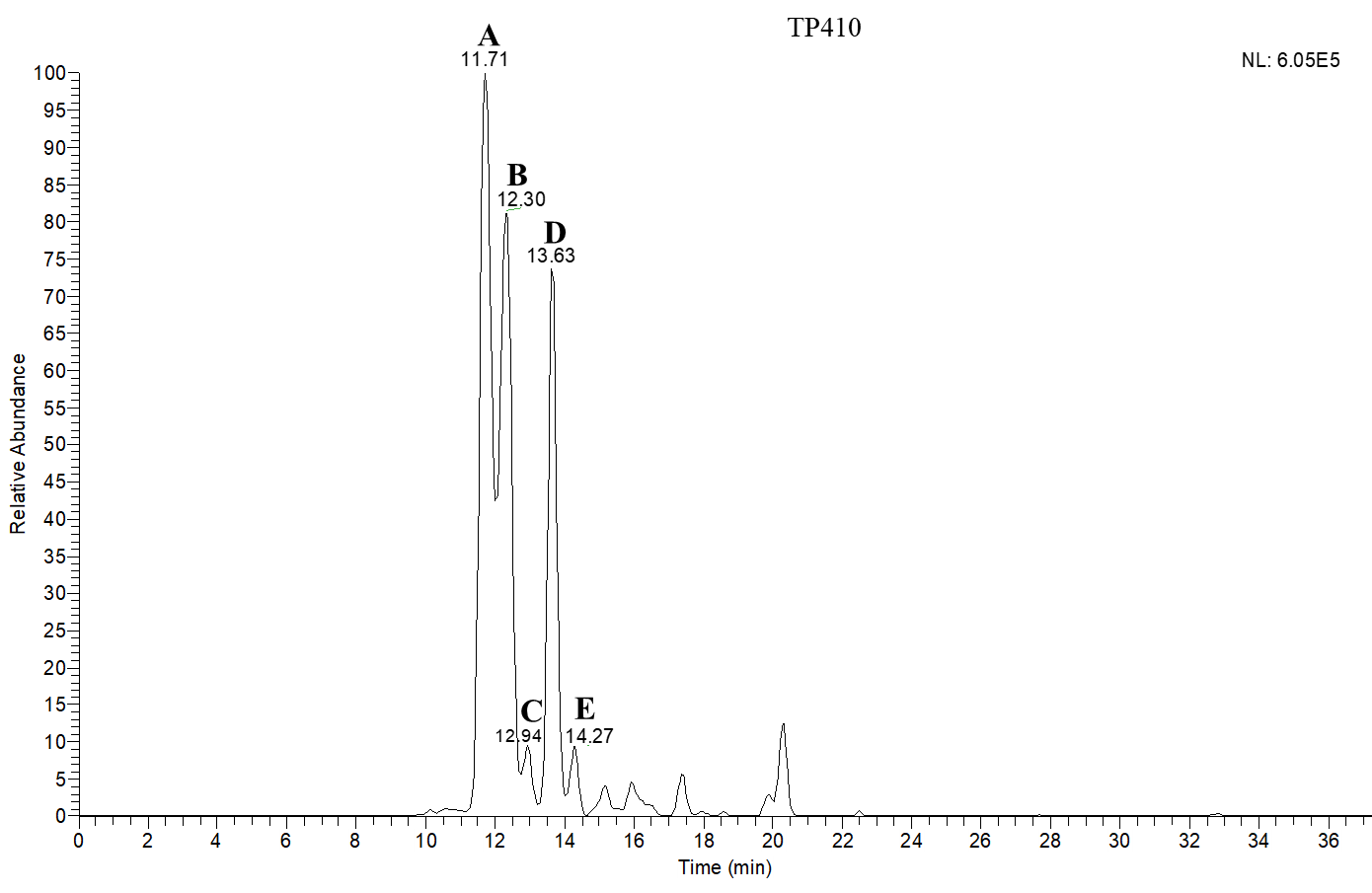


***Figure S35.*** Chromatographic separation of TP_ha_410 isomer forms, Rt= (A) 11.71, (B) 12.30, (C) 12.94, (D) 20.27 and (E) 14.27 min.

| TP_ha_ | [MH]^+^ | tr (min) | Δppm | MS^2^, molecular formula, (abundance %), [loss] | Δppm | MS^3^, molecular formula, (abundance %), [loss] | Δppm |
| --- | --- | --- | --- | --- | --- | --- | --- |
| 410-A | 410.1515  [C_21_H_26_ClFNO_4_]⁺ | 11.71 | -3.4 | 392.1407, [C_21_H_24_ClFNO_3_]⁺,  (46), [-H_2_O] | -4.1 | 374.1321, [C_21_H_22_ClFNO_2_]⁺,  (4), [-H_2_O] | -0.9 |
|  |  |  |  |  |  | 356.1639, [C_21_H_23_FNO_3_]⁺,  (100), [-HCl] | -4.9 |
|  |  |  |  |  |  | 228.0782, [C_11_H_15_ClNO_2_]⁺,  (7), [-C_10_H_9_FO] | -1.5 |
|  |  |  |  |  |  | 194.0964, [C_11_H_13_FNO]⁺,  (9), [-C_10_H_9_ClO_2_] | -2.6 |
|  |  |  |  |  |  | 165.0703, [C_10_H_10_FO]⁺,  (17), [-C_11_H_14_ClNO_2_] | -4.3 |
|  |  |  |  |  |  | 123.0235, [C_7_H_4_FO]⁺,  (3), [-C_14_H_20_ClNO_2_] | -4.6 |
|  |  |  |  | 374.1321, [C_21_H_22_ClFNO_2_]⁺,  (3), [-2H_2_O] | -0.9 | 356.1639, [C_21_H_23_FNO_3_]⁺,  (34), [-HCl] | -4.9 |
|  |  |  |  |  |  | 194.0964, [C_11_H_13_FNO]⁺,  (9), [-C_10_H_9_ClO_2_] | -3.9 |
|  |  |  |  |  |  | 165.0703, [C_10_H_10_FO]⁺,  (17), [-C_11_H_14_ClNO_2_] | -4.3 |
|  |  |  |  |  |  | 123.0235, [C_7_H_4_FO]⁺,  (3), [-C_14_H_20_ClNO_2_] | 4.6 |
|  |  |  |  | 356.1639, [C_21_H_23_FNO_3_]⁺,  (7), [-HCl] [-H_2_O] | -4.9 | 165.0703, [C_10_H_10_FO]⁺,  (100), [-C_11_H_13_NO_2_] | -4.3 |
|  |  |  |  | 264.1381, [C_15_H_19_FNO_2_]⁺,  (5), [-C_6_H_5_ClO_2_] | -5.0 | 165.0703, [C_10_H_10_FO]⁺,  (100), [-C_5_H_9_NO] | -4.3 |
|  |  |  |  | 194.0968, [C_11_H_13_FNO]⁺,  (3), [-C_10_H_13_ClO_3_] | -3.9 | 165.0703, [C_10_H_10_FO]⁺,  (100), [-CH_3_N] | -4.3 |
|  |  |  |  | 165.0703, [C_10_H_10_FO]⁺,  (100), [-C_11_H_16_ClNO_3_] | -4.3 | 123.0235, [C_7_H_4_FO]⁺,  (100), [-C_3_H_6_] | -4.6 |
|  |  |  |  | 123.0235, [C_7_H_4_FO]⁺,  (18), [-C_14_H_22_ClNO_3_] | -4.6 | \ | \ |
| 410-B | 410.1515  [C_21_H_26_ClFNO_4_]⁺ | 12.3 | -3.4 | 392.1407, [C_21_H_24_ClFNO_3_]⁺,  (46), [-H_2_O] | -4.1 | 374.1321, [C_21_H_22_ClFNO_2_]⁺,  (59), [-H_2_O] | -0.9 |
|  |  |  |  |  |  | 210.0670, [C₁₁H₁₃ClNO]⁺,  (11), [-C_10_H_11_FO_2_]⁺ | -4.8 |
|  |  |  |  |  |  | 181.0651, [C_10_H_10_FO_2_]⁺,  (16), [-C_11_H_14_ClNO] | -4.6 |
|  |  |  |  |  |  | 165.0703, [C_10_H_10_FO]⁺,  (100), [-C_11_H_14_ClNO_2_] | -4.3 |
|  |  |  |  |  |  | 123.0235, [C_7_H_4_FO]⁺,  (17), [-C_14_H_20_ClNO_2_] | -4.6 |
|  |  |  |  | 374.1321, [C_21_H_22_ClFNO_2_]⁺,  (3), [-2H_2_O] | -0.9 | 210.0670, [C₁₁H₁₃ClNO]⁺,  (92), [-C_10_H_9_FO]⁺ | -4.8 |
|  |  |  |  |  |  | 194.0968, [C_11_H_13_FNO]⁺,  (22), [-C_10_H_9_ClO] | -3.9 |
|  |  |  |  |  |  | 165.0703, [C_10_H_10_FO]⁺,  (100), [-C_11_H_12_ClNO] | -4.3 |
|  |  |  |  | 264.1381, [C_15_H_19_FNO_2_]⁺,  (2), [-C_6_H_5_ClO_2_] | -5.0 | \ | \ |
|  |  |  |  | 210.0670, [C₁₁H₁₃ClNO]⁺,  (6), [-C_10_H_13_FO_3_]⁺ | -4.8 | \ | \ |
|  |  |  |  | 199.0755, [C_10_H_12_FO_3_]⁺,  (6), [-C_11_H_14_ClNO] | -5.0 | \ | \ |
|  |  |  |  | 181.0651, [C_10_H_10_FO_2_]⁺,  (15), [-C_11_H_16_ClNO_2_] | -4.6 | \ | \ |
|  |  |  |  | 165.0703, [C_10_H_10_FO]⁺,  (100), [-C_11_H_16_ClNO_3_] | -4.3 | 123.0235, [C_7_H_4_FO]⁺,  (100), [-C_3_H_6_] | -4.6 |
|  |  |  |  | 123.0235, [C_7_H_4_FO]⁺,  (21), [-C_14_H_22_ClNO_3_] | -4.6 | \ | \ |
| 410-C | 410.1515  [C_21_H_26_ClFNO_4_]⁺ | 12.94 | -3.4 | 392.1407, [C_21_H_24_ClFNO_3_]⁺,  (40), [-H_2_O] | -4.1 | 374.1321, [C_21_H_22_ClFNO_2_]⁺,  (3), [-2H_2_O] | 0.9 |
|  |  |  |  |  |  | 356.1639, [C_21_H_23_FNO_3_]⁺,  (34), [-HCl] | -4.9 |
|  |  |  |  |  |  | 165.0703, [C_10_H_10_FO]⁺,  (17), [-C_11_H_14_ClNO_2_] | -4.3 |
|  |  |  |  | 356.1639, [C_21_H_23_FNO_3_]⁺,  (34), [-HCl] [-H_2_O] | -4.9 | \ | \ |
|  |  |  |  | 264.1381, [C_15_H_19_FNO_2_]⁺,  (16), [-C_6_H_5_ClO_2_] | -5.0 | 165.0703, [C_10_H_10_FO]⁺,  (100), [-C_5_H_9_NO] | -4.3 |
|  |  |  |  | 165.0703, [C_10_H_10_FO]⁺,  (100), [-C_11_H_16_ClNO_3_] | -4.3 | 123.0235, [C_7_H_4_FO]⁺,  (100), [-C_3_H_6_] | -4.6 |
| 410-D | 410.1515  [C_21_H_26_ClFNO_4_]⁺ | 13.63 | -3.4 | 392.1407, [C_21_H_24_ClFNO_3_]⁺,  (37), [-H_2_O] | -4.1 | 374.1321, [C_21_H_22_ClFNO_2_]⁺,  (68), [-H_2_O] | 0.9 |
|  |  |  |  |  |  | 181.0651, [C_10_H_10_FO_2_]⁺,  (29), [-C_11_H_14_ClNO] | -4.6 |
|  |  |  |  |  |  | 165.0703, [C_10_H_10_FO]⁺,  (100), [-C_11_H_14_ClNO_2_] | -4.3 |
|  |  |  |  |  |  | 123.0235, [C_7_H_4_FO]⁺,  (16), [-C_14_H_20_ClNO_2_] | -4.6 |
|  |  |  |  | 374.1321, [C_21_H_22_ClFNO_2_]⁺,  (3), [-2H_2_O] | -0.9 | 210.0670, [C₁₁H₁₃ClNO]⁺,  (57), [-C_10_H_9_FO]⁺ | 4.8 |
|  |  |  |  |  |  | 194.0968, [C_11_H_13_FNO]⁺,  (17), [-C_10_H_9_ClO] | -3.9 |
|  |  |  |  |  |  | 165.0703, [C_10_H_10_FO]⁺,  (100), [-C_11_H_12_ClNO] | -4.3 |
|  |  |  |  |  |  | 123.0235, [C_7_H_4_FO]⁺,  (15), [-C_14_H_18_ClNO] | -4.6 |
|  |  |  |  | 264.1381, [C_15_H_19_FNO_2_]⁺,  (7), [-C_6_H_5_ClO_2_] | -5.0 | \ | \ |
|  |  |  |  | 210.0670, [C₁₁H₁₃ClNO]⁺,  (5), [-C_10_H_13_FO_3_]⁺ | -4.8 | \ | \ |
|  |  |  |  | 181.0651, [C_10_H_10_FO_2_]⁺,  (24), [-C_11_H_16_ClNO_2_] | -4.6 | \ | \ |
|  |  |  |  | 165.0703, [C_10_H_10_FO]⁺,  (100), [-C_11_H_16_ClNO_3_] | -4.3 | 123.0235, [C_7_H_4_FO]⁺,  (100), [-C_3_H_6_] | -4.6 |
|  |  |  |  | 123.0235, [C_7_H_4_FO]⁺,  (20), [-C_14_H_22_ClNO_3_] | -4.6 | \ | \ |
| 410-E | 410.1515 [C_21_H_26_ClFNO_4_]⁺ | 14.27 | -3.4 | 392.1407, [C_21_H_24_ClFNO_3_]⁺,  (86), [-H_2_O] | -4.1 | 372.1347, [C_21_H_23_ClNO_3_]⁺, (100), [-HF] | -3.7 |
|  |  |  |  | 372.1347, [C_21_H_23_ClNO_3_]⁺,  (100), [-H_2_O][-HF] | -3.7 | \ |  |
|  |  |  |  | 298.1191, [C_15_H_21_ClNO_3_]⁺,  (46), [-C_7_H_5_FO] | -4.5 | \ | \ |
|  |  |  |  | 280.1087, [C_15_H_19_ClNO_2_]⁺,  (89), [-C_6_H_3_FO_2_] | -4.2 | \ | \ |
|  |  |  |  | 165.0703, [C_10_H_10_FO]⁺,  (65), [-C_11_H_16_ClNO_3_] | -4.3 | \ | \ |

***Table S13.*** List of MS^n^ product ions for TP_ha_410, *m/z* 410.1515.

**TP_ha_422**

***Figure S36.*** MS^n^ fragmentation pattern of TP_ha_422.

**
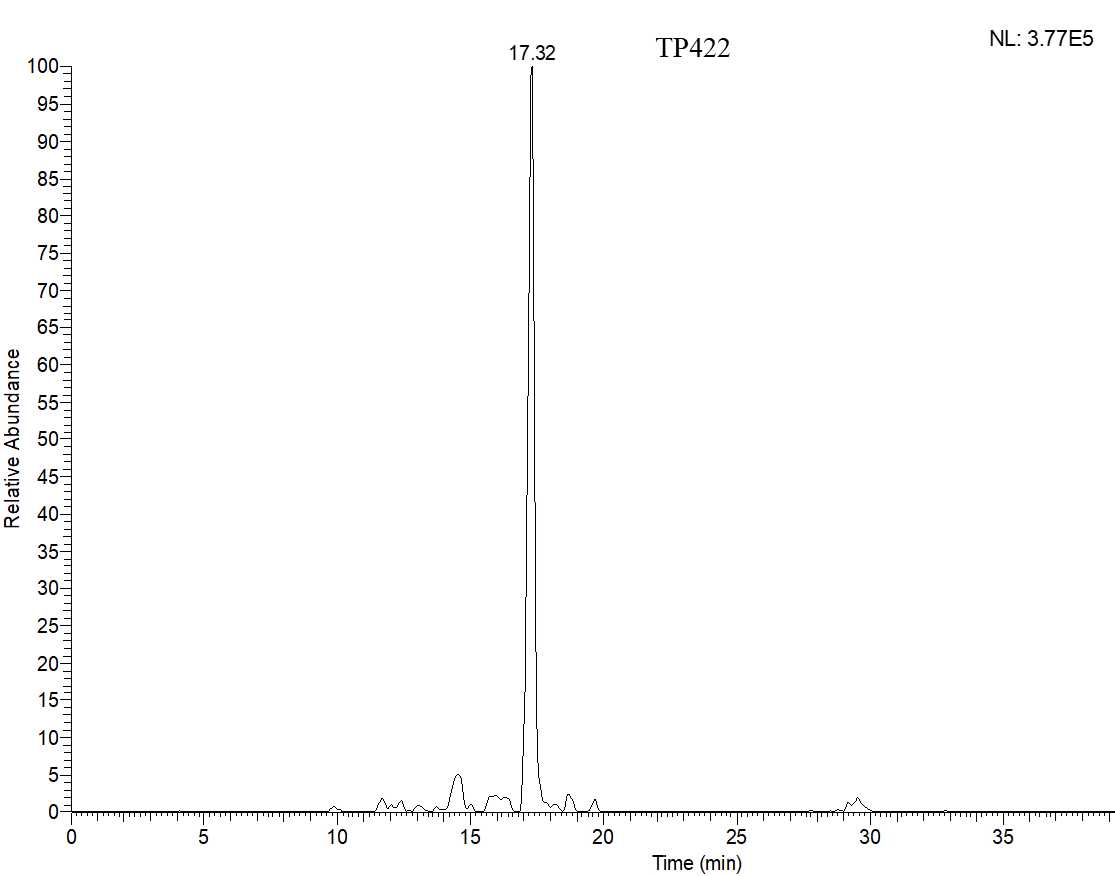
**

***Figure S37.*** Chromatographic separation of TP_ha_422, Rt= 17.32 min.

| [MH]^+^ | tr (min) | Δppm | MS^2^, molecular formula, (abundance %), [loss] | Δppm | MS^3^, molecular formula, (abundance %), [loss] | Δppm |
| --- | --- | --- | --- | --- | --- | --- |
| 422.1156  [C_21_H_22_ClFNO_5_]⁺ | 17.3 | -2.1 | 404.1049, [C_21_H_20_ClFNO_4_]⁺,  (4), [-H_2_O] | -2.6 | 240.0412, [C_11_H_11_ClNO_3_]⁺,  (1), [-C_10_H_11_FO_2_] | -4.1 |
|  |  |  |  |  | 220.1123, [C_13_H_15_FNO]⁺,  (2), [-C_8_H_7_ClO_4_] | -4.1 |
|  |  |  | 272.0676, [C_12_H_15_ClNO_4_]⁺,  (2), [-C_9_H_7_FO] | -3.0 | 254.0570, [C_12_H_13_ClNO_3_]⁺, (100), [-H_2_O] | -3.3 |
|  |  |  | 258.0519, [C_11_H_13_ClNO_4_]⁺, (2), [-C_10_H_9_FO] | -3.3 | 240.0412, [C_11_H_11_ClNO_3_]⁺,  (100), [-H_2_O] | -4.1 |
|  |  |  | 240.0412, [C_11_H_11_ClNO_3_]⁺,  (1), [-C_10_H_11_FO_2_] | -4.1 | \ | \ |
|  |  |  | 220.1123, [C_13_H_15_FNO]⁺,  (2), [-C_8_H_7_ClO_4_] | -4.2 | 165.0705, [C_10_H_10_FO]⁺,  (100), [-C_3_H_5_NO_3_] | -3.1 |
|  |  |  | 165.0705, [C_10_H_10_FO]⁺,  (100), [-C_11_H_12_ClNO_4_] | -3.1 | 123.0236, [C_7_H_4_FO]⁺,  (17), [-C_3_H_6_] | -3.8 |
|  |  |  | 123.0236, [C_7_H_4_FO]⁺,  (18), [-C_14_H_18_ClNO_4_] | -3.8 | \ | \ |

***Table S14.*** List of MS^n^ product ions for TP_ha_422, *m/z* 422.1156.

**TP_ha_424**

***Figure S38.*** MS^n^ fragmentation pattern of TP_ha_424.

**
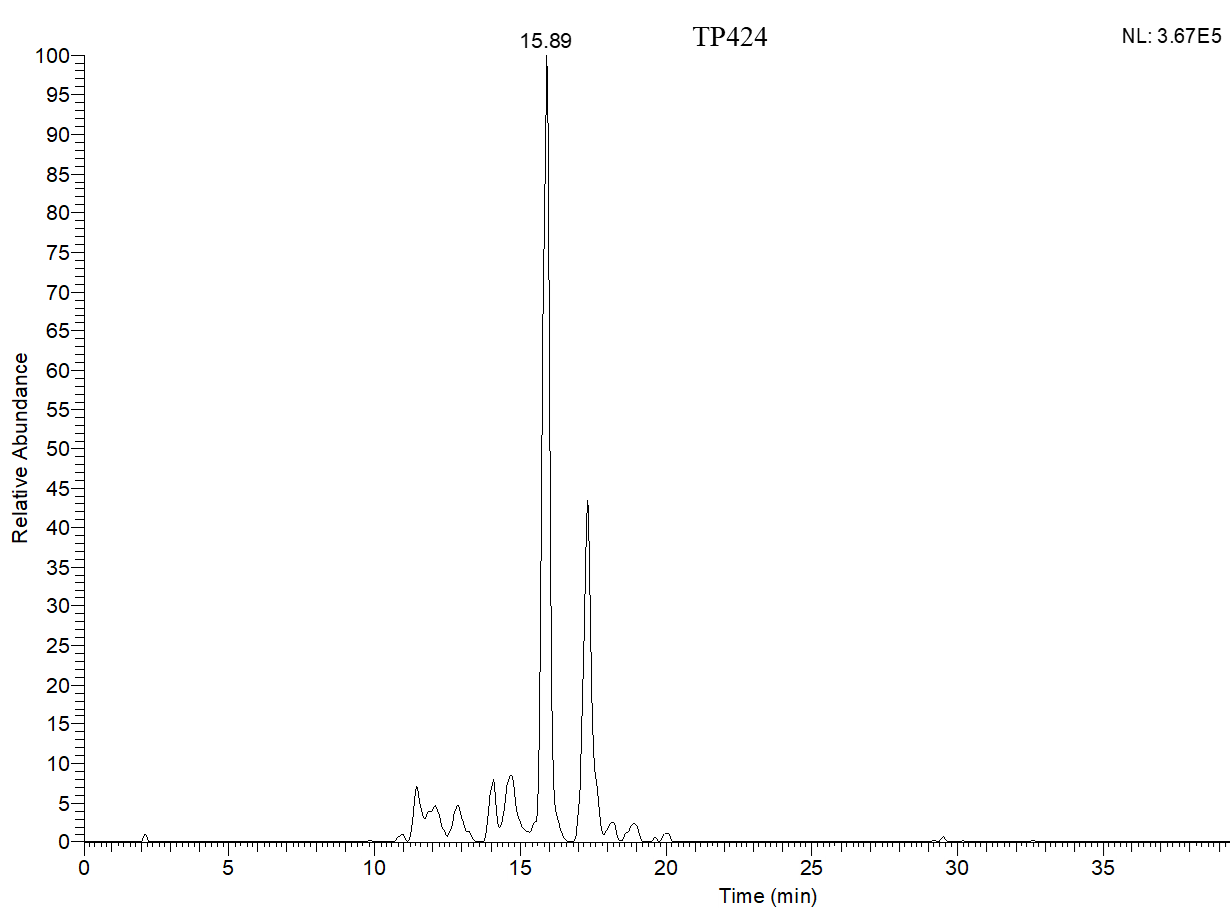
**

***Figure S39.*** Chromatographic separation of TP_ha_424, Rt = 15.89 min.

| TP_ha_ | [MH]^+^ | tr (min) | Δppm | MS^2^, molecular formula, (abundance %), [loss] | Δppm | MS^3^, molecular formula, (abundance %), [loss] | Δppm |
| --- | --- | --- | --- | --- | --- | --- | --- |
| 424 | 424.1304  [C_21_H_24_ClFNO_5_]⁺ | 15.92 | -4.1 | 406.1200, [C_21_H_22_ClFNO_4_]⁺,  (5), [-H_2_O] | -3.9 | 242.0568, [C_11_H_13_ClNO_3_]⁺,  (63) ,[-C_10_H_11_FO_2_] | -4.3 |
|  |  |  |  |  |  | 194.0966, [C_11_H_13_FNO]⁺,  (23), [-C_10_H_9_ClO_3_] | -4.9 |
|  |  |  |  |  |  | 165.0705, [C_10_H_10_FO]⁺,  (100), [-C_11_H_12_ClNO_3_] | -3.1 |
|  |  |  |  |  |  | 123.0236, [C_7_H_4_FO]⁺,  (24), [-C_14_H_20_ClNO_4_] | -3.8 |
|  |  |  |  | 260.0674, [C_11_H_15_ClNO_4_]⁺,  (10), [-C_10_H_9_FO] | -3.9 | 242.0568, [C_11_H_13_ClNO_3_]⁺,  (100) ,[-H_2_O] | -4.3 |
|  |  |  |  | 242.0568, [C_11_H_13_ClNO_3_]⁺,  (5) ,[-C_10_H_11_FO_2_] | -4.3 | \ | \ |
|  |  |  |  | 194.0966, [C_11_H_13_FNO]⁺,  (1), [-C_10_H_11_ClO_4_] | -4.9 | 165.0705, [C_10_H_10_FO]⁺,  (100), [-CH_3_N] | -3.1 |
|  |  |  |  | 165.0705, [C_10_H_10_FO]⁺,  (100), [-C_11_H_14_ClNO_4_] | -3.1 | 123.0236, [C_7_H_4_FO]⁺,  (100), [-C_3_H_6_] | -3.8 |
|  |  |  |  | 123.0236, [C_7_H_4_FO]⁺,  (17), [-C_14_H_20_ClNO_4_] | -3.8 | \ | \ |

***Table S15.*** List of MS^n^ product ions for TP_ha_424, *m/z* 424.1304.

**(a) Aripiprazole TPs with modification or loss of the tetrahydroquinolone ring**

**TP_ar_229**

***Figure S40.*** MS^n^ fragmentation pattern of TP_ar_229.

**
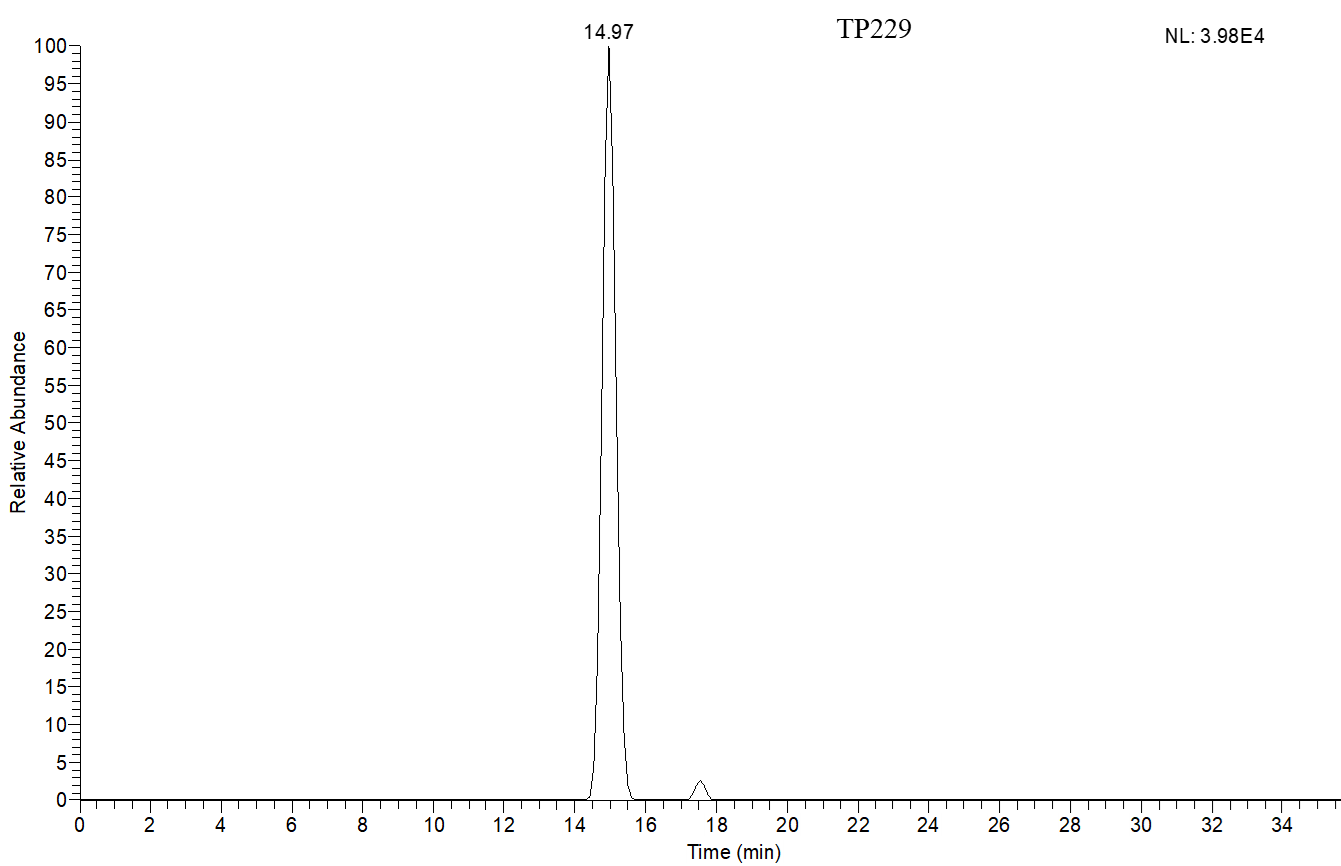
**

***Figure S41.*** Chromatographic separation of TP_ar_229, Rt = 14.97 min.

| TP_ar_ | [MH]^+^ | tr (min) | Δppm | MS^2^, molecular formula, (abundance %), [loss] | Δppm | MS^3^, molecular formula, (abundance %), [loss] | Δppm |
| --- | --- | --- | --- | --- | --- | --- | --- |
| 229 | 229.0261  [C_10_H_11_Cl_2_N_2_]⁺ | 14.97 | -1.4 | 200.0022, [C_9_H_8_Cl_2_N]⁺,  (100), [-CH_3_N] | -3.2 | 171.9715, [C_7_H_4_Cl_2_N]⁺,  (100), [-C_2_H_4_] | -0.2 |
|  |  |  |  | 173.9872, [C_7_H_6_Cl_2_N]⁺,  (24), [-C_3_H_5_N_]_ | 0.1 | \ | \ |

***Table S16.*** List of MS^n^ product ions for TP_ar_229, *m/z* 229.0261.

**TP_ar_231**

***Figure S42.*** MS^n^ fragmentation pattern of TP_ar_231.


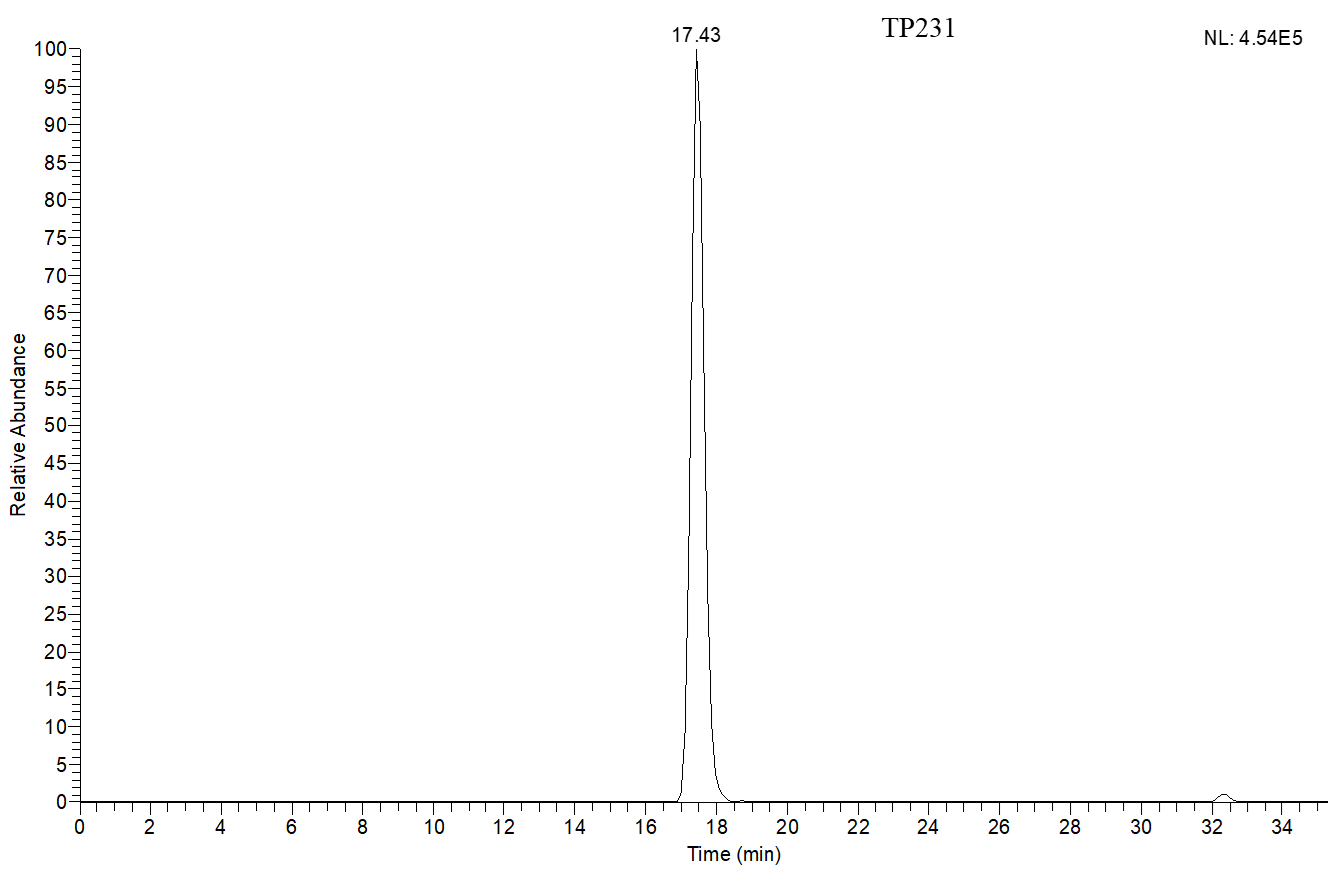


***Figure S43.*** Chromatographic separation of TP_ar_231, Rt = 17.43 min.

| TP_ar_ | [MH]^+^ | tr (min) | Δppm | MS^2^, molecular formula, (abundance %), [loss] | Δppm | MS^3^, molecular formula, (abundance %), [loss] | Δppm |
| --- | --- | --- | --- | --- | --- | --- | --- |
| 231 | 231.0443  [C_10_H_13_Cl_2_N_2_]⁺ | 17.43 | -3.1 | 200.0022, [C_9_H_8_Cl_2_N]⁺,  (1), [-CH_4_N] | -3.2 | \ | \ |
|  |  |  |  | 188.0027, [C_8_H_8_Cl_2_N]⁺,  (100), [-C_2_H_5_N] | -2.6 | 153.0336, [C_8_H_8_ClN],  (100), [-Cl°] | -2.5 |
|  |  |  |  | 153.0336, [C_8_H_8_ClN],  (8), [-C_2_H_5_N], [-Cl°] | -2.5 | \ | \ |

***Table S17.*** List of MS^n^ product ions for TP_ar_231, *m/z* 231.0443.

**TP_ar_301**

***Figure S44.*** MS^n^ fragmentation pattern of TP_ar_301.

**
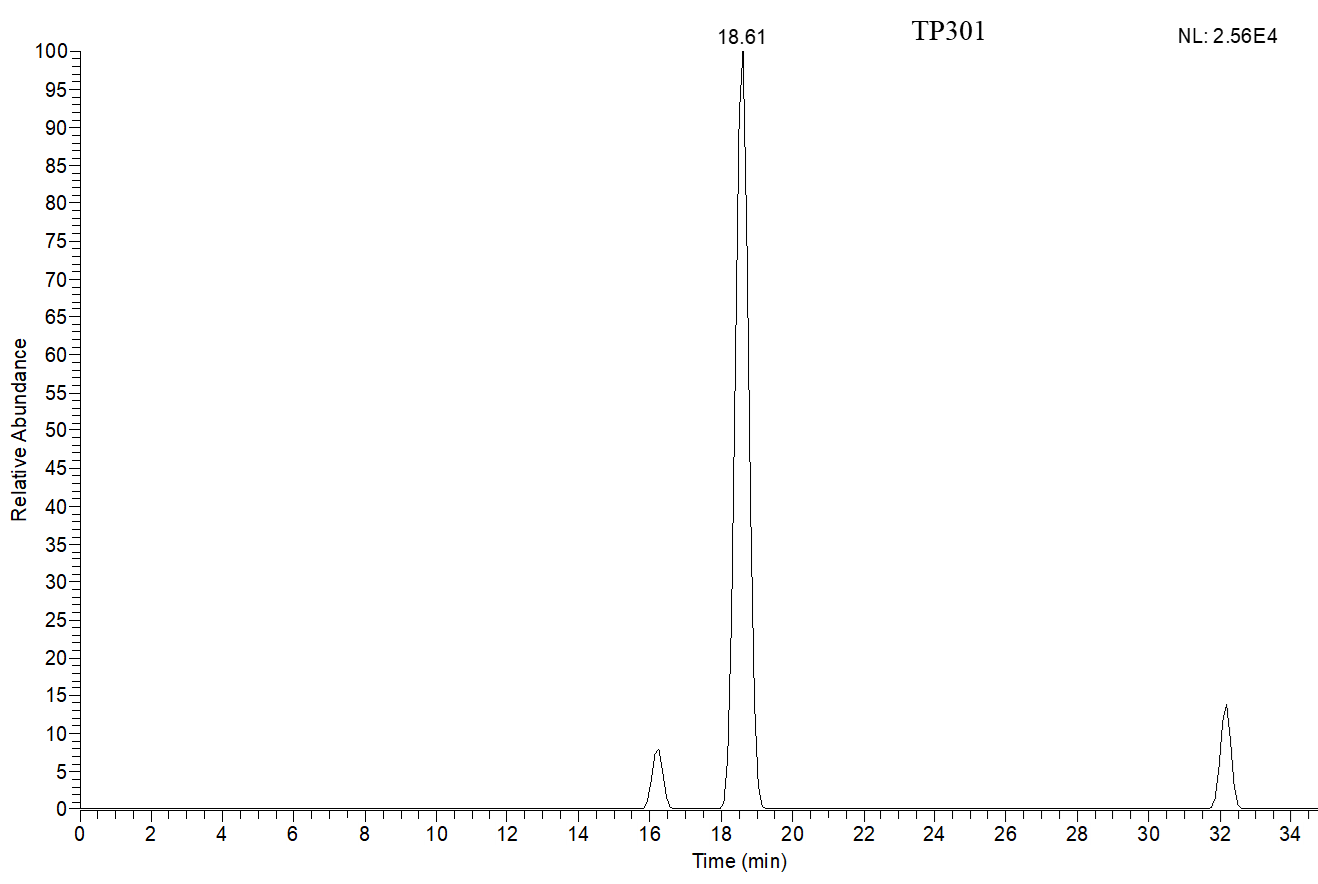
**

***Figure S45.*** Chromatographic separation of TP_ar_301, Rt = 18.61 min.

| TP_ar_ | [MH]^+^ | tr (min) | Δppm | MS^2^, molecular formula, (abundance %), [loss] | Δppm | MS^3^, molecular formula, (abundance %), [loss] | Δppm |
| --- | --- | --- | --- | --- | --- | --- | --- |
| 301 | 301.0859  [C_14_H_19_Cl_2_N_2_O]⁺ | 18.61 | -3.3 | 231.0443, [C_10_H_13_Cl_2_N_2_]⁺, (100), [-C_4_H_6_O] | -3.1 | 188.0027, [C_8_H_8_Cl_2_N]⁺,  (100), [-C_2_H_5_N] | -2.6 |
|  |  |  |  | 188.0027, [C_8_H_8_Cl_2_N]⁺,  (5), [-C_6_H_9_NO] | -2.6 | 153.0336, [C_8_H_8_ClN],  (100), [-Cl°] | -2.5 |

***Table S18.*** List of MS^n^ product ions for TP_ar_301, *m/z* 301.0859.

**TP_ar_303**

***Figure S46.*** MS^n^ fragmentation pattern of TP_ar_303.

| TP_ar_ | [MH]^+^ | tr (min) | Δppm | MS^2^, molecular formula, (abundance %), [loss] | Δppm | MS^3^, molecular formula, (abundance %), [loss] | Δppm |
| --- | --- | --- | --- | --- | --- | --- | --- |
| 303 | 303.1018  [C_14_H_21_Cl_2_N_2_O]⁺ | 18.61 | -0.03 | 285.0914, [C_14_H_19_Cl_2_N_2_]⁺,  (9), [-H_2_O] | 0.1 | 243.0450, [C_11_H_13_Cl_2_N_2_]⁺,  (44), [-C_3_H_6_] | -0.1 |
|  |  |  |  |  |  | 216.0337, [C_10_H_12_Cl_2_N]⁺,  (44), [-C_4_H_7_N] | -2 |
|  |  |  |  |  |  | 98.0962, [C_6_H_12_N]⁺,  (100) [-C_8_H_7_Cl_2_N] | -2.3 |
|  |  |  |  | 231.0443, [C_10_H_13_Cl_2_N_2_]⁺, (100),  [-C_4_H_8_O] | -3.1 | 200.0022, [C_9_H_8_Cl_2_N]⁺,  (2), [-CH_5_N] | -3.2 |
|  |  |  |  |  |  | 188.0027, [C_8_H_8_Cl_2_N]⁺,  (100), [-C_2_H_5_N] | -2.6 |
|  |  |  |  | 188.0027, [C_8_H_8_Cl_2_N]⁺,  (6), [-C_6_H_13_NO] | -2.6 | 153.0336, [C_8_H_8_ClN],  (100), [-Cl°] | -2.5 |

***Table S19.*** List of MS^n^ product ions for TP_ar_303, *m/z* 303.1018.

**TP_ar_317**

***Figure S47.*** MS^n^ fragmentation pattern of TP_ar_317.

**
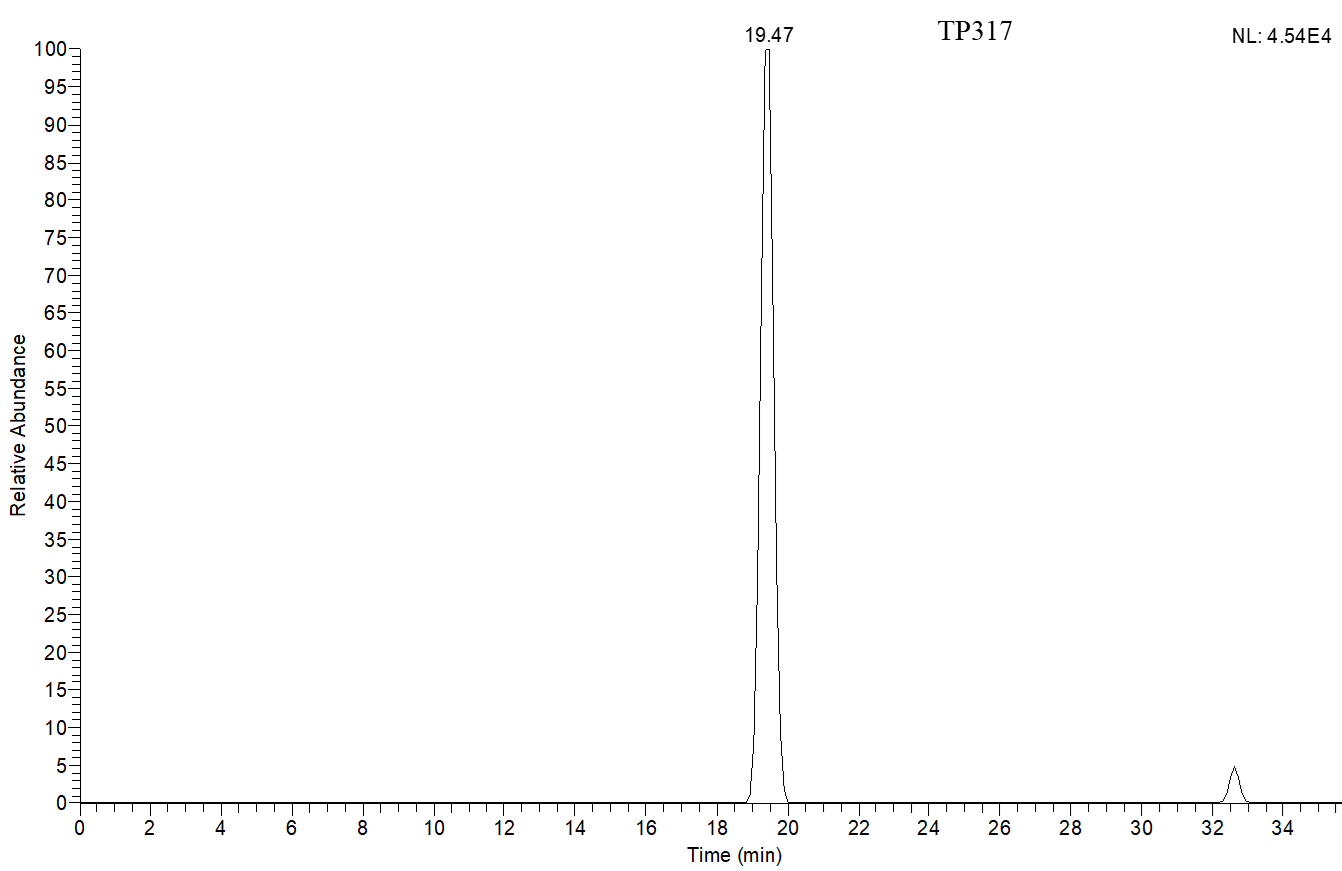
**

***Figure S48.*** Chromatographic separation of TP_ar_317, Rt = 19.47 min.

| TP_ar_ | [MH]^+^ | tr (min) | Δppm | MS^2^, molecular formula, (abundance %), [loss] | Δppm | MS^3^, molecular formula, (abundance %), [loss] | Δppm |
| --- | --- | --- | --- | --- | --- | --- | --- |
| 317 | 317.0811  [C_14_H_19_Cl_2_N_2_O_2_]⁺ | 19.47 | -2.2 | 299.0705, [C_14_H_17_Cl_2_N_2_O]⁺,  (91), [-H_2_O] | -2.5 | 281.0606, [C_14_H_15_Cl_2_N_2_]⁺,  (100), [-H_2_O_]_ | -0.3 |
|  |  |  |  |  |  | 229.0291, [C_10_H_11_Cl_2_N_2_]⁺,  (17), [_-_C_4_H_6_O] | -1.2 |
|  |  |  |  |  |  | 202.0180, [C_9_H_10_Cl_2_N]⁺,  (8), [-C_5_H_7_NO] | -2.4 |
|  |  |  |  |  |  | 126.0913, [C_7_H_12_NO]⁺,  (4), [-C_7_H_5_Cl_2_NO] | -0.3 |
|  |  |  |  |  |  | 112.0754, [C_6_H_10_NO],  (6), [-C_8_H_7_Cl_2_N] | -2.6 |
|  |  |  |  | 231.0443, [C_10_H_13_Cl_2_N_2_]⁺,  (100), [-C_4_H_6_O_2_] | -3.1 | 188.0027, [C_8_H_8_Cl_2_N]⁺,  (100), [-C_2_H_5_N] | -2.6 |
|  |  |  |  | 188.0027, [C_8_H_8_Cl_2_N]⁺,  (2), [-C_6_H_11_NO2_]_ | -2.6 | \ | \ |
|  |  |  |  | 112.0754, [C_6_H_10_NO],  (4), [-C_8_H_9_Cl_2_NO] | -2.6 | \ | \ |

***Table S20.*** List of MS^n^ product ions for TP_ar_317, *m/z* 317.0811.

**TP_ar_375**

***Figure S49.*** MS^n^ fragmentation pattern of TP_ar_375.


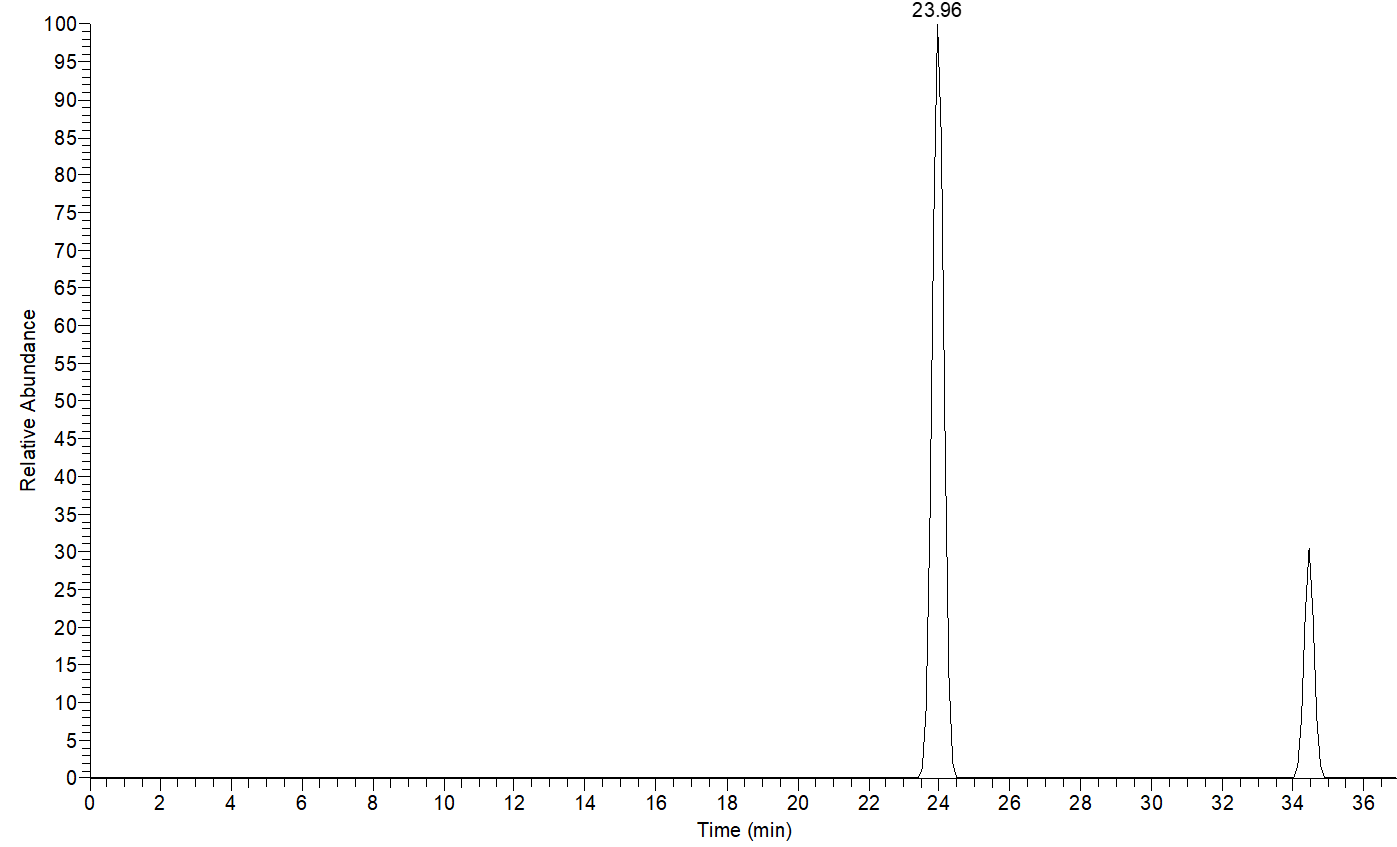


***Figure S50.*** Chromatographic separation of TP_ar_375, Rt = 23.96 min.

| TP_ar_ | [MH]^+^ | tr (min) | Δppm | MS^2^, molecular formula, (abundance %), [loss] | Δppm | MS^3^, molecular formula, (abundance %), [loss] | Δppm |
| --- | --- | --- | --- | --- | --- | --- | --- |
| 317 | 375.0865  [C_16_H_21_Cl_2_N_2_O_4_]⁺ | 23.96 | -2.3 | 331.0970, [C_15_H_21_Cl_2_N_2_O_2_]⁺,  (100), [-CO_2_] | -1.4 | 285.0914 [C_14_H_19_Cl_2_N_2_]⁺,  (100), [-CH_2_O_2_] | 0.1 |
|  |  |  |  | 285.0914, [C_14_H_19_Cl_2_N_2_]⁺,  (9), [-C_2_H_2_O_4_] | 0.1 | \ | \ |

***Table S21.*** List of MS^n^ product ions for TP_ar_375, *m/z* 375.0865.

**(b) Aripiprazole TPs with modification or loss of the dichlorobenzene ring**

**TP_ar_234**

***Figure S51.*** MS^n^ fragmentation pattern of TP_ar_234.

**
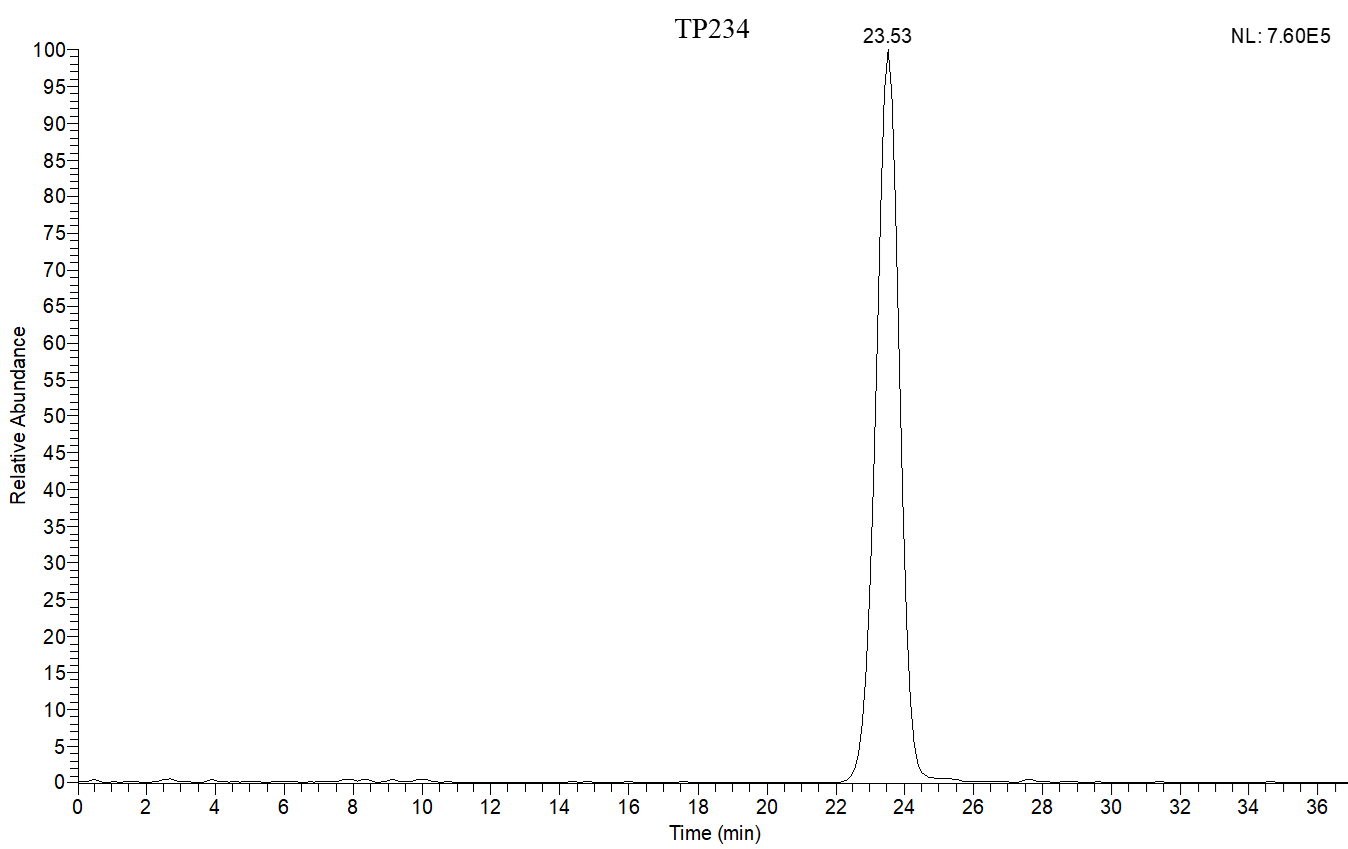
**

***Figure S52.*** Chromatographic separation of TP_ar_234, Rt = 23.53 min.

| TP_ar_ | [MH]^+^ | tr (min) | Δppm | MS^2^, molecular formula, (abundance %), [loss] | Δppm | MS^3^, molecular formula, (abundance %), [loss] | Δppm |
| --- | --- | --- | --- | --- | --- | --- | --- |
| 317 | 234.1116  [C_13_H_16_NO_3_]⁺ | 23.53 | -3.7 | 164.0700, [C_9_H_10_NO_2_]⁺,  (100), [-C_4_H_6_O] | -3.7 | 122.0964, [C_8_H_12_N]⁺,  (100), [-CH_2_O_2_] | -0.2 |

***Table S22.*** List of MS^n^ product ions for TP_ar_234, *m/z* 234.1116**.**

**TP_ar_252**

***Figure S53.*** MS^n^ fragmentation pattern of TP_ar_252.

**
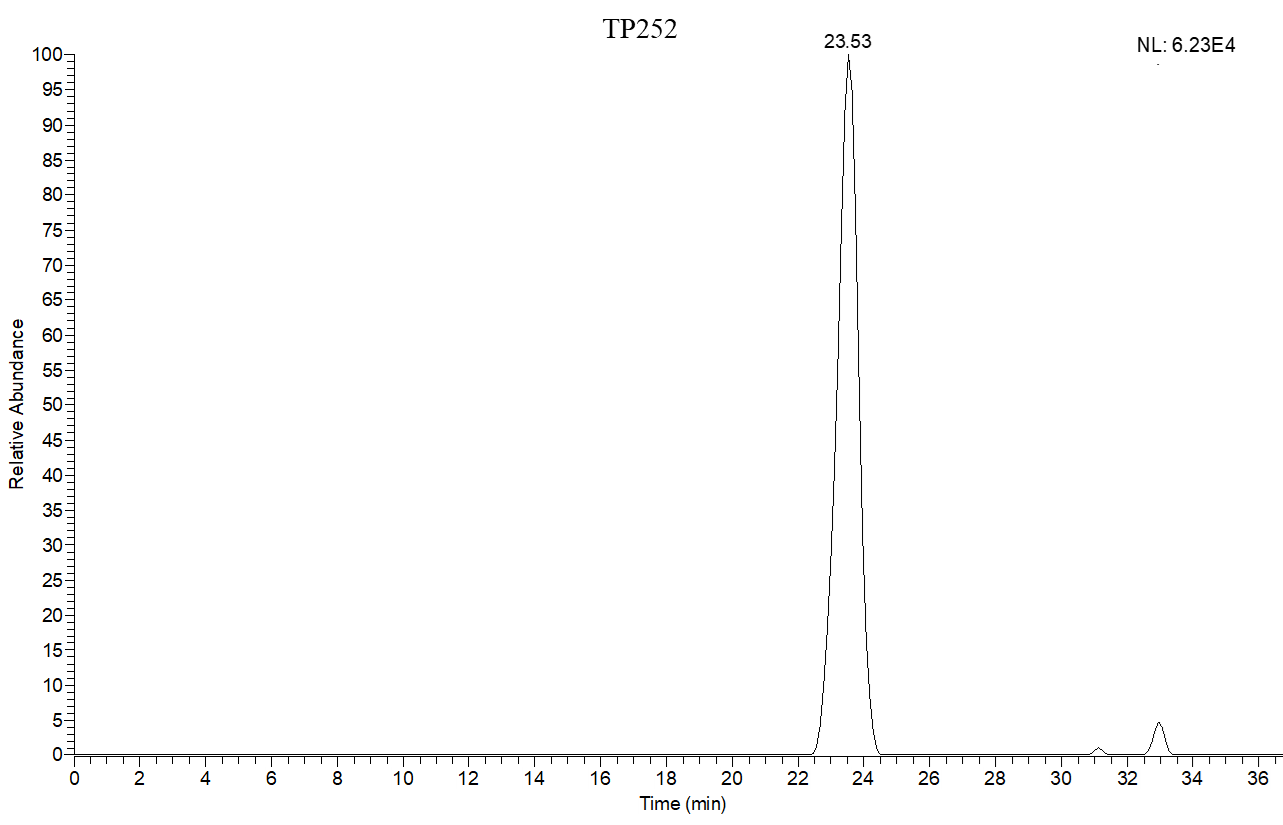
**

***Figure S54.*** Chromatographic separation of TP_ar_252, Rt = 23.53 min.

| TP_ar_ | [MH]^+^ | tr (min) | Δppm | MS^2^, molecular formula, (abundance %), [loss] | Δppm | MS^3^, molecular formula, (abundance %), [loss] | Δppm |
| --- | --- | --- | --- | --- | --- | --- | --- |
| 252 | 252.1220  [C_13_H_18_NO_4_]⁺ | 23.53 | -0.7 | 234.1118 [C_13_H_16_NO_3_]⁺,  (100), [-H_2_O] | -0.3 | 164.0701 [C_9_H_10_NO_2_]⁺,  (43), [-C_4_H_6_O] | -1.1 |
|  |  |  |  | 164.0701 [C_9_H_10_NO_2_]⁺,  (43), [-C_4_H_8_O_2_] | -0.9 | 122.0964, [C_8_H_12_N]⁺,  (100), [-CH_2_O_2_] | -0.2 |

***Table S23.*** List of MS^n^ product ions for TP_ar_252, *m/z* 252.1220.

**(c) Aripiprazole TPs with modification of the piperazine ring**

**TP_ar_422**

***Figure S55.*** MS^n^ fragmentation pattern of TP_ar_422.

**
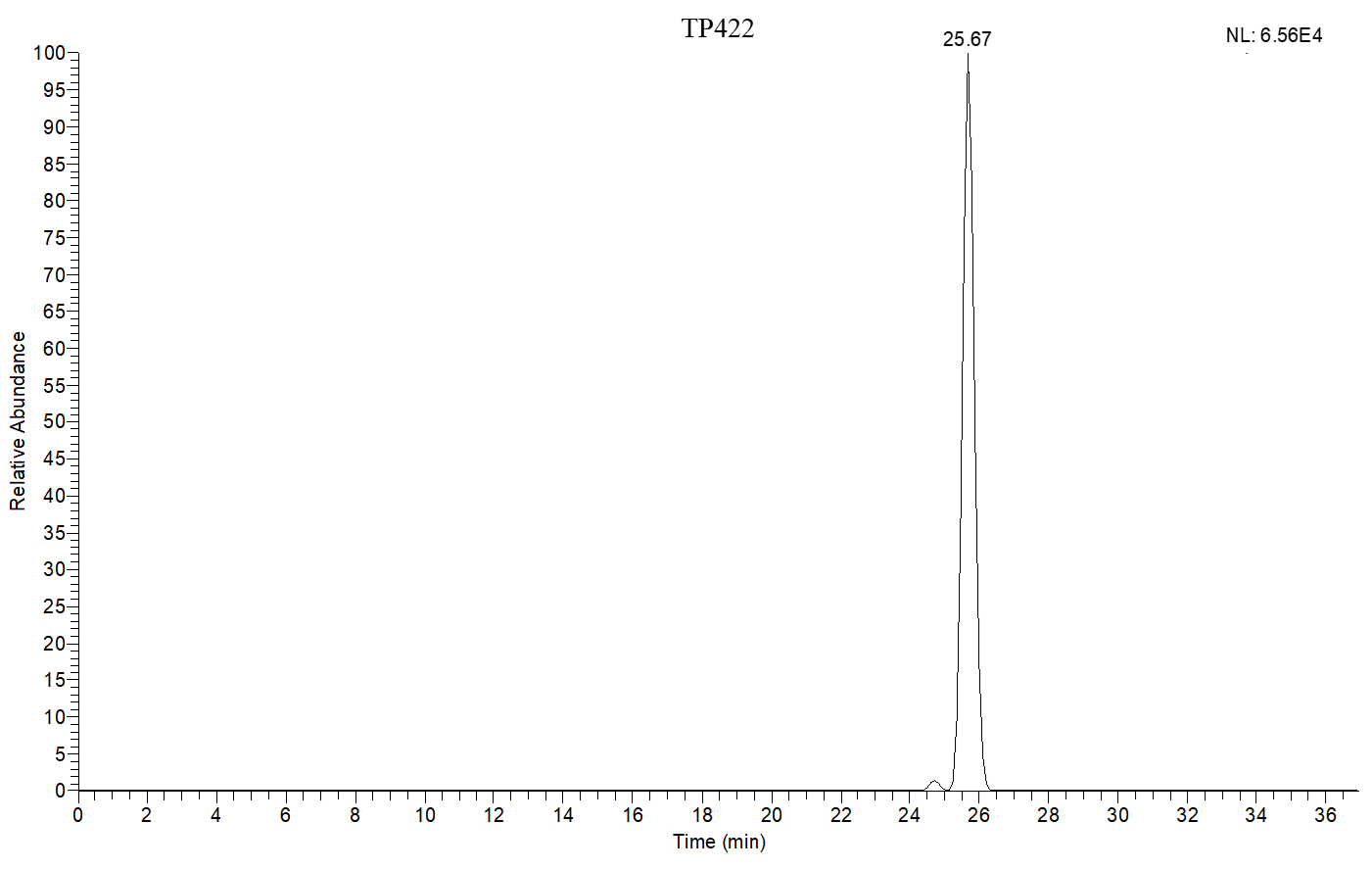
**

***Figure S56.*** Chromatographic separation of TP_ar_422, Rt = 25.67 min.

| TP_ar_ | [MH]^+^ | tr (min) | Δppm | MS^2^, molecular formula, (abundance %), [loss] | Δppm | MS^3^, molecular formula, (abundance %), [loss] | Δppm |
| --- | --- | --- | --- | --- | --- | --- | --- |
| 422 | 422.1397  [C_21_H_26_Cl_2_N_3_O_2_]⁺ | 25.67 | -0.5 | 261.1591, [C_15_H_21_N_2_O_2_]⁺,  (100), [-C_6_H_5_Cl_2_N] | 0.02 | 218.1539, [C_14_H_20_NO]⁺,  (39), [-CHNO] | -0.3 |
|  |  |  |  |  |  | 98.0962, [C_6_H_12_N]⁺,  (100) [-C_9_H_9_Cl_2_NO_2_] | -1.3 |
|  |  |  |  | 259.0757, [C_12_H_17_Cl_2_N_2_]⁺,  (34), [-C_9_H_9_NO_2_] | 0.3 | \ | \ |
|  |  |  |  | 218.1539, [C_14_H_20_NO]⁺,  (8), [-C_7_H_6_Cl_2_N_2_O] | -0.7 | \ | \ |

***Table S24.*** List of MS^n^ product ions for TP_ar_422, *m/z* 422.1397.

**TP_ar_438**

***Figure S57.*** MS^n^ fragmentation pattern of TP_ar_438.


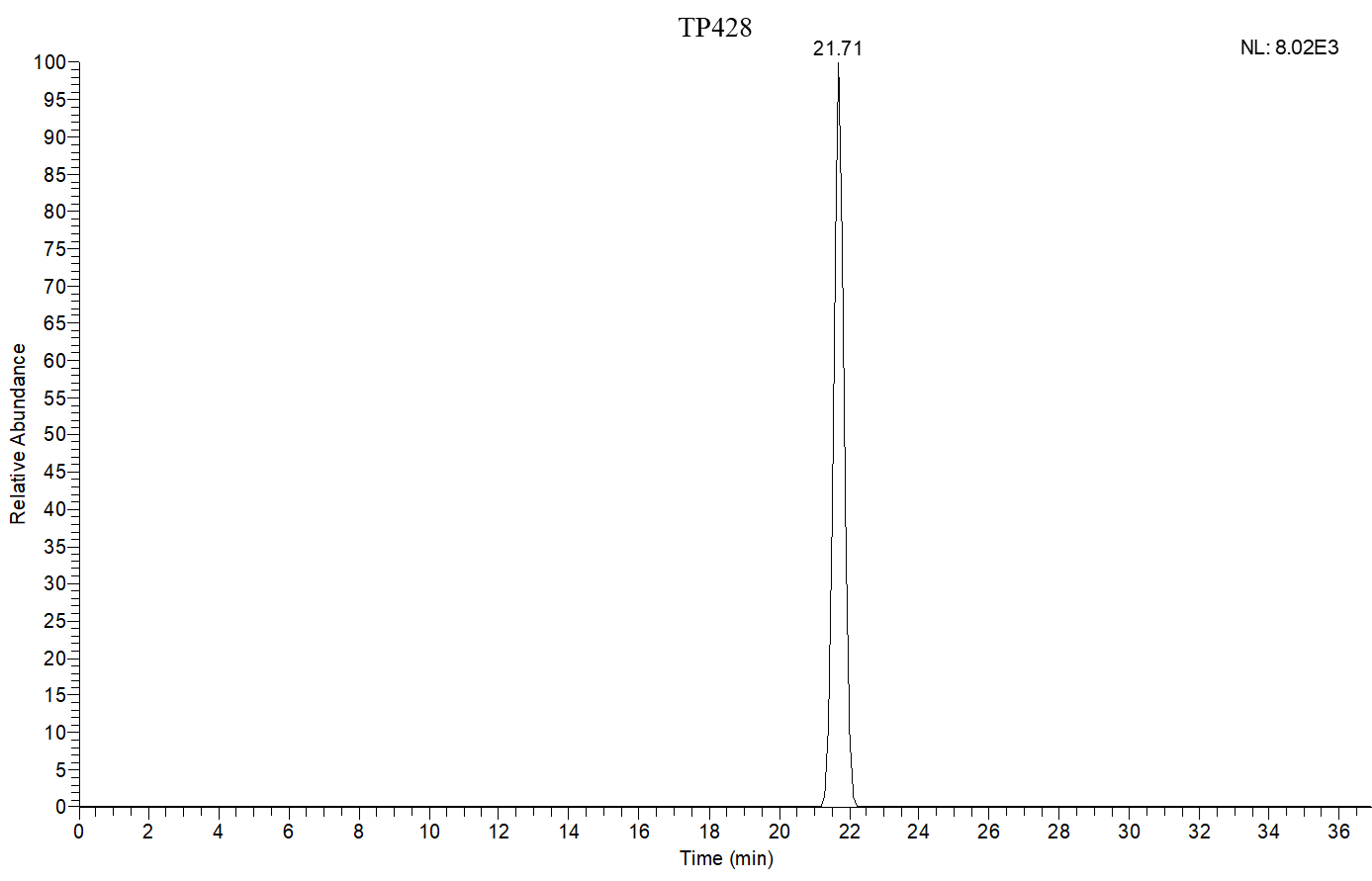


***Figure S58.*** Chromatographic separation of TP_ar_428, Rt = 21.71 min.

| TP_ar_ | [MH]^+^ | tr (min) | Δppm | MS^2^, molecular formula, (abundance %), [loss] | Δppm | MS^3^, molecular formula, (abundance %), [loss] | Δppm |
| --- | --- | --- | --- | --- | --- | --- | --- |
| 438 | 438.1334  [C_21_H_26_Cl_2_N_3_O_3_]⁺ | 21.71 | -2.7 | 420.1240, [C_21_H_24_Cl_2_N_3_O_2_],  (100), [-H_2_O] | -0.02 | 259.0763, [C_12_H_17_Cl_2_N_2_]⁺,  (43), [-C_9_H_7_NO_2_] | -0.1 |
|  |  |  |  | 259.0763, [C_12_H_17_Cl_2_N_2_]⁺,  (43), [-C_9_H_9_NO_3_] | -0.1 | \ | \ |

***Table S25.*** List of MS^n^ product ions for TP_ar_438, *m/z* 438.1334.

**(d) Aripiprazole TPs with generic structure modification**

**TP_ar_462**

***Figure S59.*** MS^n^ fragmentation pattern of TP_ar_462.

***
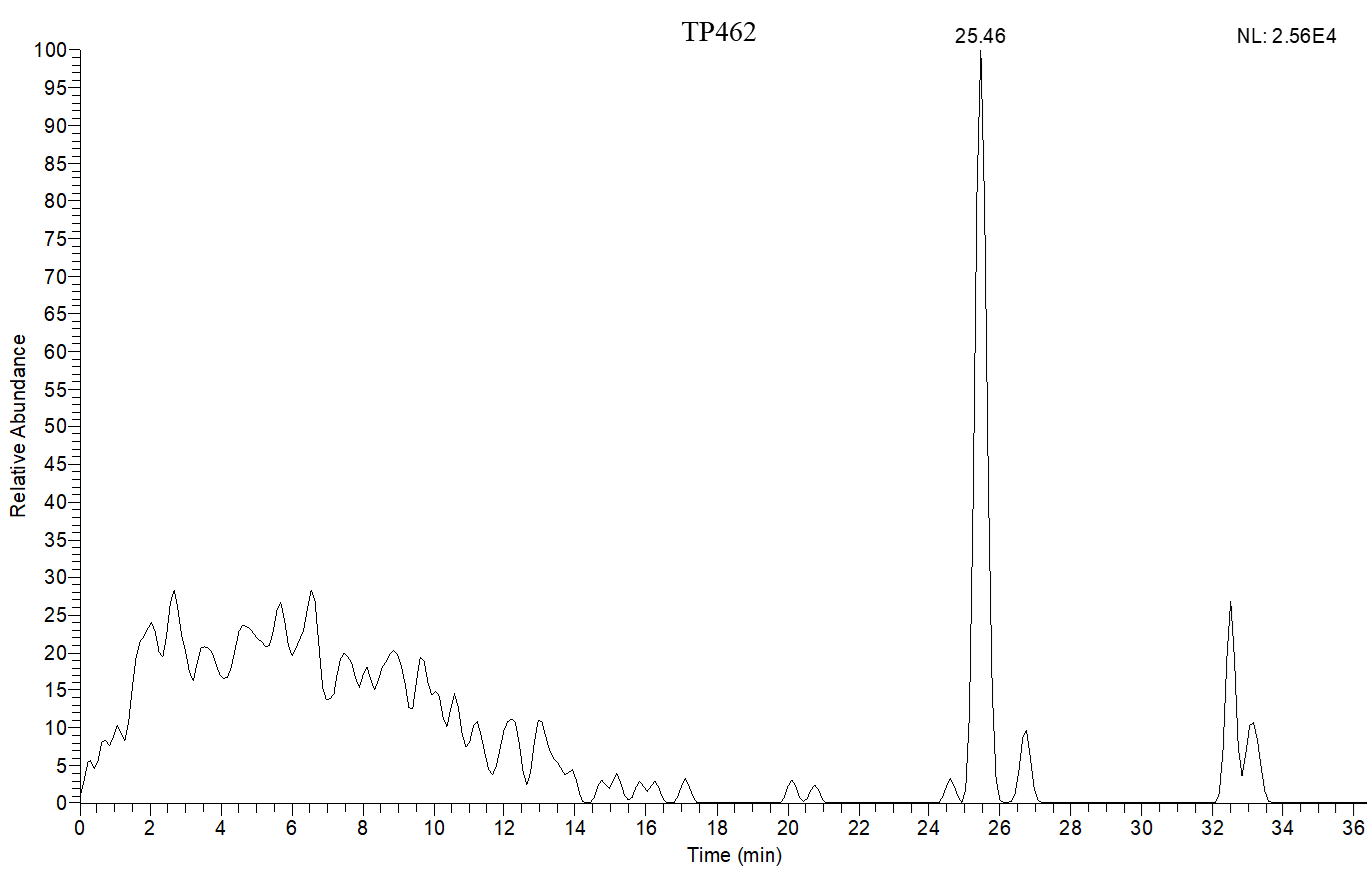
***

***Figure S60.*** Chromatographic separation of TP_ar_462, Rt =25.46 min.

| TP_ar_ | [MH]^+^ | tr (min) | Δppm | MS^2^, molecular formula, (abundance %), [loss] | Δppm | MS^3^, molecular formula, (abundance %), [loss] | Δppm |
| --- | --- | --- | --- | --- | --- | --- | --- |
| 462 | 462.1327 [C_23_H_26_Cl_2_N_3_O_3_]⁺ | 25.46 | -4.1 | 434.185, [C_22_H_26_Cl_2_N_3_O_2_]⁺,  (12), [-CO] | -2.8 | 271.0392, [C_12_H_13_Cl_2_N_2_O]⁺,  (100), [-C_10_H_13_NO] | -2.8 |
|  |  |  |  | 299.0705, [C_14_H_17_Cl_2_N_2_O]⁺, (100), [-C_9_H_9_NO_2_] | -2.5 | \ | \ |

***Table S26.*** List of MS^n^ product ions for TP_ar_462, *m/z* 462.1327

**TP_ar_464**

***Figure S61.*** MS^n^ fragmentation pattern of TP_ar_464-A.

***Figure S62.*** MS^n^ fragmentation pattern of TP_ar_464-B.

TP464

**A**

**B**

***Figure S63.*** Chromatographic separation of TP_ar_464 isomer forms, Rt = (A) 22.89 and (B) 27.59 min.

| TP_ar_ | [MH]^+^ | tr (min) | Δppm | MS^2^, molecular formula, (abundance %), [loss] | Δppm | MS^3^, molecular formula, (abundance %), [loss] | Δppm |
| --- | --- | --- | --- | --- | --- | --- | --- |
| 464-A | 464.1495  [C_23_H_28_Cl_2_N_3_O_3_]⁺ | 22.89 | -1.6 | 446.1397, [C_23_H_26_Cl_2_N_3_O_2_]⁺,  (1), [-H_2_O] | 0.1 | \ | \ |
|  |  |  |  | 285.0919 [C_14_H_19_Cl_2_N_2_]⁺,  (100), [-C_9_H_9_NO_3_] | -0.2 | 98.0962, [C_6_H_12_N]⁺,  (100) [-C_8_H_7_Cl_2_N] | -2.3 |
| 464-B | 464.1495  [C_23_H_28_Cl_2_N_3_O_3_]⁺ | 27.59 | -1.6 | 303.1021, [C_14_H_21_Cl_2_N_2_O]⁺,  (8), [-C_9_H_7_NO_2_] | -1.5 | 285.0919 [C_14_H_19_Cl_2_N_2_]⁺ (8), [-H_2_O] | -0.2 |
|  |  |  |  |  |  | 231.0450, [C_10_H_13_Cl_2_N_2_]⁺,  (100), [-C_4_H_8_O] | -0.1 |
|  |  |  |  | 285.0919 [C_14_H_19_Cl_2_N_2_]⁺,  (100), [-C_9_H_9_NO_3_] | -0.2 | 98.0962, [C_6_H_12_N]⁺,  (100) [-C_8_H_7_Cl_2_N] | -2.3 |
|  |  |  |  | 243.0450 [C_11_H_13_Cl_2_N_2_]⁺,  (30), [-C_12_H_15_NO_3_] | -0.1 | 200.0025, [C_9_H_8_Cl_2_N]⁺,  (100), [-C_2_H_5_N] | -1.6 |
|  |  |  |  | 234.1124, [C_13_H_16_NO_3_]⁺,  (9), [-C_10_H_12_Cl_2_N_2_] | -0.3 | 216.1019, [C_13_H_14_NO_2_]⁺,  (93), [-H_2_O] | -0.1 |
|  |  |  |  |  |  | 164.0703, [C_9_H_10_NO_2_]⁺,  (100), [-C_4_H_6_O] | -2.0 |
|  |  |  |  | 218.1173, [C_13_H_16_NO_2_]⁺,  (72), [-C_10_H_12_Cl_2_N_2_O] | -1.0 | 176.0704, [C_10_H_10_NO_2_]⁺,  (100), [-C_3_H_6_] | -1.3 |
|  |  |  |  |  |  | 164.0703, [C_9_H_10_NO_2_]⁺,  (22), [-C_4_H_6_] | -2.0 |
|  |  |  |  | 176.0704, [C_10_H_10_NO_2_]⁺,  (29), [-C_13_H_18_Cl_2_N_2_O] | -1.3 | 148.0756, [C_9_H_10_NO]⁺,  (100), [-CO] | -0.6 |
|  |  |  |  | 164.0703, [C_9_H_10_NO_2_]⁺,  (19), [-C_14_H_18_Cl_2_N_2_O] | -2.0 | \ | \ |

***Table S27.*** List of MS^n^ product ions for TP_ar_464, *m/z* 464.1495.
